# Systematic mining of fungal chimeric terpene synthases using an efficient precursor-providing yeast chassis

**DOI:** 10.1073/pnas.2023247118

**Published:** 2021-07-13

**Authors:** Rong Chen, Qidong Jia, Xin Mu, Ben Hu, Xiang Sun, Zixin Deng, Feng Chen, Guangkai Bian, Tiangang Liu

**Affiliations:** ^a^Key Laboratory of Combinatorial Biosynthesis and Drug Discovery, Ministry of Education, Wuhan University School of Pharmaceutical Sciences, Wuhan 430071, People’s Republic of China;; ^b^Genome Science and Technology Graduate Program, University of Tennessee, Knoxville, TN 37996;; ^c^Hubei Engineering Laboratory for Synthetic Microbiology, Wuhan Institute of Biotechnology, Wuhan 430075, People’s Republic of China;; ^d^Department of Plant Sciences, University of Tennessee, Knoxville, TN 37996

**Keywords:** chimeric terpene synthases, diterpene, sesterterpene, evolution, fungi

## Abstract

Chimeric terpene synthases, which consist of C-terminal prenyltransferase (PT) and N-terminal class I terpene synthase (TS) domains (termed PTTSs here), is unique to fungi and produces structurally diverse di- and sesterterpenes. Prior to this study, 20 PTTSs had been functionally characterized. Our understanding of the origin and functional evolution of *PTTS* genes is limited. Our systematic search of sequenced fungal genomes among diverse taxa revealed that *PTTS* genes were restricted to Dikarya. Phylogenetic findings indicated different potential models of the origin and evolution of *PTTS* genes. One was that *PTTS* genes originated in the common Dikarya ancestor and then underwent frequent gene loss among various subsequent lineages. To understand their functional evolution, we selected 74 *PTTS* genes for biochemical characterization in an efficient precursor-providing yeast system employing chassis-based, robot-assisted, high-throughput automatic assembly. We found 34 *PTTS* genes that encoded active enzymes and collectively produced 24 di- and sesterterpenes. About half of these di- and sesterterpenes were also the products of the 20 known PTTSs, indicating functional conservation, whereas the PTTS products included the previously unknown sesterterpenes, sesterevisene (1), and sesterorbiculene (2), suggesting that a diversity of PTTS products awaits discovery. Separating functional PTTSs into two monophyletic groups implied that an early gene duplication event occurred during the evolution of the PTTS family followed by functional divergence with the characteristics of distinct cyclization mechanisms.

## Significance

Chimeric terpene synthases, termed PTTSs, are a unique family of enzymes occurring only in fungi. Characterizing PTTSs is challenging due to the complex reactions they catalyze and the structural complexity of their products. Here, by devising an efficient precursor-providing yeast chassis and incorporating a high-throughput automated platform, we identified 34 active PTTSs, which was considerably more than the number of known functional PTTSs. This effective and rapid pipeline can be employed for the characterization of other PTTSs or related terpenoid biosynthetic enzymes. By systematically analyzing the presence/absence of *PTTS* genes together with phylogenetic analysis, the ancestral *PTTS* gene was inferred to have undergone duplication and functional divergence, which led to the development of two distinct cyclization mechanisms.

Terpenoids comprise the largest and most structurally diverse family of natural products (>80,000 have been characterized), and they are produced by organisms in all domains of life (1, 2). These organic chemicals play various roles in defense, regulation, and communication and have served as therapeutics, fragrances, and flavorings (3, 4). Terpenoids are biosynthesized from the universal C5 precursors, dimethylallyl diphosphate (DMAPP) and isopentenyl diphosphate (IPP), which are produced via the mevalonate and/or methyl-erythritol 4-phosphate pathways (depending on the organism). Subsequently, prenyltransferases (PTs) assemble IPP and DMAPP into linear isoprenyl diphosphate chains of various lengths. Terpene synthases (TSs) use these chains to generate a hydrocarbon core skeleton with multiple chiral centers. The core skeleton is modified by a series of enzymes to generate structurally and functionally diverse terpenoids (*SI Appendix*, Fig. S1). The structural complexity and corresponding diversity of terpenoids is primarily due to various TSs. According to the initial carbocation formation strategy, TSs are traditionally classified into a class I TS clade that generates carbocation via diphosphate ionization and a class II clade that initiates cyclization by protonating an olefinic double bond or epoxide group in an isoprenoid substrate (5–7). Class I TSs have the characteristic conserved metal-binding, Asp-rich structural motifs, DDXXD/E and (N/H)DXX(S/T)XXXE, whereas class II TSs have the signature of a general acid motif, DXDD (8, 9). PTs are generally divided into *cis*-PTs and *trans*-PTs, with *trans*-PTs containing two aspartate-rich motifs (10). Despite the absence of significant sequence similarity, *trans*-PTs and class I TSs are evolutionarily related (11), sharing a conserved structural domain known as the TS fold or alpha domain (9).

**Fig. 1.**
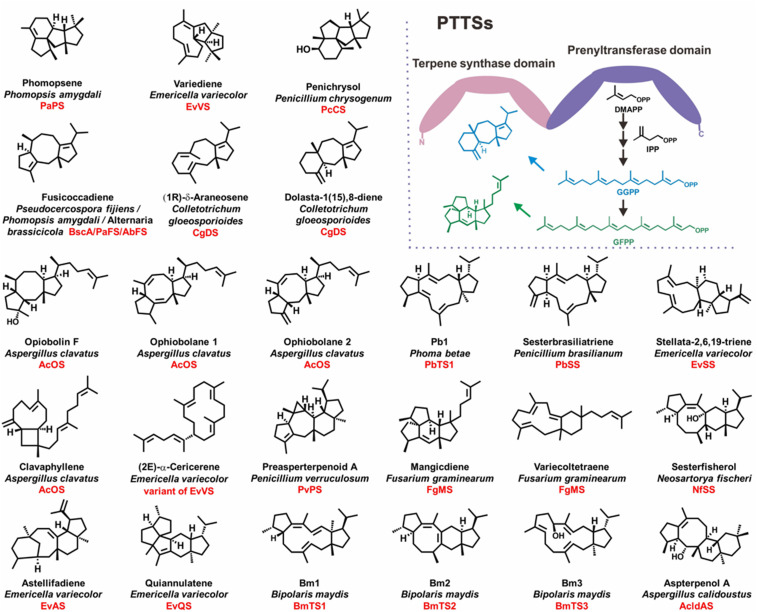
Chemical structures of di- and sesterterpenes synthesized by known fungal PTTSs and the general PTTS profile. The origin and corresponding PTTSs for di- and sesterterpenes are shown. For PTTS, the prenyltransferase domain can catalyze the condensation of IPP and DMAPP to produce GGPP and GFPP. Then, the TS domain utilizes GGPP or GFPP as a substrate to generate cyclic and acyclic di- and sesterterpenes.

Some enzymes are inherently chimeric, and the close physical proximity of their active sites can enhance product metabolic flux (12, 13). Chimeric TSs comprising a C-terminal PT domain and a class I N-terminal TS domain (PTTSs) represent a rare and important class of TSs found in various species of fungi. These PTTSs can directly use IPP and DMAPP as cosubstrates to form diverse di- and sesterterpene skeletons (14). Since the discovery of the chimeric diterpene synthase PaFS (15) and the chimeric sesterterpene synthase AcOS (16), ∼20 PTTSs and their resulting di- and sesterterpenes have been characterized using genome-based approaches (*SI Appendix*, Table S1). Notably, most compounds produced by PTTSs have novel basic carbon skeletons and complex biosynthetic mechanisms, including 5/6/3/6/5 pentacyclic preasperterpenoid A (17), 5/8/6/5 tetracyclic sesterfisherol (18), 5/5/5/6/5 pentacyclic quiannulatene (19), and 5/6/5/5 tetracyclic phomopsene (20) (Fig. 1). According to the initial carbocation formation strategy, the cyclization mechanisms of PTTSs can be classified into a type A (C1-IV-V) mode in which sequential cyclization is initiated between the C1-C15/C14-C18 of geranylfarnesyl diphosphate (GFPP) to yield a five to 15 ring system and a type B (C1-III-IV) mode in which a five to 11 ring system is uniformly generated by cyclization at the C1-C11/C10-C14 of GFPP/geranylgeranyl diphosphate (GGPP) (14, 21).

The accumulating number of PTTSs requiring functional characterization has generated several fundamental questions that await answers, such as whether *PTTS* genes of interest are widely distributed among fungi or restricted to specific groups and which mechanisms govern their functional evolution. Numerous PTTSs need to be characterized to answer these questions, and this requires an efficient high-throughput system, although *Aspergillus oryzae* is widely used for heterologous expression and it has facilitated the characterization of a series of PTTSs from filamentous fungi (18, 22). However, rapidly characterizing the functions of numerous unknown PTTSs would be impossible using this system. The development of robust terpene precursor-providing chassis in *Escherichia coli* and *Saccharomyces cerevisiae* has provided efficient approaches for the overproduction of farnesene, taxadiene, and artemisinic acid (23–25) and a scalable strategy for rapidly characterizing the functions of TSs, accelerating the process of mining novel sesqui-, di-, and sesterterpenes with unusual skeletons (26–29).

In this study, we systematically collected *PTTS* genes from various fungal species and propose an evolutionary model in which *PTTS* genes have a common origin in the Dikarya ancestor. Gene duplication and frequent gene loss are among the inferred mechanisms governing *PTTS* gene evolution. We then determined the catalytic functions of 34 PTTSs using a robot-based automatic high-throughput assembly platform and an efficient precursor-providing *S. cerevisiae* chassis. Thus, our findings provide profound insights into the origin and functional evolution of *PTTSs*, a class of TS genes that are unique to fungi.

## Results

### Mining and Phylogenetic Analysis of PTTSs.

We exhaustively searched the sequenced genomes of 477 fungi as well as genes deposited in the National Center for Biotechnology Information (NCBI) and UniProt databases. We identified 227 unique *PTTS* genes in 139 fungal species (*SI Appendix*, Fig. S2), including 20 that have already been characterized (*SI Appendix*, Table S1). Among these, 224 unique *PTTS* genes were identified in Ascomycota, and three were identified in Basidiomycota. However, genome sequencing revealed that *PTTS* genes were not ubiquitous in any fungi lineage (*SI Appendix*, Fig. S2). We identified *PTTS* genes in all Ascomycota lineages except Pezizomycetes (nine species), Saccharomycotina (40 species), and Taphrinomycotina (seven species). Within Basidiomycota, we detected *PTTS* genes in only one of the 138 species of Agaricomycotina, which contained three *PTTS* genes, but not in Ustilaginomycotina (16 species) or Pucciniomycotina (22 species). No *PTTS* genes were detected in Zygomycota (17 species) or any other basal fungal lineages (15 species).

**Fig. 2.**
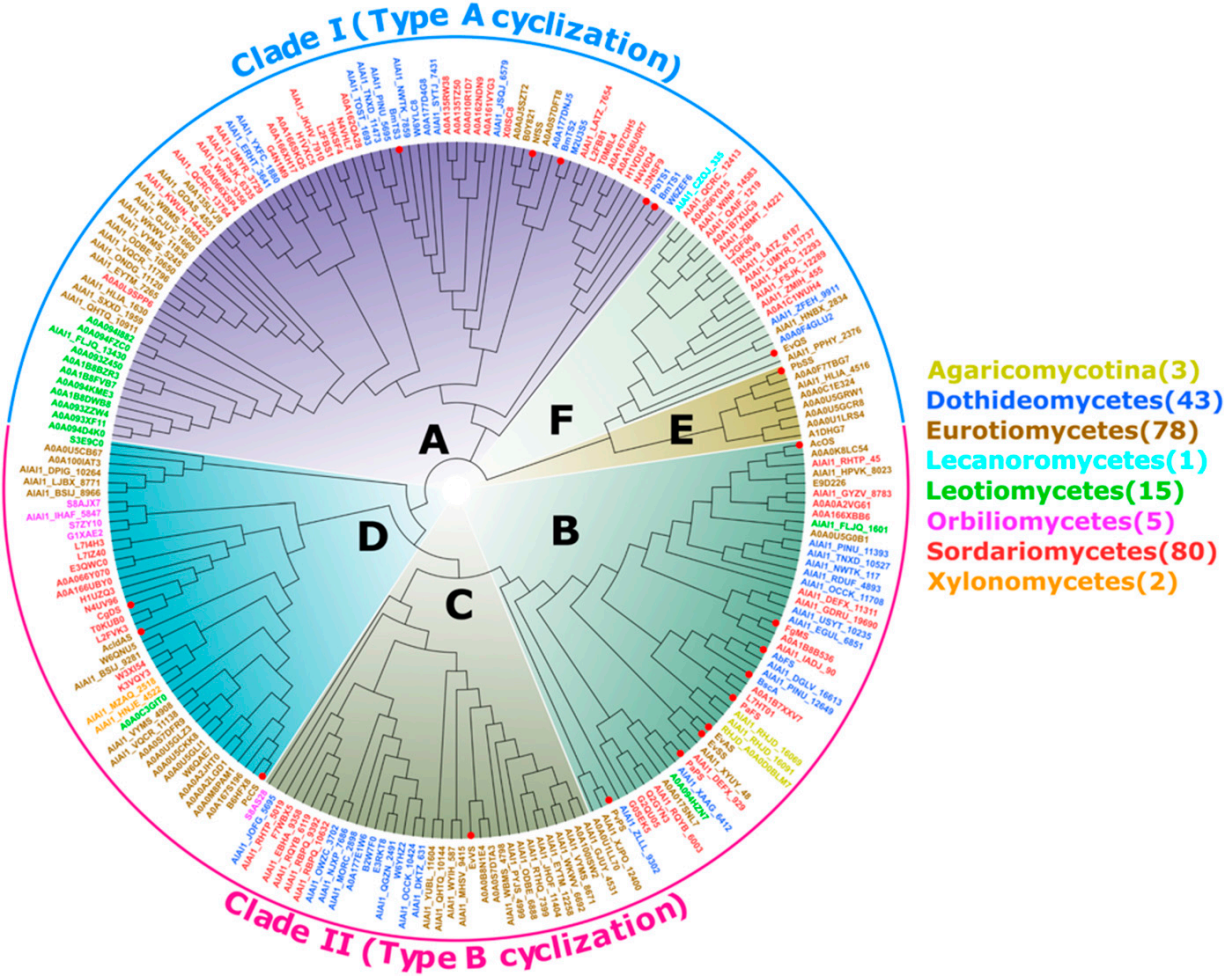
Evolutionary history of 227 PTTSs. We color coded PTTSs based on fungal lineage origin. Six subfamilies (A to F) are recognized, with subfamilies A, F, and E forming Clade I and subfamilies B, C, and D forming Clade II. The red dots represent known PTTSs.

To understand their evolutionary relatedness, a phylogenetic analysis of all 227 *PTTS* genes was performed. Prior to this study, a few studies had investigated the phylogeny of small datasets of *PTTS* genes, which led to the classification of six subfamilies (A to F) (17, 18, 21, 30). Our phylogenetic analysis using a larger and more comprehensive dataset substantiated these findings (Fig. 2), and we designated them as PTTS subfamilies A to F. Our findings also supported bifurcation of the *PTTS* genes with subfamilies A, E, and F forming Clade I and those from the remaining subfamilies forming Clade II. Known PTTSs in Clades I and II catalyze type A (C1-IV-V) and B (C1-III-IV) cyclization (14, 21), respectively. Clade II notably contains *PTTS* genes from seven lineages of fungi containing *PTTSs*, whereas Clade I contain *PTTS* genes from five lineages (Fig. 2). Within each of the six subfamilies, >90% of the *PTTS* genes remain functionally unknown, attesting to the large gap in knowledge regarding the catalytic functions of PTTSs.

### High-Throughput Mining of Active PTTSs Using an Efficient Precursor-Providing Yeast Chassis.

Compared with other classes of TSs, PTTSs are far more challenging to characterize due to the complexity of the reactions they catalyze and the structural complexity of their products. We implemented a metabolic engineering strategy to create a yeast sesterterpenoid overproduction chassis by overexpressing GFPP synthase (GFPPS) and PTTS assemblies in engineered *S. cerevisiae* YZL141 (13). To functionally characterize numerous PTTSs, we facilitated structural characterization by fusing the PTTSs to GFPPS using the linker peptide GSTGS (31) that decrease the distance between them and enhanced the metabolic flux of GFPP toward sesterterpene synthases. Introducing GFPPS can also unlock the GFPP utilization ability of chimeric diterpene synthases to generate diverse sesterterpenes.

In selecting candidate PTTSs for functional characterization, we considered amino acid (aa) sequence length (∼700 aa), sequence similarity (<80%), and the characteristics of the conserved motifs (DDXXD/E and NSE/DTE for TS and DDXXD/N for PT domains). As a result, we selected 74 candidate PTTSs. To characterize the functions of PTTSs in *S. cerevisiae*, the 74 candidates were codon optimized, synthesized, and cloned into pYJ117 to produce corresponding plasmids that were transformed using the robot-based automated high-throughput platform to obtain yeast mutants harboring *PTTS* genes (*SI Appendix*, Fig. S3). The 74 engineered strains were obtained from gene fragments within only 10 d, which greatly accelerated TS mining. The engineered *S. cerevisiae* strains were cultivated in 24-well plates for 72 h. Gas chromatography–mass spectrometry (GC-MS) results indicated that 34 of the 74 analyzed *PTTS* genes encoded active TSs, including six with only diterpene synthase, 24 with only sesterterpene, and four with bifunctional di- and sesterterpene synthase activities (*SI Appendix*, Table S3).

### Terpene Products of 34 Active PTTSs and Function–Phylogeny Relationships of Functional PTTSs.

**Fig. 3.**
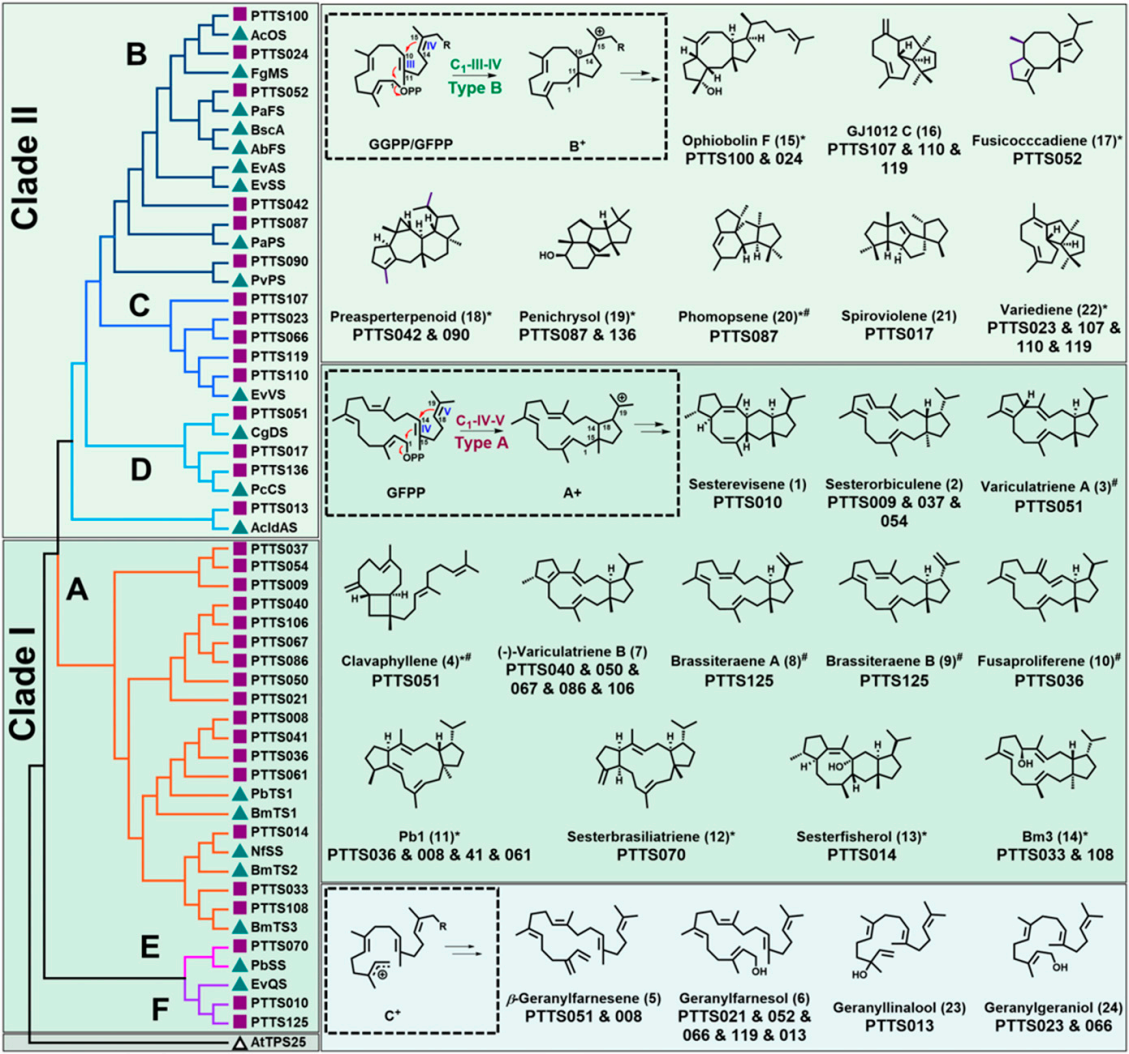
Phylogenetic analysis of characterized PTTSs and resulting products. (*Left*) Phylogenetic tree grouped 34 novel and 20 known PTTSs into six subfamilies to form Clades I (A, E, and F) and II (B to D). Accession numbers for active PTTSs are provided in *SI Appendix*, Table S3. (*Right*) Structure and cyclization models for novel products. Blue triangles represent characterized enzymes. Red squares represent enzymes assessed herein. The white triangle represents the plant-derived AtTPS25 outgroup. The “^#^” indicates structures of compounds proposed by comparing GC-MS spectra with literature. The “*” indicates structures of compounds produced by previously reported PTTSs and the newly characterized PTTSs.

The high-throughput assays showed that 34 PTTSs encoded active TSs to produce a total of 24 di- and sesterterpenes, for which titers were adequate in 18 of them. We then determined their structures via spectroscopic analysis (Fig. 3). The structures of the remaining six compounds were proposed by comparing their GC-MS spectra with reported data (*SI Appendix*, Fig. S6). Among the 24 terpene products [designated as products (**1**)–(**24**)], 20 were cyclic, four were acyclic, and 11 were the products of known PTTSs (Fig. 3). Among these, seven known sesterterpenes were produced by 12 new PTTSs, four known diterpenes were produced by eight new PTTSs, and the remaining 13 di- or sesterterpenes were generated by novel PTTS with previously unknown catalytic activities. Two of the cyclic terpenes identified as new PTTS products were novel sesterterpenes and designated as sesterevisene (**1**) and sesterorbiculene (**2**).

Among the four acyclic products, β-geranylfarnesene (**5**) (32) and geranylfarnesol (**6**) (33, 34) were sesterterpenes and geranylgeraniol (**24**) (35, 36) and geranyllinalool (**23**) (37, 38) were diterpenes (Fig. 3). These acyclic products could have resulted from ionization of the allylic diphosphate and either deprotonation of the initial carbocation or trapping with water. To exclude the possibility that these products arose from altered/misfolded PTTSs linked with GFPPS, their respective genes, PTTS013, 021, 051, 052, and 066, were overexpressed in *S. cerevisiae* as GFPPS fusions and as individual proteins. The results showed that PTTS–GFPPS fusion did not influence the production of these compounds. In fact, this strategy helped to determine the ability of the diterpene synthases PTTS013, 052, and 066 to use GFPP for geranylfarnesol production (*SI Appendix*, Fig. S7).

**Fig. 4.**
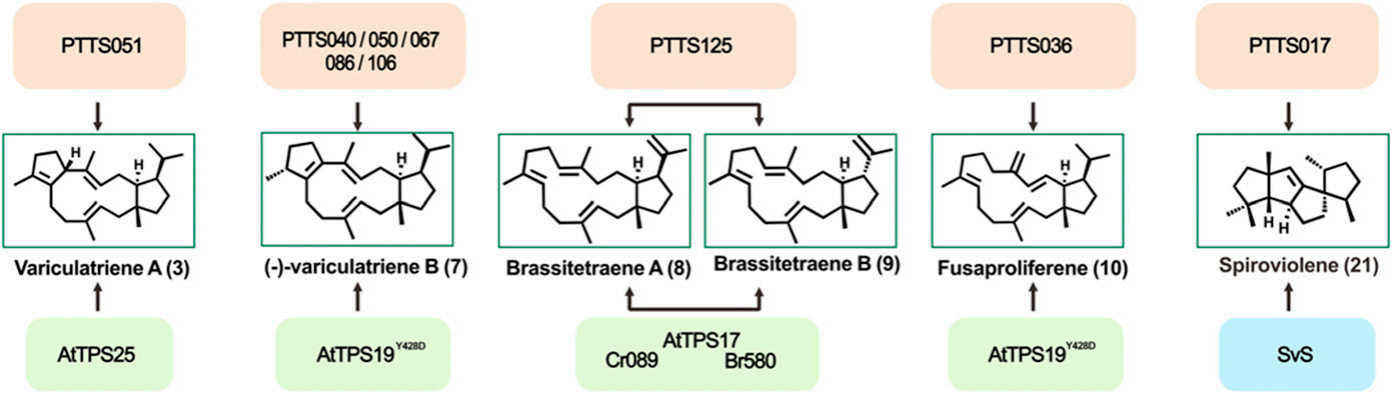
Fungal PTTSs produce five plant-derived sesterterpenes and one bacterially derived diterpene. The orange and green underlines represent fungal PTTSs characterized herein and plant-derived sesterterpene synthases, respectively. Sesterterpene synthases AtTPS25, AtTPS19^Y428D^, and AtTPS17 derived from *Arabidopsis thaliana* and sesterterpene synthases CR089 and Br580 derived from *Capsella rubella* and *Brassica rapa*, respectively. The blue underline represents bacterially derived diterpene synthase SvS from *Streptomyces violens*.

Some of the sesterterpenes have previously been shown to be produced by plants or bacteria. Previous studies have reported that plants can produce fungal-type sesterterpenes (33). Several fungal PTTSs can also produce plant and bacterial terpenes (Fig. 4). The GC-MS data showed that PTTS051 can produce variculatriene A (**3**), which is usually derived from plants, providing an additional source for the high-yield production of variculatriene A and its bioactive derivative variculanol (33, 39). Each of PTTS040, 050, 067, 086, and 106 produced the known sesterterpene (-)-variculatriene B (**7**), which is also produced by the AtTPS19^Y428D^ variant of the plant *Arabidopsis thaliana* (33). Likewise, PTTS036 produced fusaproliferene (**10**), another product of the AtTPS19^Y428D^ variant (33). PTTS125 produced brassitetraene A (**8**) and brassitetraene B (**9**), which are unified 15/5 bicyclic sesterterpene intermediates of three plant sesterterpene synthases (Cr089 from *Capsella rubella*, AtTPS17 from *A. thaliana*, and Br580 from *Brassica rapa*) (40). We thus discovered eight PTTSs that can catalyze the synthesis of plant-derived variculatriene A (**3**), (-)-variculatriene B (**7**), brassitetraene A (**8**), brassitetraene B (**9**), and fusaproliferene (**10**). The diterpene synthase PTTS017 produced spiroviolene (**21**), which is also produced by spiroviolene synthase in bacterium *Streptomyces violens* (41).

As introduced earlier, cyclic di- and sesterterpene products of PTTSs are formed through either type A (C1-IV-V) or type B (C1-III-IV) cyclization. The known enzymes catalyzing types A and B cyclization belong to two separate clades (Fig. 2). To gain further insights into this function–phylogeny relationship, we created a phylogenetic tree with the 34 new PTTSs and the 20 known PTTSs. Consistent with previous results and our findings from a larger dataset (Fig. 2), all active PTTSs were grouped into the six subfamilies (A to F; Fig. 3). Among the novel active PTTSs, 19 that located with subfamilies A, E, and F in Clade I were characterized by the C1-IV-V cyclization mode (type A). The remaining 15 PTTSs that located with subfamilies B to D in Clade II were characterized by the C1-III-IV cyclization mode (type B; Fig. 3). A number of observations were made from these results. First, the cyclic products of PTTSs from clades I and II were respectively produced through type A and type B cyclization, which was consistent with previous findings (14). Second, most of the 34 active PTTSs were single-product enzymes. Third, some closely related PTTSs had the same catalytic function, which was not surprising. For example, PTTS040, 050, 067, 086, and 106 that clustered within subfamily A, all appeared to be (-)-variculatriene B synthases. Fourth, PTTSs that shared a high identity generated different products, and those with a low identity generated similar products. For example, PTTS042 and 090, which respectively shared 36% and 94% identity with PvPS, produced the sesterterpene skeleton preasperterpenoid A (**18**) (17). PTTS023, 107, 110, and 119 shared 40 to 64% identity with EvVS and produced the same sesterterpene skeleton, variediene (**22**) (Fig. 3 and *SI Appendix*, Table S3) (42). These data suggested that only a few amino acid residues near the active pocket regulate the catalytic activity of the TS domain. The last observation was that PTTSs in Clades I and II produced acyclic products. Although such PTTSs in Clade I produced only acyclic sesterterpenes, those in Clade II produced acyclic sesterterpenes and acyclic diterpenes (Fig. 3).

### Detailed Characterization of PTTSs with Previously Unknown Sesterterpene Products.

**Fig. 5.**
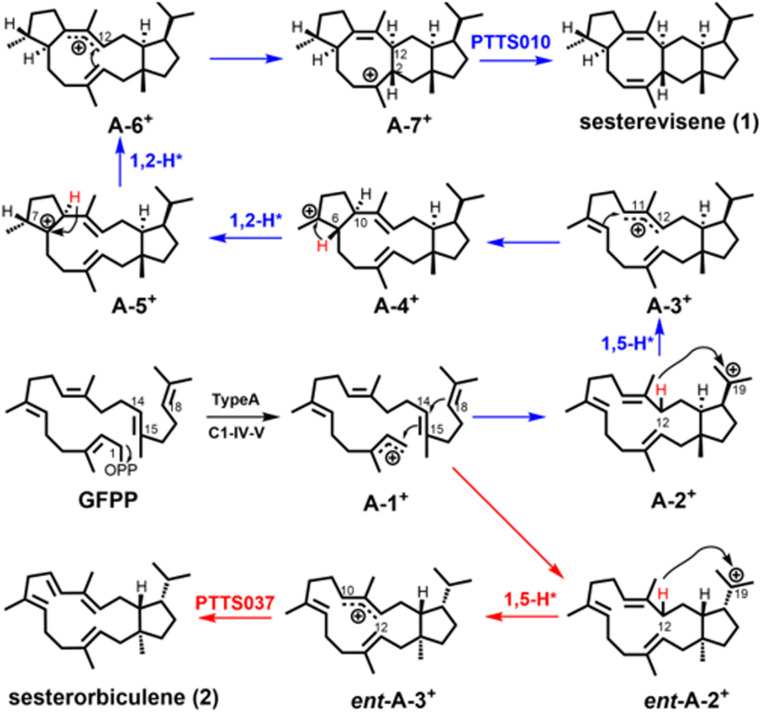
Proposed cyclization mechanisms of sesterevisene (**1**) and sesterorbiculene (**2**).

Bioinformatic data revealed that the chimeric enzyme PTTS010 (ZbSS), isolated from *Zymoseptoria brevis* and shared 46% identity with EvQS, was grouped in Clade I-F (Fig. 3). Sequence alignment indicated that the highly conserved Asp-rich motifs ^95^DDYYD and ^230^NDCHSWPKE were located in the TS domain, and the Asp-rich motifs ^487^DDIED and ^614^DDYQN were located in the PT domain. PTTS010 produced a new compound (**1**) with a characteristic sesterterpene molecular ion at *m/z* 340. Its molecular formula was confirmed as C_25_H_40_ based on a high-resolution electron ionization mass spectrometry (HR-EI-MS) ion peak [M]^+^ at *m/z* 340.3126 (calculated for 340.3130). The ^13^C NMR spectrum revealed 25 signals, including four olefinic carbons, suggesting a tetracyclic skeleton. The ^1^H and ^13^C NMR data showed the same planar structure of compound **1** (*SI Appendix*, Table S5) and aspergildiene A (43). The ^13^C NMR chemical shifts of C-6, C-17, and C-24 of **1** differed from those of aspergildiene A, indicating that the relative configuration differs between these compounds. The relative configuration of **1** was determined by NOESY correlation analysis and NMR chemical shift calculations. The NOESY correlations of H-12/H-6, H-6/H-21, H-12/H-14, H-23/H-2, and H-2/H-7 confirmed the relative configurations of C-2, C-6, C-7, C-12, C-14, and C-15 (*SI Appendix*, Fig. S8). The relative configuration of C-18 was determined by ^13^C NMR and ^1^H NMR chemical shift calculations (*SI Appendix*, Tables S15 and S16). Its absolute configuration was assigned as 2*S*, 6*S*, 7*R*, 12*S*, 14*S*, 15*R*, and 18*R* through Electronic Circular Dichroism (ECD) calculation (*SI Appendix*, Fig. S9). Thus, compound **1** was identified as a previously unknown 5/8/6/5 tetracyclic sesterterpene, named sesterevisene (**1**), and the sesterterpene synthase was designated as *Z. brevis* sesterevisene synthase (ZbSS). We proposed the following cyclization mechanism for sesterevisene (**1**) based on those of 5/8/6/5 scaffolds (Fig. 5) as follows. Initiated by the elimination of a pyrophosphate group from GFPP, two successive cyclizations at C1 to C15 and C14 to C18 yield the bicyclic cation A-2^+^ with a five to 15 fused ring system. A subsequent 1,5-H shift from C12 to C19 affords cation A-3^+^. A second C6 to C10 cyclization yields cation A-4^+^ with a tricyclic system. Two sequential 1,2-H shifts, followed by a third C2 to C12 cyclization, form the tetracyclic cation A-7^+^, producing **1** (Fig. 5).

The phylogenetic findings showed that PTTS037 (CoSS) clustered and shared 70 and 77% identity with PTTS009 and 054, respectively (*SI Appendix*, Table S3). The GC-MS data showed that PTTS009, 037, and 054 produced an identical sesterterpene hydrocarbon **2** with the formula C_25_H_40_ as determined by an HR-EI-MS [M]^+^ ion at *m/z* 340.3137 (calculated for 340.3130). Its ultraviolet (UV) absorption at 241 nm implied the presence of a conjugated double bond. The structure of **2** was subsequently elucidated by NMR spectra (*SI Appendix*, Table S6). A comparison of its ^1^H and ^13^C spectra showed that **2** was closely related to Bm3 (21). The only difference between the two structures was that **2** possessed an additional degree of unsaturation and one less hydroxyl group than Bm3, which was confirmed by heteronuclear multiple bond correlations (HMBCs) from H-9 (δ_H_ 5.45, doublet of triplets, 11.4, 8.3) to C-11 (δ_C_ 129.7), H-10 (δ_C_ 5.87, d, 11.4) to C-22 (δ_C_ 17.0), and ^1^H-^1^H COSY correlations of H_2_-8 (δ_H_ 2.76, m)/H-9-/H-10. We determined the planar structure and relative configuration of **2** by extensive two-dimensional NMR spectral analysis together with the above data (*SI Appendix*, Fig. S8). The absolute configuration of **2** was determined by the chemical elimination of Bm3 mixed with *p*-TsOH/toluene at room temperature for 10 h and the high-performance liquid chromatography (HPLC) and GC-MS detection of the conversion to **2** (*SI Appendix*, Fig. S10). Therefore, **2** was designated as a previously unknown sesterterpene and named sesterorbiculene, and the sesterterpene synthase was designated *Colletotrichum orbiculare* sesterorbiculene synthase (CoSS). Based on the cyclization mechanism of Bm3, we propose that the cyclization of **2** was initiated by sequential cyclization between the C1-cation, C14-C15 olefin (IV), and C18-C19 olefin (V) of GFPP [type A: C1-IV-V], which was consistent with the phylogenetic results (Fig. 5).

## Discussion

In this study, we systematically investigated the origin and functional evolution of PTTSs, a family of TSs that is unique to fungi. We found *PTTS* genes in Dikarya and not in any basal fungi lineages (*SI Appendix*, Fig. S2). Within Dikarya, *PTTS* genes were found in most lineages of Ascomycota but in only one of 174 species of Basidiomycota. One explanation for this is that the *PTTS* gene originated in the common ancestor of Dikarya, but genes were lost during diversification, particularly in Basidiomycota. An alternative scenario is that the single species of Basidiomycota containing *PTTSs* acquired them through other mechanisms, such as horizontal gene transfer. In the latter scenario, *PTTS* genes would have originated in Ascomycota. *PTTS* genes of Ascomycota exhibited a pattern of bifurcation, occurring in both Clade I and Clade II, except for a single gene from Lecanoromycetes, which only occurred in Clade I, and all five genes from Orbiliomycetes, which only occurred in Clade II. The absence of Clade I *PTTS* genes in Orbiliomycetes and Clade II genes in Lecanoromycetes could be caused by gene loss. As Clade II contained *PTTS* genes from a wider range of lineages than Clade I, the Clade II genes might have evolved first and an ancient duplication event led to the Clade I genes. These different models have important implications for the evolution of the catalytic mechanisms of PTTS enzymes, as they indicate that type B cyclization catalyzed by Clade II PTTSs evolved first, and type A cyclization evolved through gene duplication and functional divergence. Continued identification of *PTTS* genes from a wider range of fungal taxa and their functional characterization would provide additional evidence regarding the origin and functional evolution of *PTTS* genes.

We used a high-throughput automated platform together with an efficient yeast chassis to generate precursors and characterized 34 active fungal PTTSs that produced two new sesterterpenes and 22 known terpenes. We inferred from a comparison of their catalytic activities in the contexts of a sequence–function and phylogeny–function relationship that several mechanisms were responsible for the functional evolution of PTTSs. One such mechanism was through changes in protein sequences leading to functional divergence as evidenced by 37 different terpene products produced by 54 active PTTSs. More evidence to support this mechanism is from the identification of PTTSs producing acyclic products. These PTTSs might have evolved from a cyclic terpene-producing progenitor. A similar scenario has been proposed for typical plant TSs. The ancestral plant TS was probably a cyclase from which acyclic terpene producing enzymes evolved (44). Another possibility is that convergent evolution could have been responsible for distantly related PTTSs producing the same product. Therefore, our study not only adds to the classification of cyclization mechanisms for PTTSs that catalyze the cyclization of GGPP and GFPP to produce diverse di- and sesterterpenes but also reveals diverse mechanisms underlying their functional evolution. The precursor-providing yeast platform efficiently excavated the functions of chimeric sesterterpene synthases, enabling these observations. That 40 of the selected PTTS did not produce di-/sesterterpenes using the engineered yeast chassis is notable. Nonfunctional PTTSs might have been a consequence of incorrect exon/intron predictions, inclusion of body formation in *S. cerevisiae*, mutations in the conserved catalytic motif, or the incomplete evolution of functional genes (45–47). This requires clarification, and further investigation is needed to link the catalytic activity of the 34 active PTTSs including the acyclic terpene producers to the production of specific terpenes in the source fungus. Once such links are established, it will also be interesting to study the biological functions of these PTTSs and the sesterterpenes or diterpenes that they produce.

Interestingly, six of the terpene products of fungal PTTSs are also produced by plants or bacteria. They are produced by typical plant TSs, which are only distantly related to microbial TSs (33). The plant sesterterpene synthases are newly derived members within the TPS-a subfamily, the general function of which is sesterterpene biosynthesis (48). This presents a case of neofunctionalization. Bacterial TSs are more closely related to PTTSs, particularly the PTTS TS domain. The observation that identical terpenes can be produced by PTTSs in fungi and by classic TSs in plants/bacteria provides clear evidence of functional convergent evolution, a mechanism frequently observed in the evolution of plant secondary metabolism (49). The discovery of these PTTSs has expanded the known sources of these terpenes and provides a solid foundation for the high-yield production of terpene skeletons and their derivatives with diverse pharmacological properties.

From a methodological perspective, we developed an efficient approach for the heterologous expression of synthetic *PTTS* genes in an efficient precursor-providing yeast chassis. Numerous yeast mutants were generated from gene fragments within ∼10 d using a high-throughput automated platform. This avoided manual labor and accelerated research progress. Our findings showed that the combination of an efficient precursor-providing yeast chassis and high-throughput automation platform is an effective and rapid pipeline for characterizing PTTSs. This system can also be used to characterize other enzyme families that produce terpenes.

## Materials and Methods

### Strains and Media.

The primers used in this study are listed in *SI Appendix*, Table S23, and details on the plasmids and strains used are provided in *SI Appendix*, Tables S25 and S26. The *E. coli* strain DH10B was used to clone and propagate all plasmids. *S. cerevisiae* YZL141 was cultivated in yeast extract peptone dextrose (YPD) medium and used as a platform for the heterologous expression of PTTSs, providing the precursors IPP and DMAPP, and facilitating the overproduction of terpene products.

### Identification and Phylogenetic Analysis of Fungal PTTSs.

Two approaches were applied to identify candidate *PTTS* genes. The first was to search for putative *PTTS* genes in the 519 sequenced fungal genomes in the Joint Genome Institute (https://genome.jgi.doe.gov/programs/fungi/index.jsf) as of October 2015. Putative TSs were identified by searching all predicted amino acid sequences containing HMMER against the Pfam database (http://pfam.xfam.org, version 28.0). Sequences containing both a terpene_synth_C domain (PF03936) and a polyprenyl_synt domain (PF00348) were identified as putative *PTTS* genes. The second approach was to search for *PTTS* genes in the NCBI and UniProt databases. Putative TSs of the appropriate amino acid sequence length (∼700) with conserved motifs in their PT and TS domains were selected as candidates for functional characterization. These two sets were then combined, and all PTTSs with similarity <80% were retained. In total, the final set included 74 candidate PTTSs (*SI Appendix*, Table S2). Multiple sequence alignments were produced in MAFFT using a highly accurate setting (L-INS-i) and 1,000 iterations of improvement. Maximum likelihood phylogenetic trees were inferred with FastTree (version 2.1.7) using a high accuracy setting (-spr 4 -mlacc 2 -slownni). All trees were rendered using FigTree (version 1.4.2). To evaluate the diversity of *PTTSs* in filamentous fungi, a multiple sequence alignment was performed using CLUSTAL W 2.0.12. Evolutionary analyses were subsequently conducted using MEGA7. Finally, a phylogenetic tree was generated using the maximum likelihood method based on the Jones–Taylor–Thornton matrix-based model.

### Construction of Plasmids and Mutants.

All strains and plasmids are summarized in *SI Appendix*, Tables S26 and S27. To generate a plasmid to express sufficient levels of the precursors IPP and DMAPP in *S. cerevisiae*, pYZL141 was constructed as previously described (29). To confirm the functions of the PTTSs in *S. cerevisiae*, pRC310-pRC383 were constructed. With the help of an automated high-throughput platform (Biomek FXP Laboratory Automation Workstation), the plasmids were linearized and inserted into the HIS3 site of *S. cerevisiae* YZL141 to generate *S. cerevisiae* mRC310-mRC383. For high-throughput plasmid assembly, the LiAc/SS carrier DNA/polyethylene glycol (PEG) yeast transformation protocol was modified from a previously reported protocol (50).

For plasmid assembly, 300 ng each coding sequence fragment was combined with 300 ng linearized expression vector. Using a Biomek FX^P^ Laboratory Automation Workstation equipped with a MP200 96-Tip Tool for liquid handling operations, the DNA mix was transformed into the CEN.PK2-1D yeast strain using the modified LiAc/PEG protocol. Detailed procedures are presented in *SI Appendix*, Fig. S3.

### Fermentation of PTTSs in *S. cerevisiae*.

For product detection, RC310-RC383 mutants were cultivated in 24 deep-well plates containing YPD medium. Cultures were incubated at 30 °C for 3 d, and then compounds were extracted using hexane/ethyl acetate (4:1) and concentrated for GC-MS detection. For structural characterization, candidate mutants were activated on YPD agar plates and inoculated into 5 mL YPD (2% glucose) medium at 30 °C overnight. Next, 1% of the culture was used to inoculate a 250-mL shaker flask containing 50 mL YPD (2% glucose), which was incubated at 30 °C overnight. Finally, 1% of the culture was transferred to a 2-L shaker flask containing 1 L of YPD (2% glucose and 1% D-(+)-galactose) at 30 °C for 3 d of fermentation. Strains were collected, and products were extracted three times with hexane/ethyl acetate (4:1). The organic layers were combined, concentrated using a rotary evaporator, and dissolved in hexane for GC-MS and follow-up experiments.

### GC-MS Analysis of Terpenoids.

Terpenoids were detected by GC-MS on a Thermo TRACE GC Ultra combined with a TSQ Quantum XLS MS. The samples were injected into a TRACE TR-5MS GC column (30 m × 0.25 mm × 0.25 μm). The oven temperature was initially set at 80 °C for 1 min, increased to 220 °C at 10 °C/min, and then held at 220 °C for 15 min. The injector and transfer lines were maintained at 230 and 240 °C, respectively. The compounds were all analyzed in an *m/z* range of 50 to 500. EI mass spectra were compared with the National Institute of Standards and Technology (NIST) mass spectral library.

### Isolation and Structural Identification of Compounds.

The redissolved extracts were preseparated by silica gel (80 to 100 mesh) column chromatography with petroleum ether/ethyl acetate (100:1 to 1:1). Fractions were collected and analyzed by GC-MS for identification. The final purification was performed by preparative HPLC using an Ultimate 3000 HPLC and a SEP LC-52 with a MWD UV detector (Separation Technology Co Ltd.). The elution gradient was 90 to 100% acetonitrile from 0 to 50 min and then 100% acetonitrile from 60 to 70 min. Fragments were detected using UV light at 210 nm. The product structure was identified by GC-MS, HR-EI-MS, and NMR.

#### Sesterevisene (1).

Colorless oil; [α]_D_^27^ +3.7 (c 0.18, CHCl_3_); ^1^H NMR (CDCl_3_, 500 MHz) and ^13^C NMR (CDCl_3_, 125 MHz), *SI Appendix*, Table S5; HR-EI-MS [M]^+^ *m/z* 340.3126 (calculated C_25_H_40_^+^ for 340.3130). Infrared radiation (IR) (KBr) ν_max_ 2,958, 2,925, 2,854, 1,666, 1,463, 1,363, 1,098, 954, and 802 cm^−1^.

#### Sesterorbiculene (2).

Yellowish oil; C_25_H_40_; [α]_D_^27^ -45.1 (*c* 0.10, CHCl_3_); ^1^H NMR (CDCl_3_, 500 MHz) and ^13^C NMR (CDCl_3_, 125 MHz), *SI Appendix*, Table S6; HR-EI-MS [M]^+^ *m/z* 340.3137 (calculated 340.3130 for C_25_H_40_^+^). UV (CHCl_3_) λ_max_ (log ε) 241 (3.77) nm. Infrared radiation (IR) (KBr) ν_max_ 3,448, 2,958, 2,926, 2,854, 1,639, 1,462, 1,384, 1,101, 938, and 906 cm^−1^.

## Supplementary Material

Supplementary File

## Data Availability

Data supporting the findings of this work are included in the article and/or *SI Appendix*. The sequences of 34 biochemically characterized PTTSs reported in this paper have been deposited in the GenBank database (accession nos. MW798200 to MW798233).
