# Supplementary material for: Systematic mining of fungal chimeric terpene synthases using an efficient precursor-providing yeast chassis
Source: Proc Natl Acad Sci U S A. 2021 Jul 13;118(29):e2023247118. doi: 10.1073/pnas.2023247118 (PMC8307374; doi:10.1073/pnas.2023247118)
Supplement: Supplementary File [file pnas.2023247118.sapp.pdf]

PNAS
www.pnas.org

Supplementary Information for

Systematic mining of fungal chimeric terpene synthases using an efficient precursor-providing yeast chassis

Rong Chen[a], Qidong Jia[b], Xin Mu[a], Ben Hu[a], Xiang Sun[a], Zixin Deng[a,c], Feng Chen[b,d,*], Guangkai Bian[a,*], Tiangang Liu[a,c,*]
[a] Key Laboratory of Combinatorial Biosynthesis and Drug Discovery, Ministry of Education and Wuhan University School of Pharmaceutical Sciences, Wuhan 430071, PR China
[b] Genome Science and Technology Graduate Program, University of Tennessee, Knoxville, TN 37996, USA
[c] Hubei Engineering Laboratory for Synthetic Microbiology, Wuhan Institute of Biotechnology, Wuhan 430075, PR China
[d] Department of Plant Sciences, University of Tennessee, 2431 Joe Johnson Drive, Knoxville, TN 37996, USA

Corresponding authors: Tiangang Liu, Guangkai Bian, Feng Chen

Email: liutg@whu.edu.cn, gkbian@whu.edu.cn, fengc@utk.edu

This PDF file includes:

Supplementary methods
Figure S1 to S45
Tables S1 to S27
SI References

Supplementary Information Text

General procedures. 1D and 2D NMR data were recorded using an Agilent DD2 (400 MHz) NMR spectrometer (Santa Clara, CA, USA) and Bruker AV III (500 MHz and 600 MHz) spectrometers. $CDCl_3$ was used for NMR measurements. Chemical shifts were recorded in parts per million (ppm) downfield from tetramethylsilane and all shifts for ^{1}H-NMR were reported relative to the proton resonance (δ = 7.26 ppm) of $CHCl_3$, whereas shifts for ^{13}C-NMR were reported relative to the carbon resonance (δ = 77.16 ppm) of $CDCl_3$. The multiplicity (s = singlet, d = doublet, t = triplet, q = quartet, m = multiplet, br = broad), coupling constant (Hz), and integration were also recorded. UV spectra were obtained on a Shimadzu UV-2401PC spectrophotometer. Infrared (IR) spectra were obtained on a Bruker Tensor-27 FT-IR spectrometer using KBr pellets. Optical rotations were measured in $CHCl_3$ with JASCO P-1020 polarimeters. Column chromatography was performed with silica gel (80–100 mesh; Qingdao Marine Chemical, Inc., Qingdao, People's Republic of China). HR-EI-MS was performed on a Waters AutoSpec Premier P776. Semi-preparative HPLC was performed on an Thermo Fisher Scientific UltiMate 3000 HPLC and SEP LC-52 with a MWD UV detector (Separation (Beijing) Technology Co Ltd.) with Zorbax SB-C18 (9.4 mm × 250 mm) and Waters Xterra RP C18 (3.9 mm × 150 mm, 5 μm) columns. Fractions were monitored by thin layer chromatography. Spots were visualized with UV light (254 nm and 365 nm) and by heating silica gel plates sprayed with 10% H_2SO_4 in EtOH. All solvents used for column chromatography were distilled.

Reagents and chemicals. PCR amplification for plasmid construction was performed using PrimeSTAR GXL DNA polymerase (Takara Bio, Dalian, China). PCR primers were synthesized by GenScript. Fast Digest restriction enzymes were purchased from Thermo Fisher Scientific. The Plasmid Mini Kit, PCR Purification Kit, and Gel Extraction Kit were purchased from Axygen (Hangzhou, China). Cloning was performed in the chemically-competent *E. coli* strain DH10B (Invitrogen). Tryptone and yeast extracts for yeast fermentation were purchased from Angel Yeast (Wuhan, China).

Plasmid construction by the automated yeast assembly method. *S. cerevisiae* CEN. PK2-1D (EUROSCARF) *MATa ura3-52 trp1-289 leu2-3 112 his3Δ1 MAL2-8C SUC2* was used for all heterologous recombination experiments. For each assembled plasmid, 300 ng of each coding sequence fragment was combined with 300 ng of the linearized expression vector. The DNA mix was transformed into the CEN. PK2-1D strain using a modified LiAc/PEG protocol on a Biomek FX^{P} Laboratory Automation Workstation (Beckman Coulter) equipped with a MP200 96-Tip Tool for liquid handling operations.

The data of known compounds in this paper.

***β*-Geranylfarnesene (5)**: Colorless oil; $C_{25}H_{40}$; ^{1}H NMR ($CDCl_3$, 500 MHz) and ^{13}C NMR ($CDCl_3$, 125 MHz), see Table S7; HR-EI-MS $[M]^+$ *m/z* 340.3137 (calcd 340.3130 for $C_{25}H_{40}^+$).

Geranylfarnesol (6): Colorless oil; $C_{25}H_{42}O$; ^{1}H NMR ($CDCl_3$, 500 MHz) and ^{13}C NMR ($CDCl_3$, 125 MHz), see Table S7; HR-EI-MS $[M]^+$ *m/z* 358.3229 (calcd 358.3236 for $C_{25}H_{42}O^+$).

(-)-Variculatriene B (7): White solid; $C_{25}H_{40}$; $[\alpha]_D^{24}$ -87.9 (*c* 0.11, $CHCl_3$); ^{1}H NMR ($CDCl_3$, 500 MHz) and ^{13}C NMR ($CDCl_3$, 125 MHz), see Table S8; HR-EI-MS $[M]^+$ *m/z* 340.3129 (calcd 340.3130 for $C_{25}H_{40}^+$).

Pb1 (11): Colorless oil; $C_{25}H_{40}$; $[\alpha]_D^{22}$ +48.9 (c 0.12, $CHCl_3$); ^{1}H NMR ($CDCl_3$, 400 MHz) and ^{13}C NMR ($CDCl_3$, 100 MHz), see Table S8; HR-EI-MS $[M]^+$ *m/z* 340.3127 (calcd 340.3130 for $C_{25}H_{40}^+$).
Sesterbrasiliatriene (12): Colorless oil, $C_{25}H_{40}$; $[\alpha]_D^{24}$ -5.5 (*c* 0.19, $CHCl_3$); ^{1}H NMR ($CDCl_3$, 400 MHz) and ^{13}C NMR ($CDCl_3$, 100 MHz), see Table S9; HR-EI-MS $[M]^+$ *m/z* 341.3126 (calcd 340.3130 for $C_{25}H_{40}^+$).
Sesterfisherol (13): White solid; $C_{25}H_{42}O$; $[\alpha]_D^{26}$ -33.7 (c 0.2, $CHCl_3$); ^{1}H NMR ($CDCl_3$, 400 MHz) and ^{13}C NMR ($CDCl_3$, 100 MHz), see Table S9; HR-EI-MS $[M]^+$ *m/z* 358.3232 (calcd 358.3236 for $C_{25}H_{42}O^+$).
Bm3 (14): Colorless oil; $C_{25}H_{42}O$; $[\alpha]_D^{24}$ -27.7 (*c* 0.25, $CHCl_3$); ^{1}H NMR ($CDCl_3$, 400 MHz) and ^{13}C NMR ($CDCl_3$, 100 MHz), see Table S10; HR-EI-MS $[M]^+$ *m/z* 358.3237 (calcd 358.3236 for $C_{25}H_{42}O^+$).
Ophiobolin F (15): Colorless needles; $C_{25}H_{42}O$; $[\alpha]_D^{24}$ +21.4 (c 0.17, $CHCl_3$); ^{1}H NMR ($CDCl_3$, 500 MHz) and ^{13}C NMR ($CDCl_3$, 125 MHz), see Table S10; HR-EI-MS $[M]^+$ *m/z* 358.3230 (calcd 358.3236 for $C_{25}H_{42}O^+$).
GJ1012 C (16): Colorless oil; $C_{20}H_{32}$; $[\alpha]_D^{21}$ +3.4 (c 0.02, $CHCl_3$); ^{1}H NMR ($CDCl_3$, 600 MHz) and ^{13}C NMR ($CDCl_3$, 150 MHz), see Table S11; HR-EI-MS $[M]^+$ *m/z* 272.2499 (calcd 272.2504 for $C_{20}H_{32}^+$).
Fusicocccadiene (17): Colorless oil; $C_{20}H_{32}$; $[\alpha]_D^{21}$ +12.3 (*c* 0.24, $CHCl_3$);^{1}H NMR ($CDCl_3$, 400 MHz) and ^{13}C NMR ($CDCl_3$, 100 MHz), see Table S11; HR-EI-MS $[M]^+$ *m/z* 272.2508 (calcd 272.2504 for $C_{20}H_{32}^+$).
Preasperterpenoid (18): Colorless meddles; $C_{25}H_{40}$; $[\alpha]_D^{27}$ +126.5 (c 0.15, $CHCl_3$); ^{1}H NMR ($CDCl_3$, 500 MHz) and ^{13}C NMR ($CDCl_3$, 125 MHz), see Table S12; HR-EI-MS $[M]^+$ *m/z* 340.3125 (calcd 340.3130 for $C_{25}H_{40}^+$).
Penichrysol (19): Yellowish oil; $C_{20}H_{34}O$; $[\alpha]_D^{22}$ +6.9 (*c* 0.10, $CHCl_3$); ^{1}H NMR ($CDCl_3$, 400 MHz) and ^{13}C NMR ($CDCl_3$, 100 MHz), see Table S12; HR-EI-MS $[M]^+$ *m/z* 290.2607 (calcd 290.2610 for $C_{20}H_{34}O^+$).
Spiroviolene (21): Colorless oil; $C_{20}H_{32}$; $[\alpha]_D^{22}$ -1.09 (c 0.11, $CHCl_3$); ^{1}H NMR ($CDCl_3$, 400 MHz) and ^{13}C NMR ($CDCl_3$, 100 MHz), see Table S13; HR-EI-MS $[M]^+$ *m/z* 272.2502 (calcd 272.2504 for $C_{20}H_{32}^+$).
Variediene (22): Colorless oil; $C_{20}H_{32}$; $[\alpha]_D^{22}$ -32.8 (c 0.14, $CHCl_3$); ^{1}H NMR ($CDCl_3$, 600 MHz) and ^{13}C NMR ($CDCl_3$, 150 MHz), see Table S13; HR-EI-MS $[M]^+$ *m/z* 272.2500 (calcd 272.2504 for $C_{20}H_{32}^+$).
Geranyllinalool (23): Colorless oil; ^{1}H NMR ($CDCl_3$, 600 MHz) and ^{13}C NMR ($CDCl_3$, 150 MHz), see Table S14; HR-EI-MS $[M]^+$ *m/z* 290.2613 (calcd 290.2610 for $C_{20}H_{34}O^+$).
Geranylgeraniol (24): Colorless oil; ^{1}H NMR ($CDCl_3$, 500 MHz) and ^{13}C NMR ($CDCl_3$, 125 MHz), see Table S14; HR-EI-MS $[M]^+$ *m/z* 290.2615 (calcd 290.2610 for $C_{20}H_{34}O^+$).

Elimination reaction of Bm3

We added *p*-TsOH•H_2O (1.0 mg, 5.25 μmol) to a solution of Bm3 (2 mg, 5.58 μmol) in toluene (200 μL) at room temperature and stirred the mixture for 10 h. Saturated $NaHCO_3$ (200 μL), was added, then the mixture was extracted with ethyl acetate (500 μL). The organic layers were concentrated *in vacuo*. Crude extracts dissolved with ethyl acetate (200 μL) were analyzed using HPLC and GCMS.

Computational method

An exhaustive conformation space search of two diastereoisomers of 1 was conducted by CREST (version 2.8) using the default iMTD-GC procedure (1). The DFT geometry of conformers within an energy window of 5 kcal/mol (9 and 10 for 1-18*S* and 1-18*R*, respectively) were optimized at M06-2X-D3 / def2-SVP level of theory in the gas phase in Gaussian 09 (2), and NMR shielding tensors for optimized conformers with a Boltzmann distribution > 1% were further calculated at the MPW1PW91/6-31+G (d, p) level with the PCM model (chloroform). The calculated shielding constants were converted into chemical shifts by reference to TMS at 0 ppm (δcal = σTMS – σcal), where the σTMS was the shielding constant of TMS calculated at the same level. The DP4+ probability of each candidate was calculated as described using an Excel file provided by Sarotii, *et al.* (3). To calculate theoretical electronic circular dichroism (ECD), two optimized conformers of 1-18*R* in the results were determined by ESD 09 software using TDDFT at the cam-b3lyp/def2svp level with PCM in methanol. The ECD spectrum of 1-18*R* was obtained by weighing the Boltzmann distribution rate of geometric conformation in Multiwfn 3.8 (4).

The ECD spectrum was simulated by overlapping Gaussian functions for each transition according to:

$$\Delta\varepsilon(E) = \frac{1}{2.297\times10^{-39}} \times \frac{1}{\sqrt{2\pi\sigma}} \sum_i^A \Delta E_i R_i \mathrm{e}^{-[(E-E_i)/(2\sigma)]^2},$$

where σ represents the width of the band at 1/e height, and ΔE_i and R_i are the excitation energies and rotational strengths for transition i, respectively; σ = 0.66 eV and R^{velocity}.

The optimized conformation geometries, thermodynamic parameters, and populations of all conformations are provided in Tables S15–S24

Supplementary Fig.S:

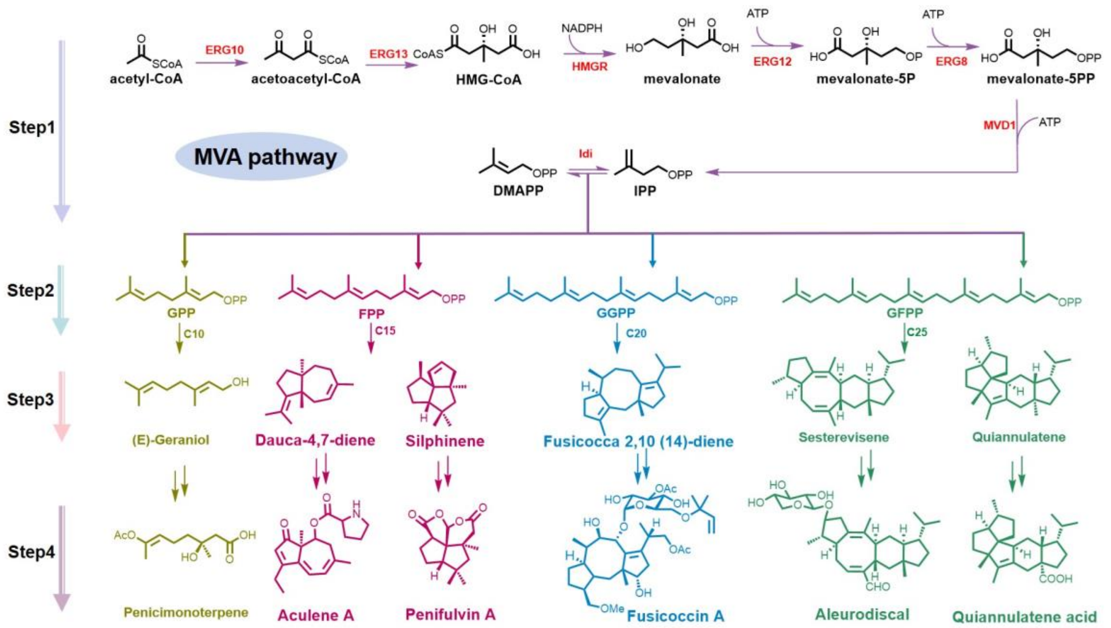

Fig. S1. The MVA pathway of terpenoids biosynthesis. Abbreviations: acetyl-CoA acetyltransferase (ERG10), 3-hydroxy-3-methylglutaryl CoA synthase (ERG13), 3-hydroxy-3-methylglutaryl CoA reductase (HMGR), mevalonate kinase (ERG12), phosphomevalonate kinase (ERG8), mevalonate diphosphate decarboxylase (MVD1), isopentenyl diphosphate isomerase (Idi), C10 geranyl dipho-sphate (GPP), C15 farnesyl diphosphate (FPP), C20 geranylgeranyldiphosphate (GGPP), and C25 geranylfarnesyl diphosphate (GFPP).

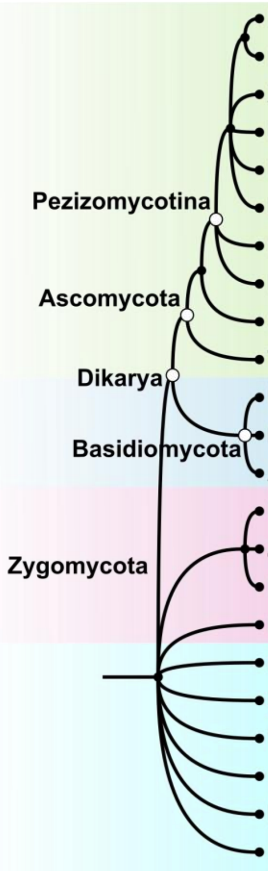

	JGI	UniProt	Known
Sordariomycetes	28(28/19/19/72)	48(25)	4(3)
Leotiomycetes	2(2/1/1/18)	13(3)	0(0)
Xylonomycetes	2(2/2/2/3)	0(0)	0(0)
Lecanoromycetes	1(1/1/1/2)	0(0)	0(0)
Eurotiomycetes	37(41/22/26/53)	31(20)	10(7)
Dothideomycetes	27(30/21/22/85)	10(7)	6(4)
Orbiliomycetes	1(1/1/1/2)	4(2)	0(0)
Pezizomycetes	0(0/0/0/9)	0(0)	0(0)
Saccharomycotina	0(0/0/0/34)	0(0)	0(0)
Taphrinomycotina	0(0/0/0/7)	0(0)	0(0)
Ustilaginomycotina	0(0/0/0/16)	0(0)	0(0)
Pucciniomycotina	0(0/0/0/22)	0(0)	0(0)
Agaricomycotina	3(3/1/1/124)	0(0)	0(0)
Kickxellomycotina	0(0/0/0/3)	0(0)	0(0)
Zoopagomycotina	0(0/0/0/0)	0(0)	0(0)
Mucoromycotina	0(0/0/0/9)	0(0)	0(0)
Entomophthoromycotina	0(0/0/0/2)	0(0)	0(0)
Cryptomycota	0(0/0/0/1)	0(0)	0(0)
Blastocladiomycota	0(0/0/0/1)	0(0)	0(0)
Neocallimastigomycota	0(0/0/0/2)	0(0)	0(0)
Glomeromycota	0(0/0/0/1)	0(0)	0(0)
Microsporidia	0(0/0/0/8)	0(0)	0(0)
Chytridiomycota	0(0/0/0/2)	0(0)	0(0)
	101(108/68/73/476)	106(57)	20(14)

Fig. S2. Analysis of *PTTS* genes in sequenced fungal genomes deposited at Joint Genome Institute (JGI; https://mycocosm.jgi.doe.gov/mycocosm/home) and other fungi from NCBI and UniProt (UniProt). The numbers outside the parentheses indicate the number of unique *PTTS* genes identified from a specific lineage. In the JGI column, the four numbers in each indicate total number of *PTTS* genes; total number of fungal species containing *PTTS* genes, total number of strains containing *PTTS* genes analyzed, and total number of species analyzed, respectively. In the UniProt column, the number in each parentheses indicates the total number of fungal species that were found to contain *PTTS* genes.

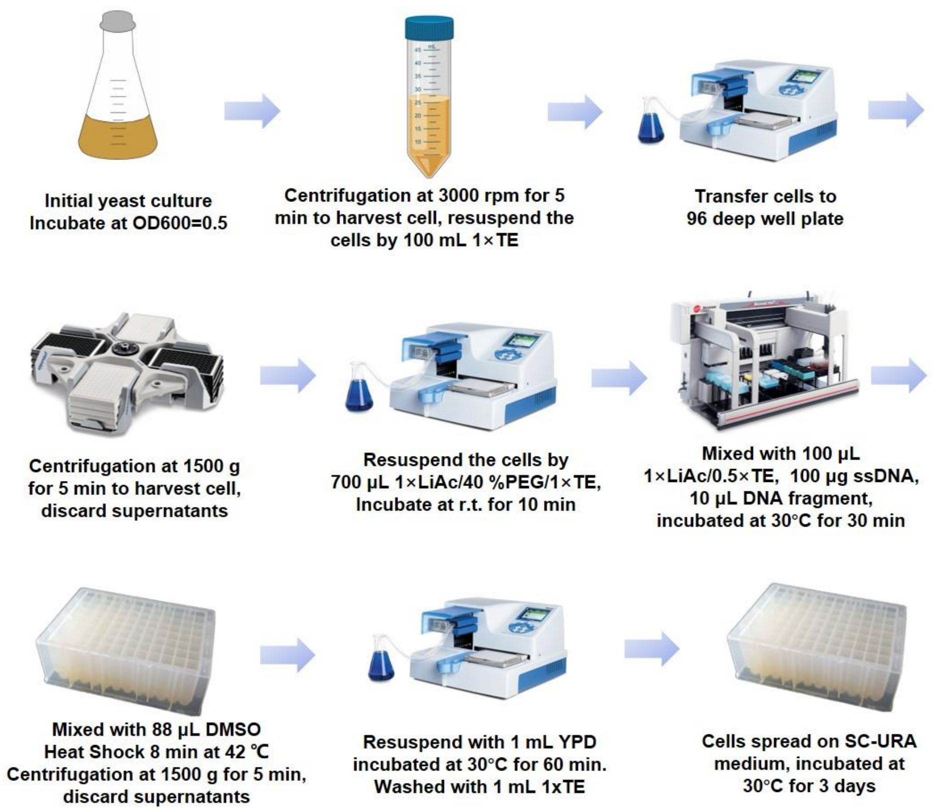

Fig. S3. Schematic illustration of high-throughput yeast assembly.

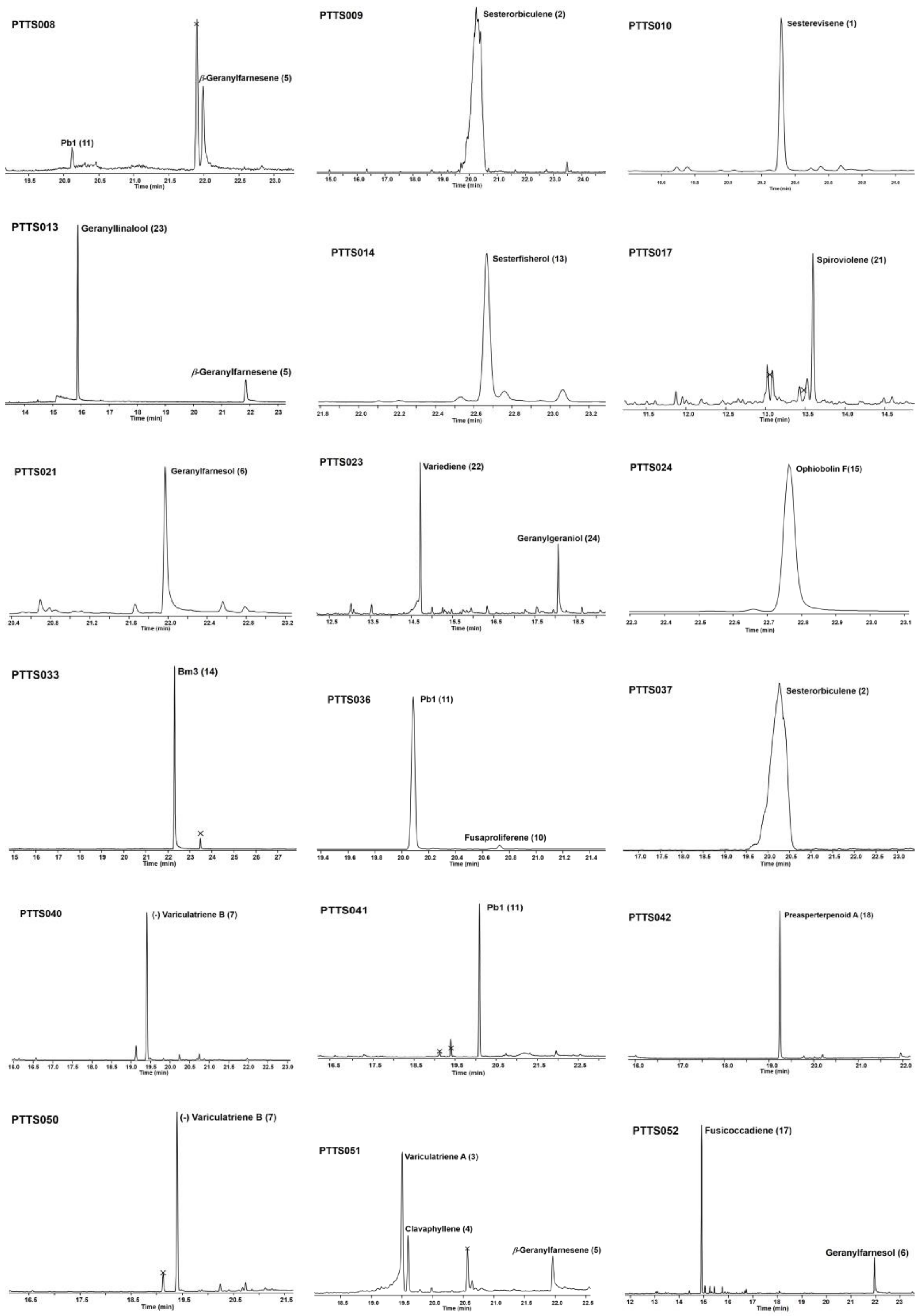
PTTS008
Pb1 (11)
β-Geranylfarnesene (5)
PTTS009
Sesterorbiculene (2)
PTTS010
Sesterevisene (1)
PTTS013
Geranyllinalool (23)
β-Geranylfarnesene (5)
PTTS014
Sesterfisherol (13)
PTTS017
Spiroviolene (21)
PTTS021
Geranylfarnesol (6)
PTTS023
Variediene (22)
Geranylgeraniol (24)
PTTS024
Ophiobolin F(15)
PTTS033
Bm3 (14)
PTTS036
Pb1 (11)
Fusaproliferene (10)
PTTS037
Sesterorbiculene (2)
PTTS040
(-) Variculatriene B (7)
PTTS041
Pb1 (11)
PTTS042
Preasperterpenoid A (18)
PTTS050
(-) Variculatriene B (7)
PTTS051
Variculatriene A (3)
Clavaphyllene (4)
β-Geranylfarnesene (5)
PTTS052
Fusicoccadiene (17)
Geranylfarnesol (6)
Time (min)

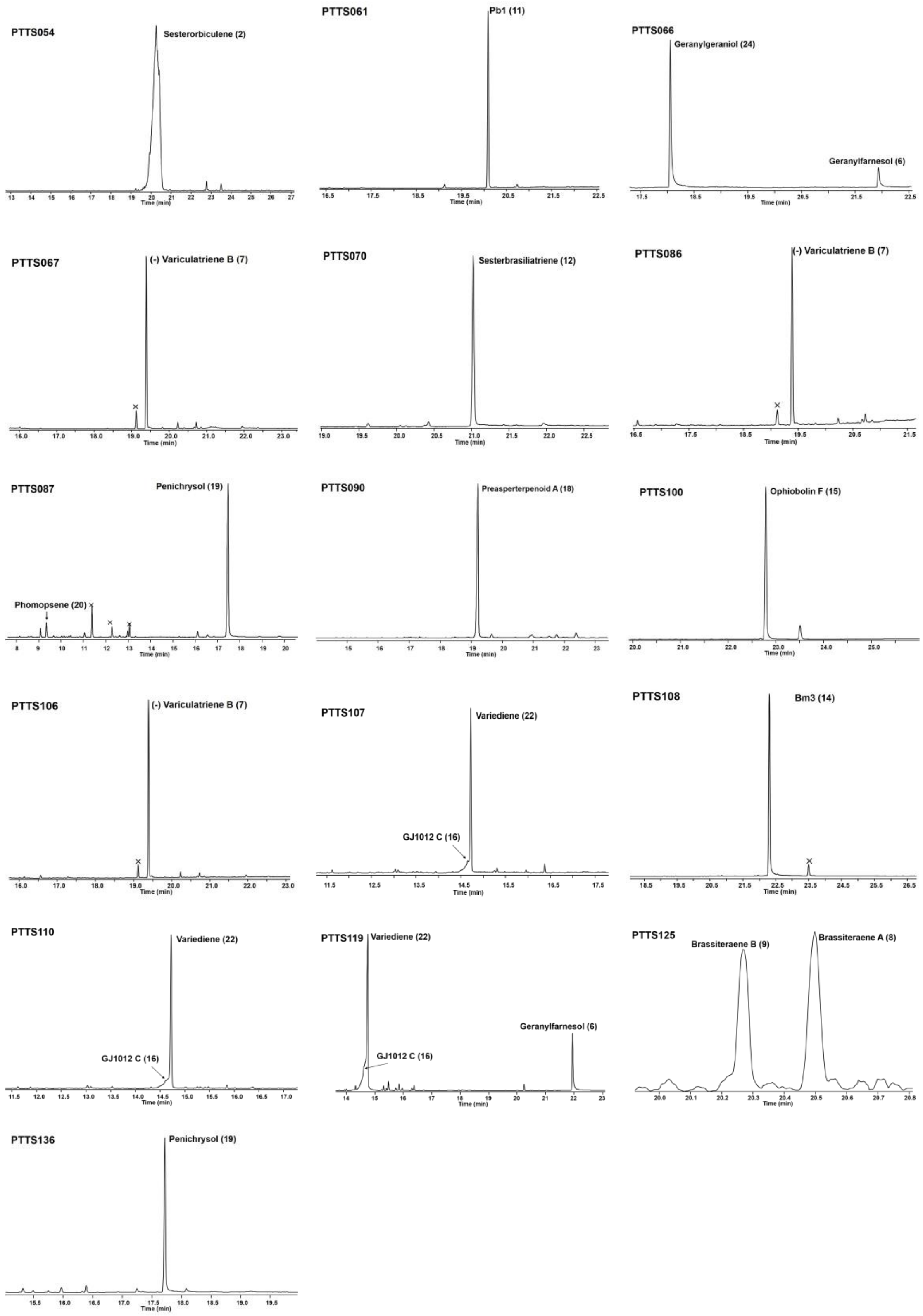

Fig. S4. GC spectrum of di- and sesterterpenes obtained from *S. cerevisiae* strains with PTTSs.

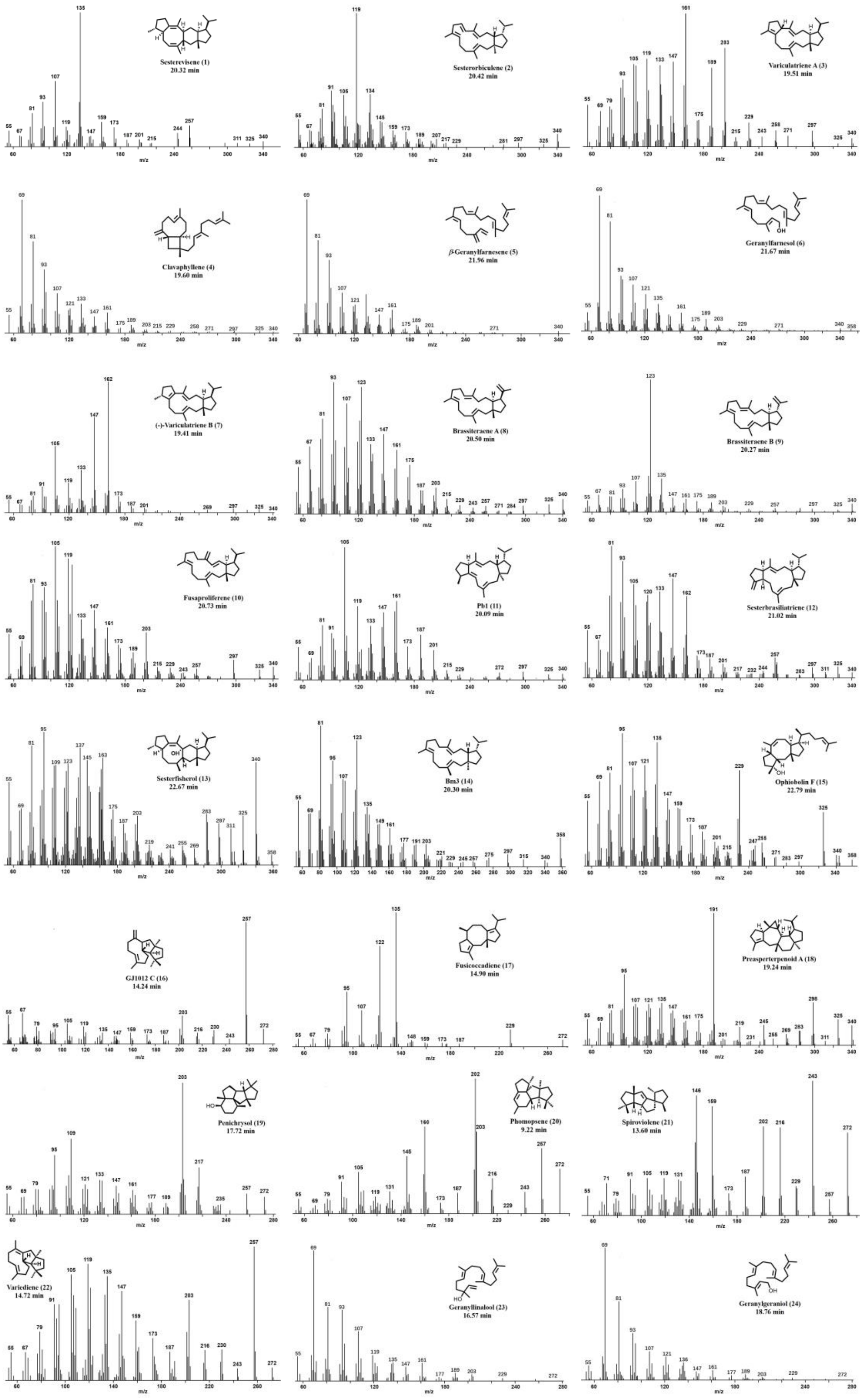

Fig. S5. Mass spectra of di- and sesterterpenes obtained from *S. cerevisiae* strains with PTTSs. Retention time is shown in the parenthesis.

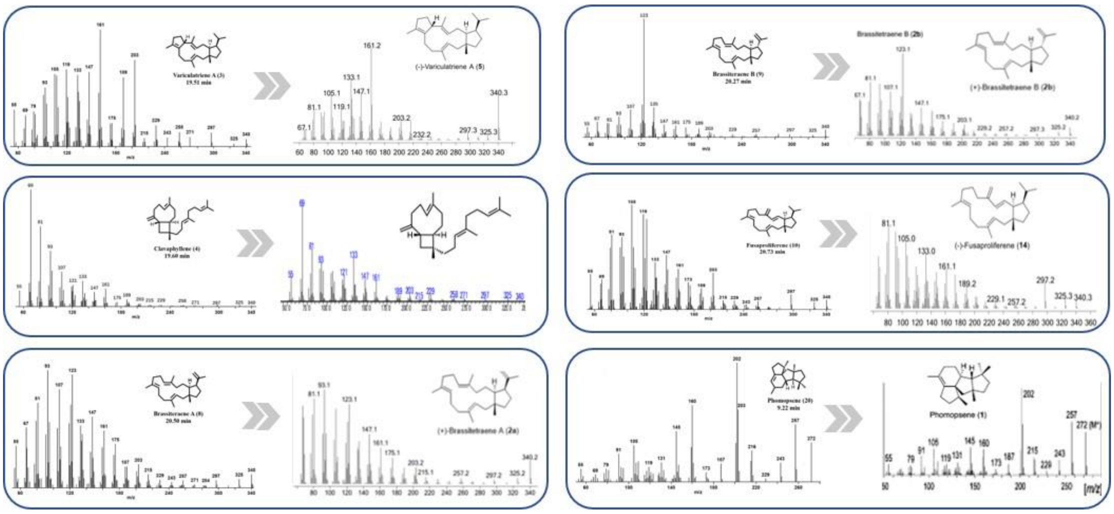

Fig. S6. Mass spectra of six compounds compare with those of known terpenes in reported literature.

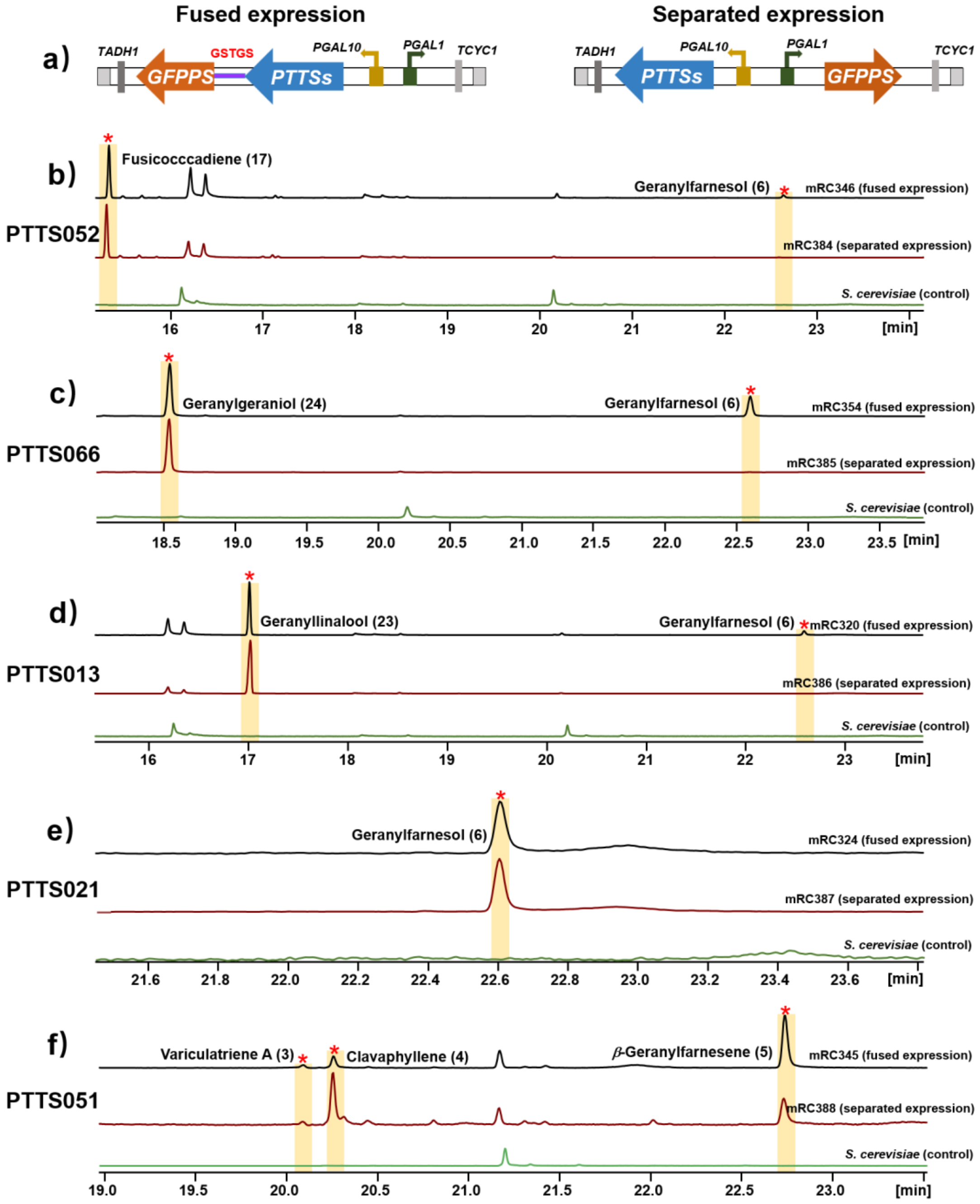

Fig. S7. Heterologous expression of PTTSs in *S. cerevisiae* by fused or separated expression with GFPPS. a) scheme for fused and separated expression of PTTCs and GFPPS; b-f) represent the fused and separated expression of PTTS052, PTTS066, PTTS013, PTTS021 and PTTS051 with GFPPS, respectively.

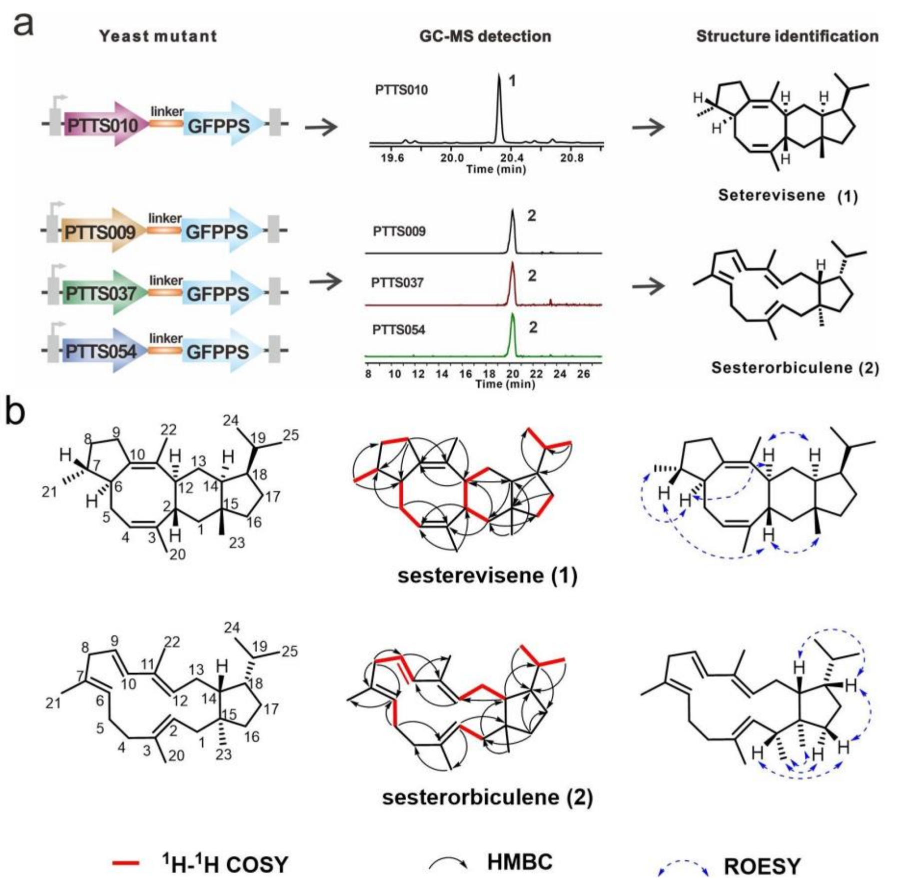

Fig. S8. Characterization of PTTSs in efficient precursor providing *S. cerevisiae* chassis. a) Schematic illustration of the characterization for PTTS010, PTTS037, PTTS009, and PTTS054. b) The key HMBC, ^{1}H-^{1}H COSY and NOESY correlations of Sesterevisene (**1)** and Sesterorbiculene (**2)**.

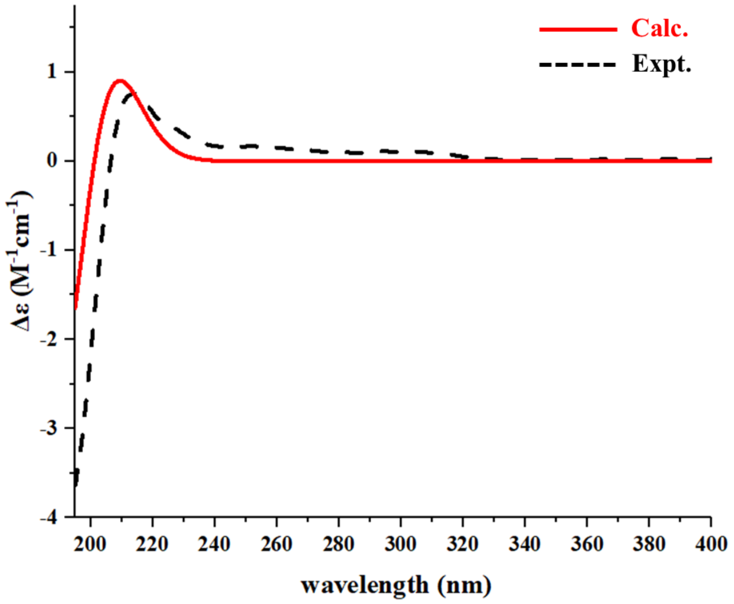

Fig. S9 Calculated and experimental ECD spectrum of **1**.

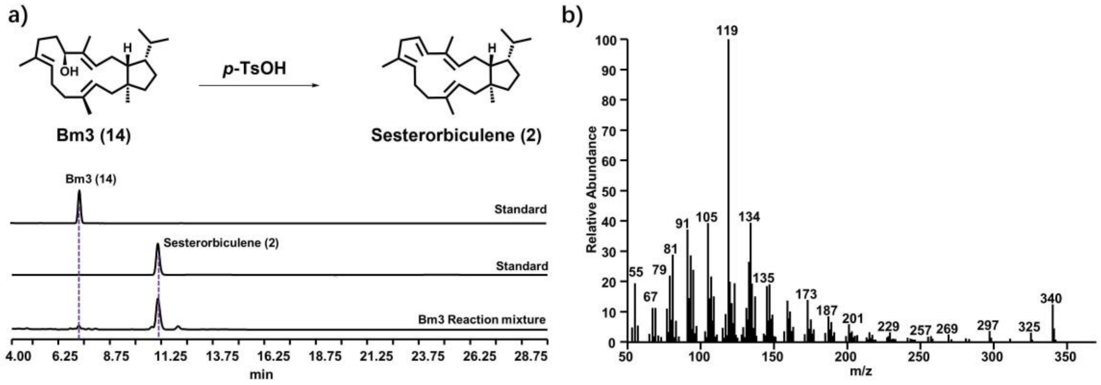

Fig. S10 Results of Bm3 elimination reaction. a) HPLC profile of Bm3 elimination reaction. b) MS spectrum of sesterterpene obtained from reaction mixture. These results showed that Bm3 can be eliminated in the presence of *p*-TsOH to generate sesterorbiculene (**2**). These findings demonstrated that the conformation of the five-membered ring of sesterorbiculene (**2**) is consistent with that of Bm3.

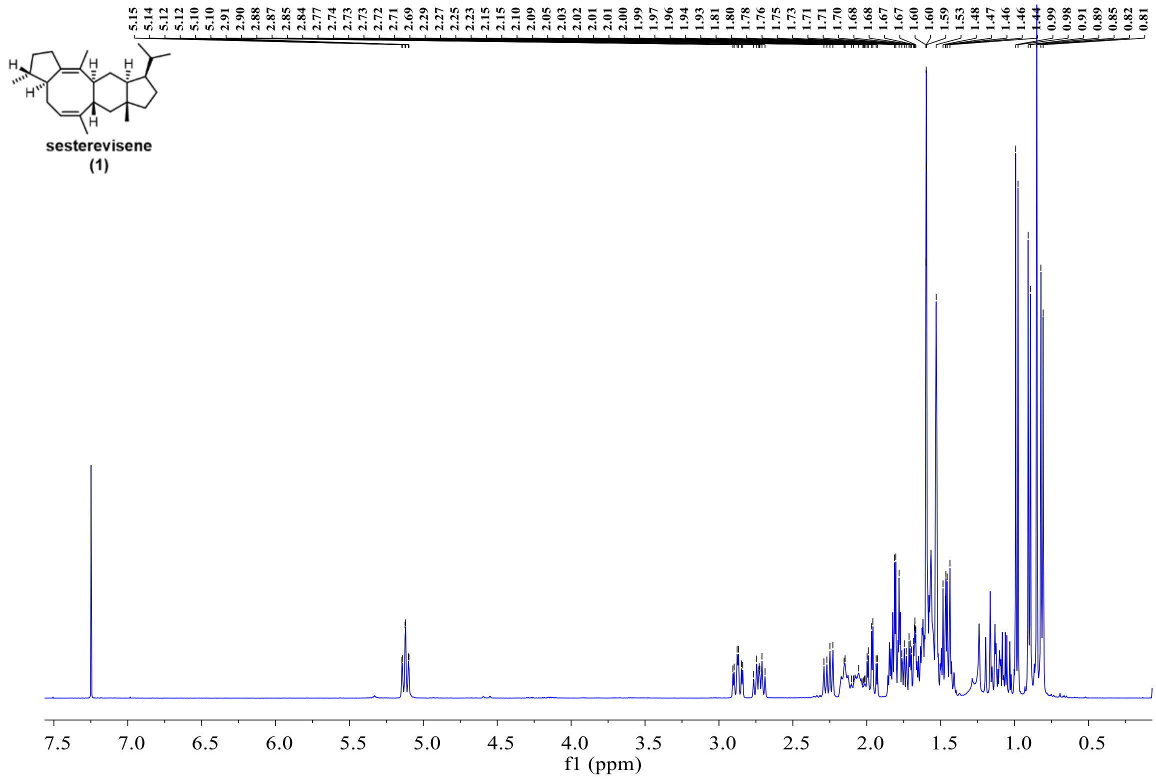

Fig. S11. ^{1}H NMR spectrum of compound **1** ($CDCl_3$, 500 MHz).

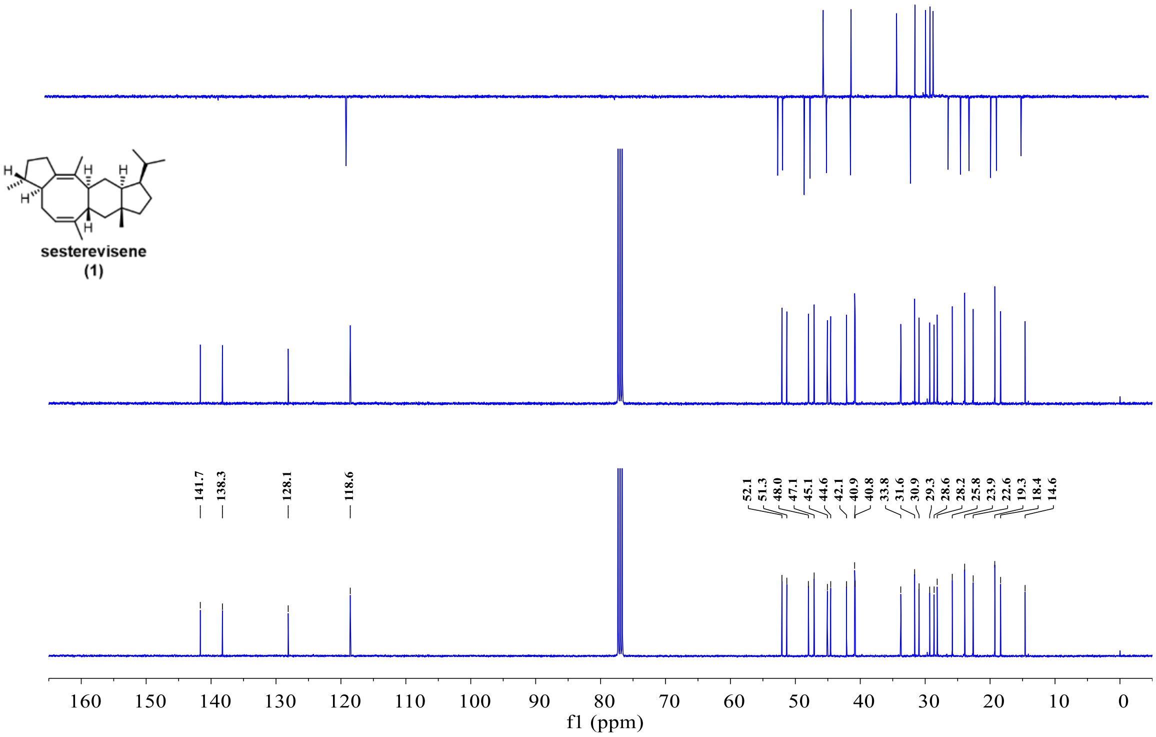

Fig. S12. ^{13}C NMR spectrum of compound **1** ($CDCl_3$, 125 MHz).

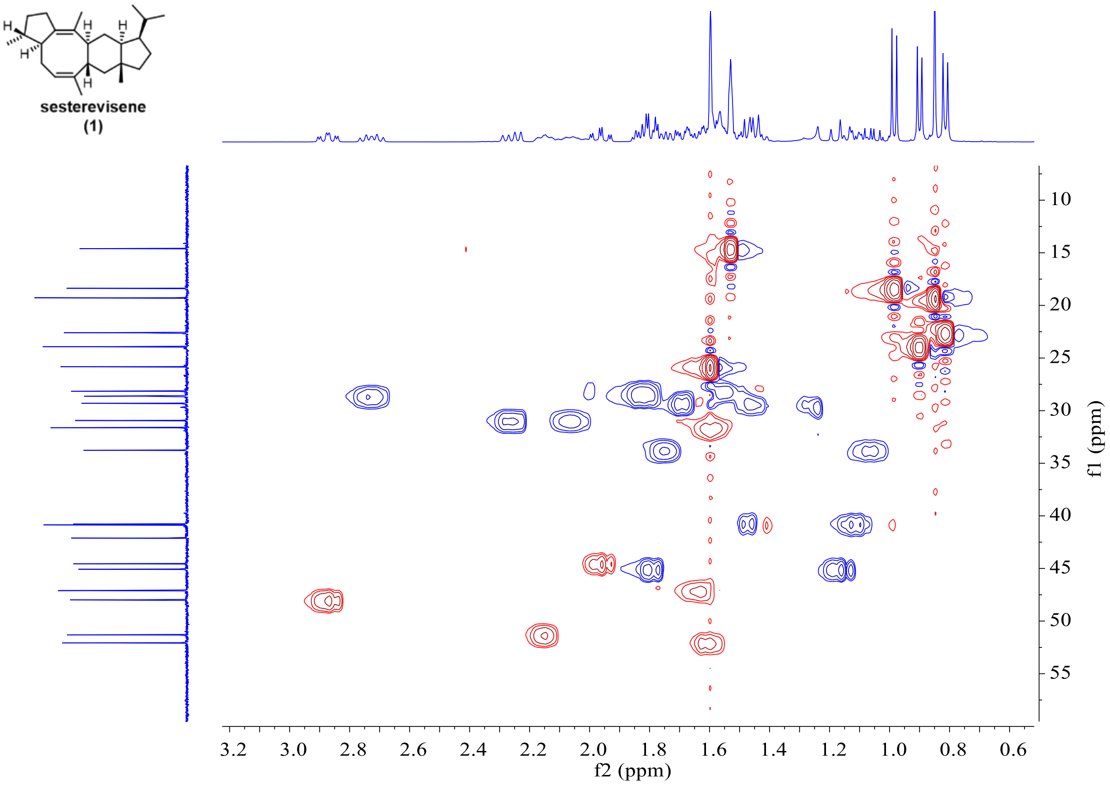

Fig. S13. HSQC spectrum of compound **1** in $CDCl_3$.

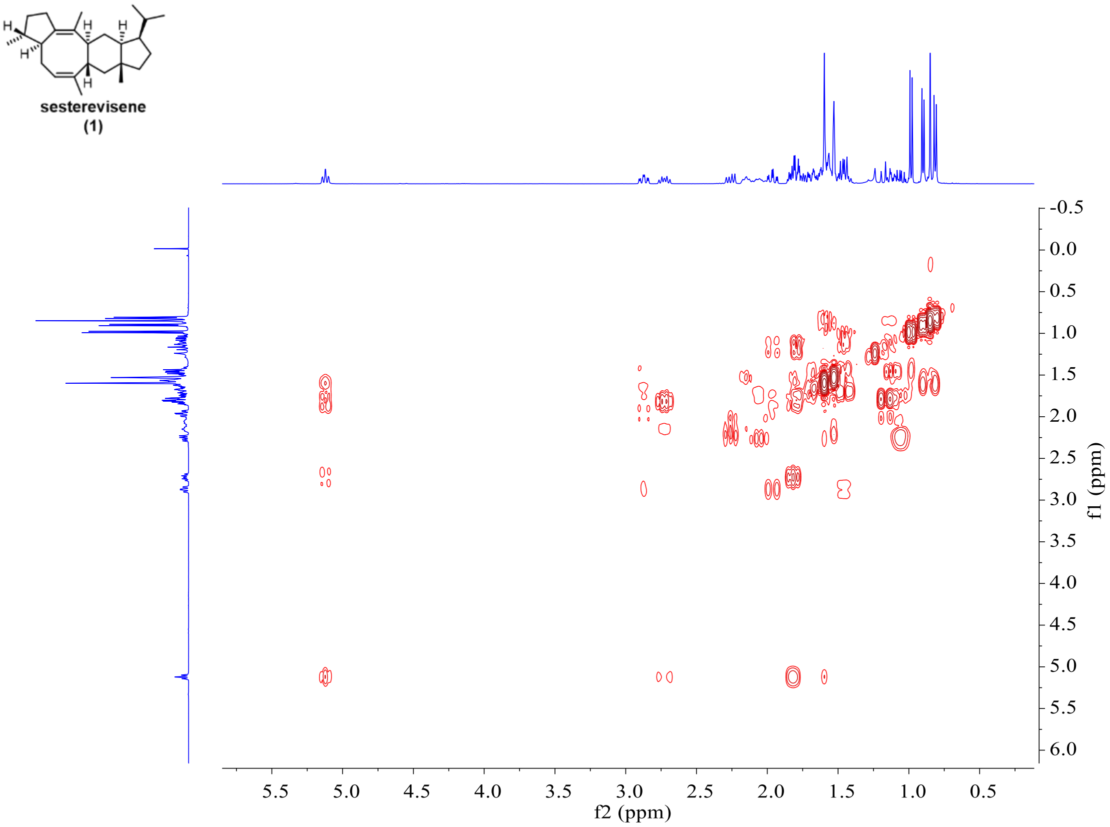

Fig. S14. ^{1}H-^{1}H COSY spectrum of compound **1** in $CDCl_3$.

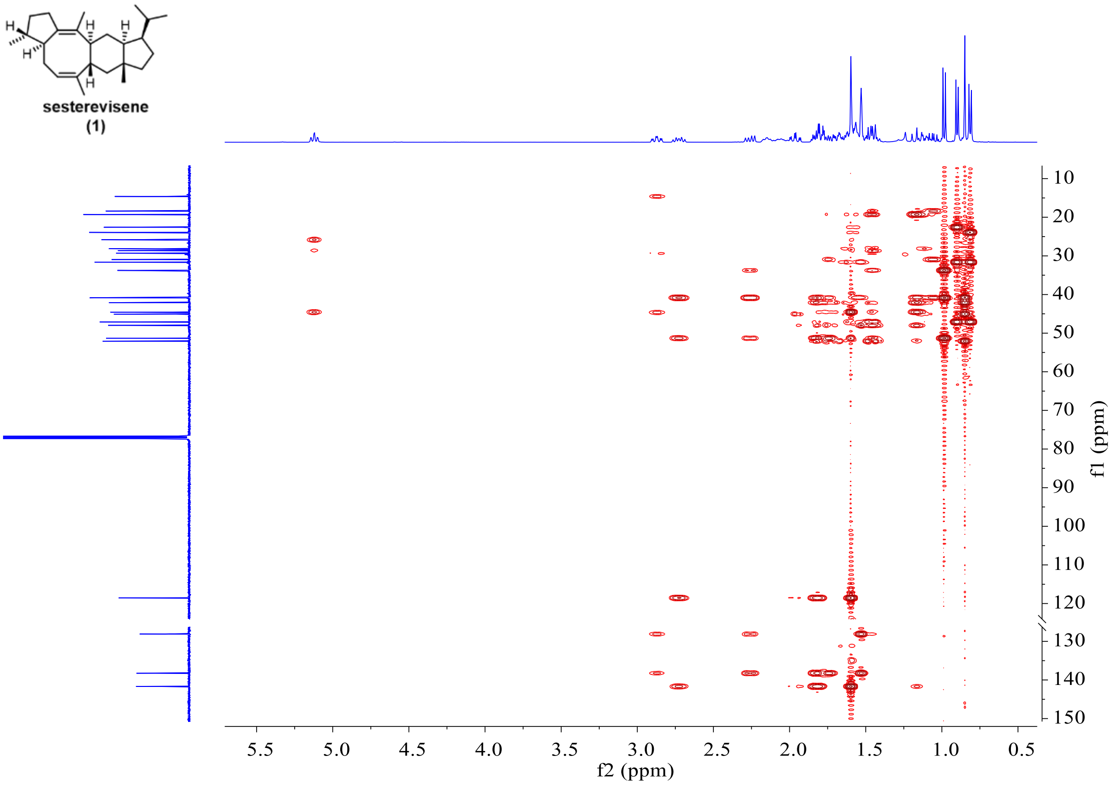

Fig. S15. HMBC spectrum of compound **1** in $CDCl_3$.

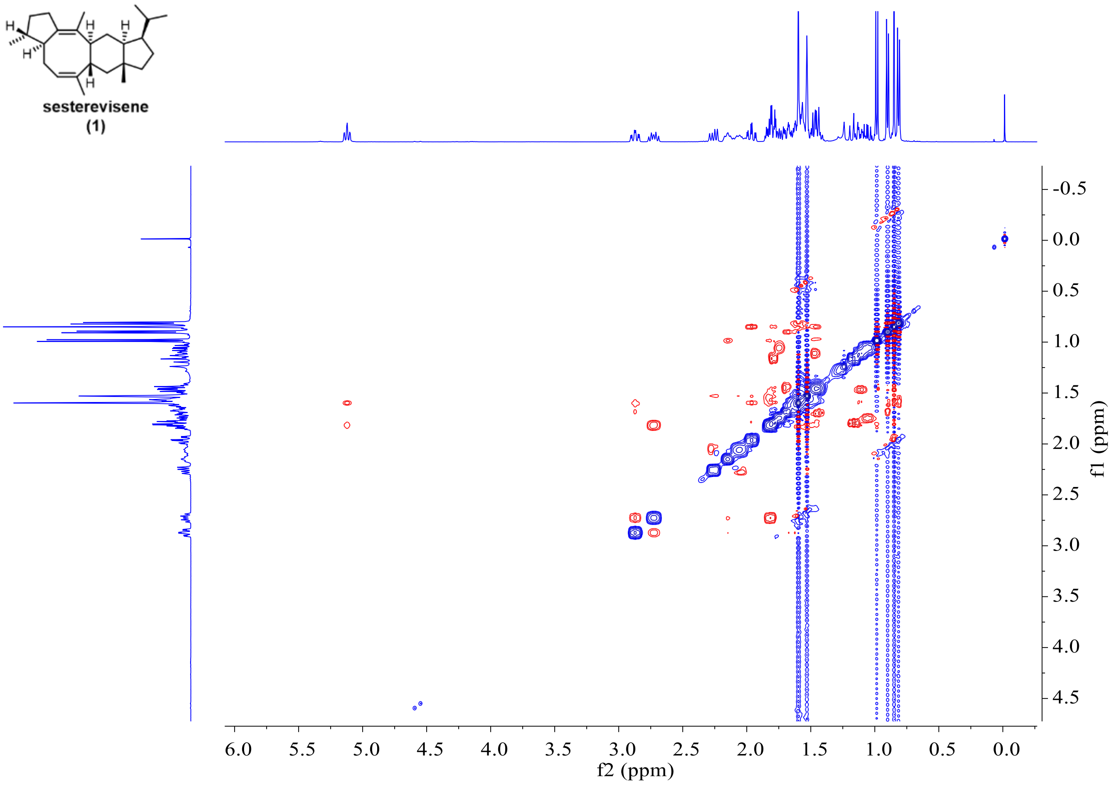

Fig. S16. NOESY spectrum of compound **1** in $CDCl_3$.

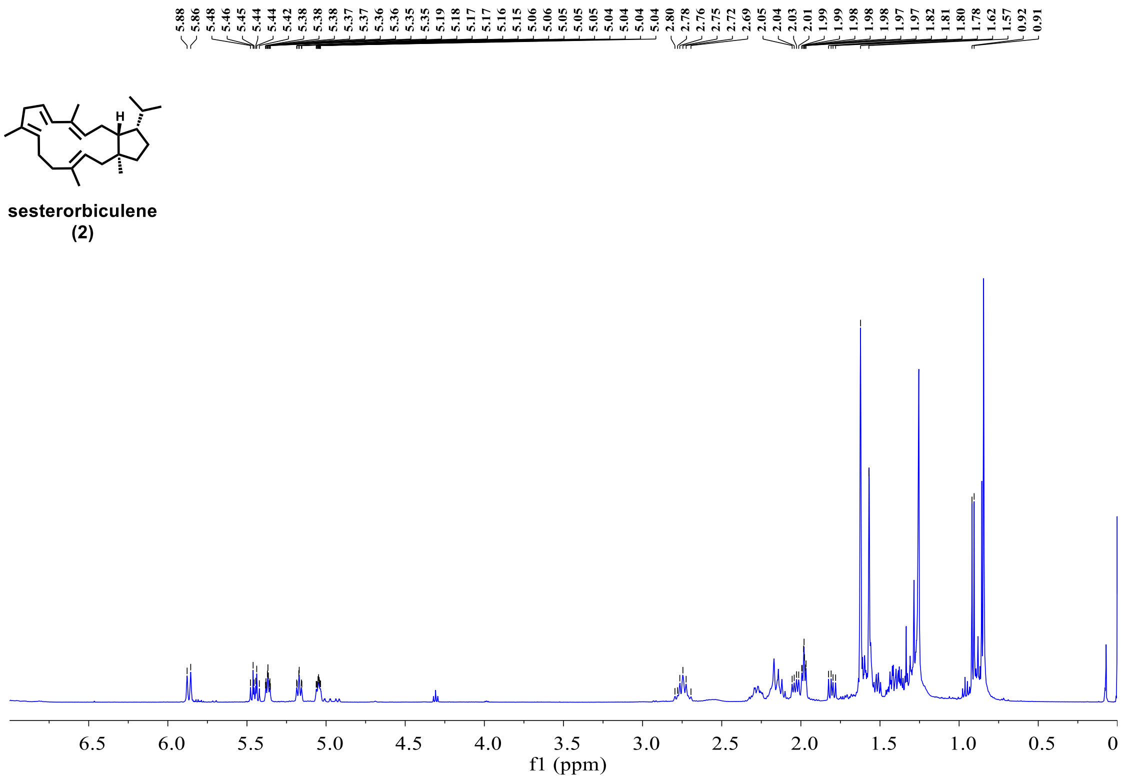

Fig. S17. ^{1}H NMR spectrum of compound **2** ($CDCl_3$, 500 MHz).

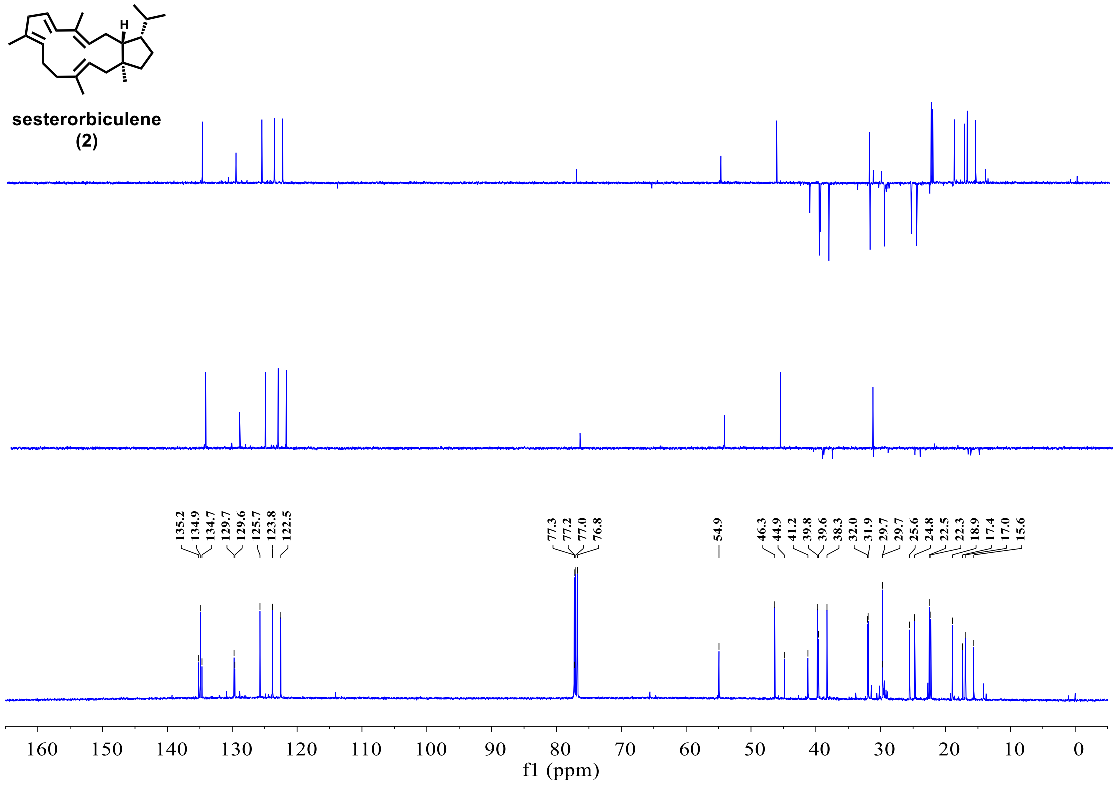

Fig. S18. ^{13}C NMR spectrum of compound **2** ($CDCl_3$, 125 MHz).

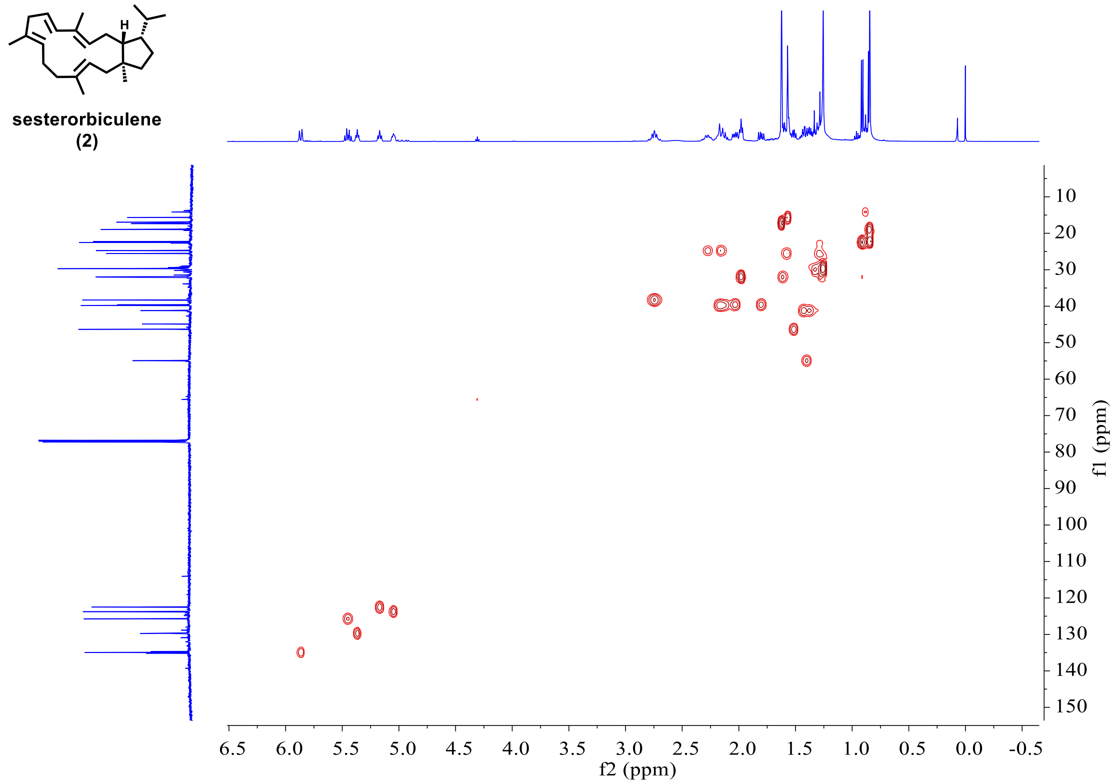

Fig. S19. HSQC spectrum of compound **2** in $CDCl_3$.

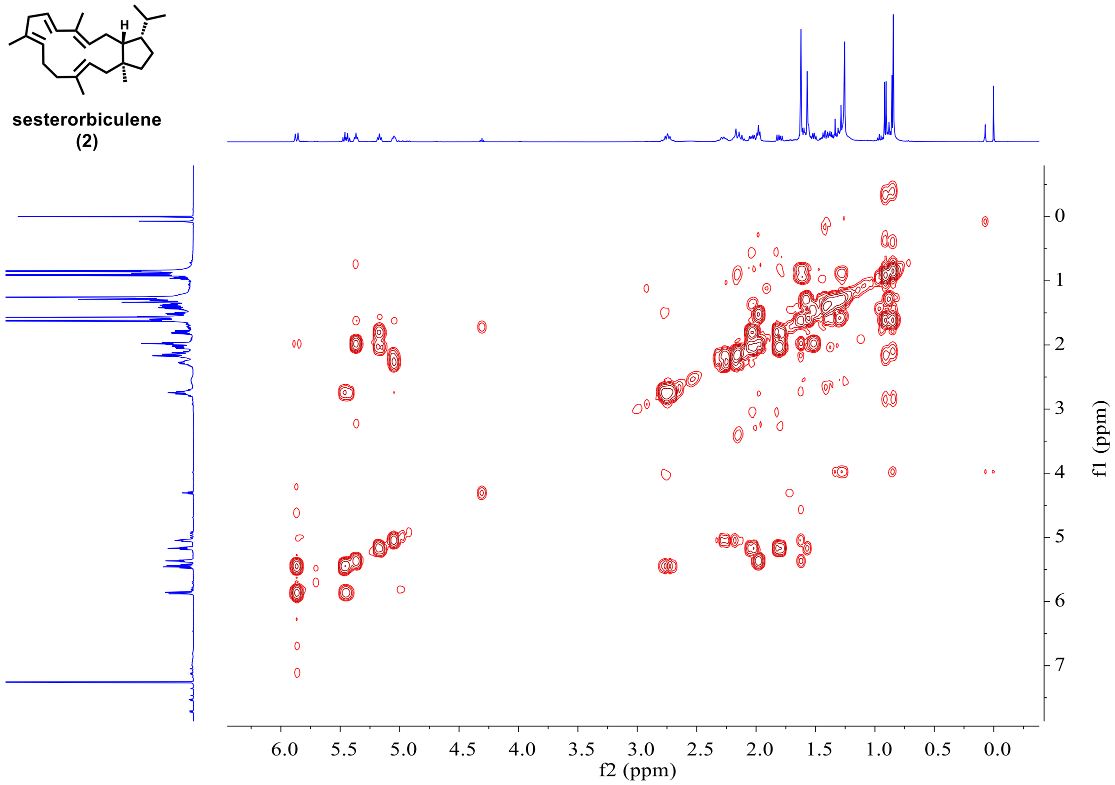

Fig. S20. ^1H-^1H COSY spectrum of compound **2** in $CDCl_3$.

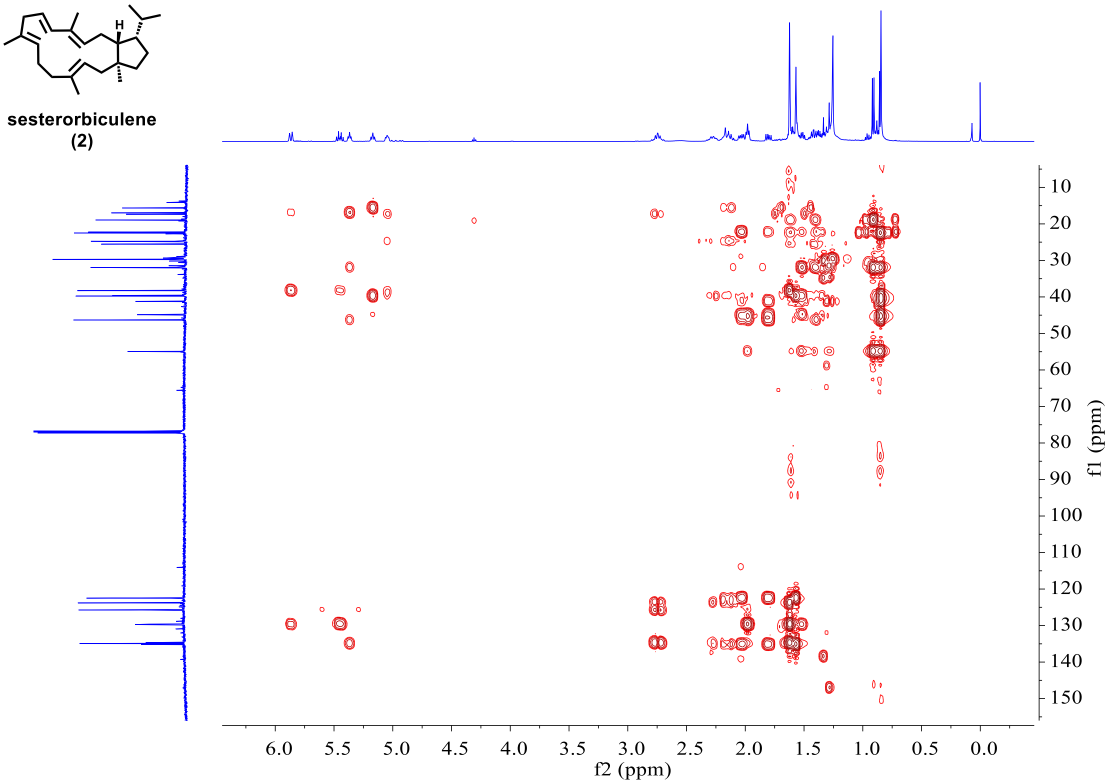

Fig. S21. HMBC spectrum of compound **2** in $CDCl_3$.

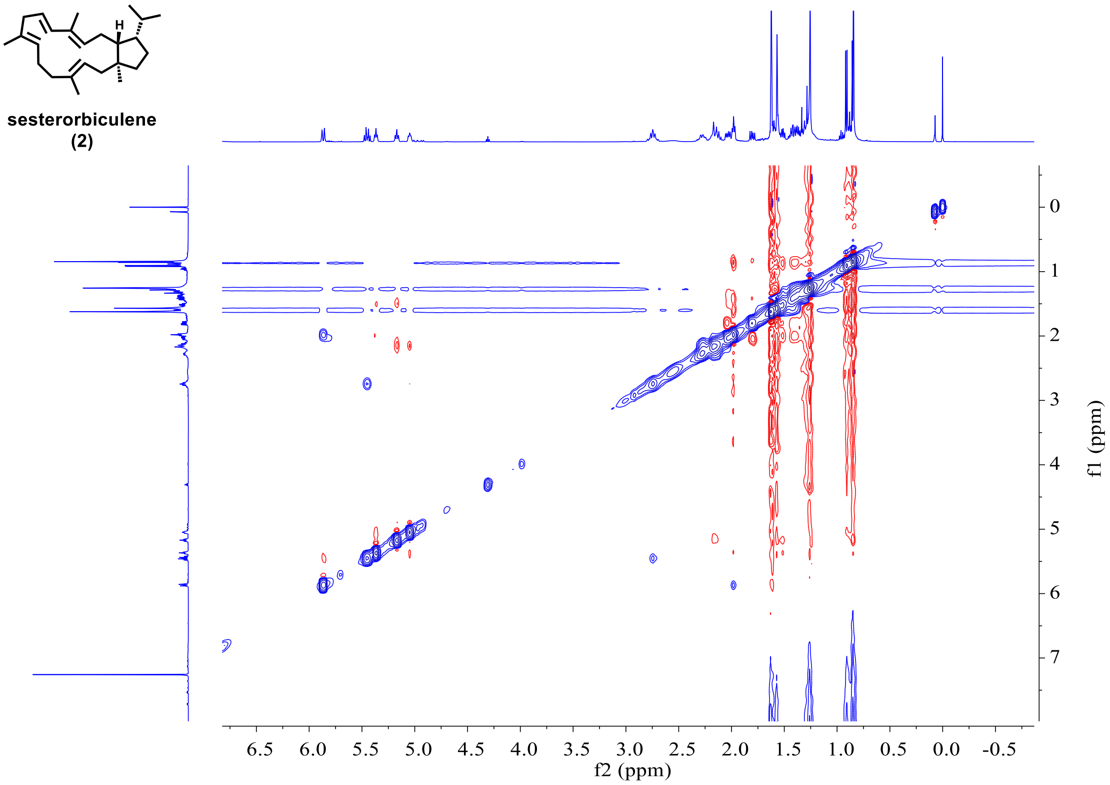

Fig. S22. NOESY spectrum of compound **2** in $CDCl_3$.

Single Mass Analysis (displaying only valid results)
Tolerance = 10.0 PPM / DBE: min = -1.5, max = 50.0
Selected filters: None

Monoisotopic Mass, Odd and Even Electron Ions
5 formula(e) evaluated with 1 results within limits (up to 50 closest results for each mass)
Elements Used:
C: 0-200 H: 0-400

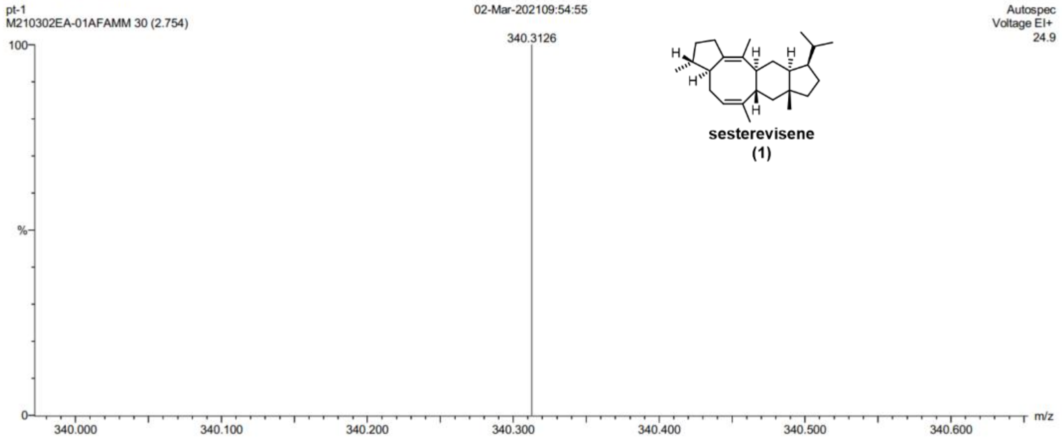

	Mass	Calc. Mass	mDa	PPM	DBE	i-FIT	Formula
Minimum:					-1.5		
Maximum:			500.0	10.0	50.0		
	340.3126	340.3130	-0.4	-1.2	6.0	5546031.5	C25 H40

Fig. S23. HR-EI-MS spectrum of compound **1** in $CDCl_3$.

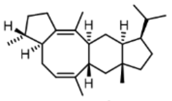

sesterevisene
(1)

Rudolph Research Analytical

This sample was measured on an Autopol VI, Serial #91058
Manufactured by Rudolph Research Analytical, Hackettstown, NJ, USA.

Measurement Date : Thursday, 06-MAY-2021

Set Temperature : OFF

Time Delay : Disabled

Delay between Measurement : Disabled

n	Average	Std.Dev.	% RSD	Maximum	Minimum
5	3.79	0.13	3.43	3.89	3.56

S.No	Sample ID	Time	Result	Scale	OR °Arc	WLG.nm	Lg.mm	Conc.g/100ml	Temp.
1	010	02:08:03 PM	3.89	SR	0.0070	589	100.00	0.180	27.0
2	010	02:08:11 PM	3.83	SR	0.0069	589	100.00	0.180	27.0
3	010	02:08:19 PM	3.83	SR	0.0069	589	100.00	0.180	27.0
4	010	02:08:27 PM	3.83	SR	0.0069	589	100.00	0.180	27.0
5	010	02:08:36 PM	3.56	SR	0.0064	589	100.00	0.180	27.0

Fig. S24. [α] spectrum of compound **1** in $CDCl_3$.

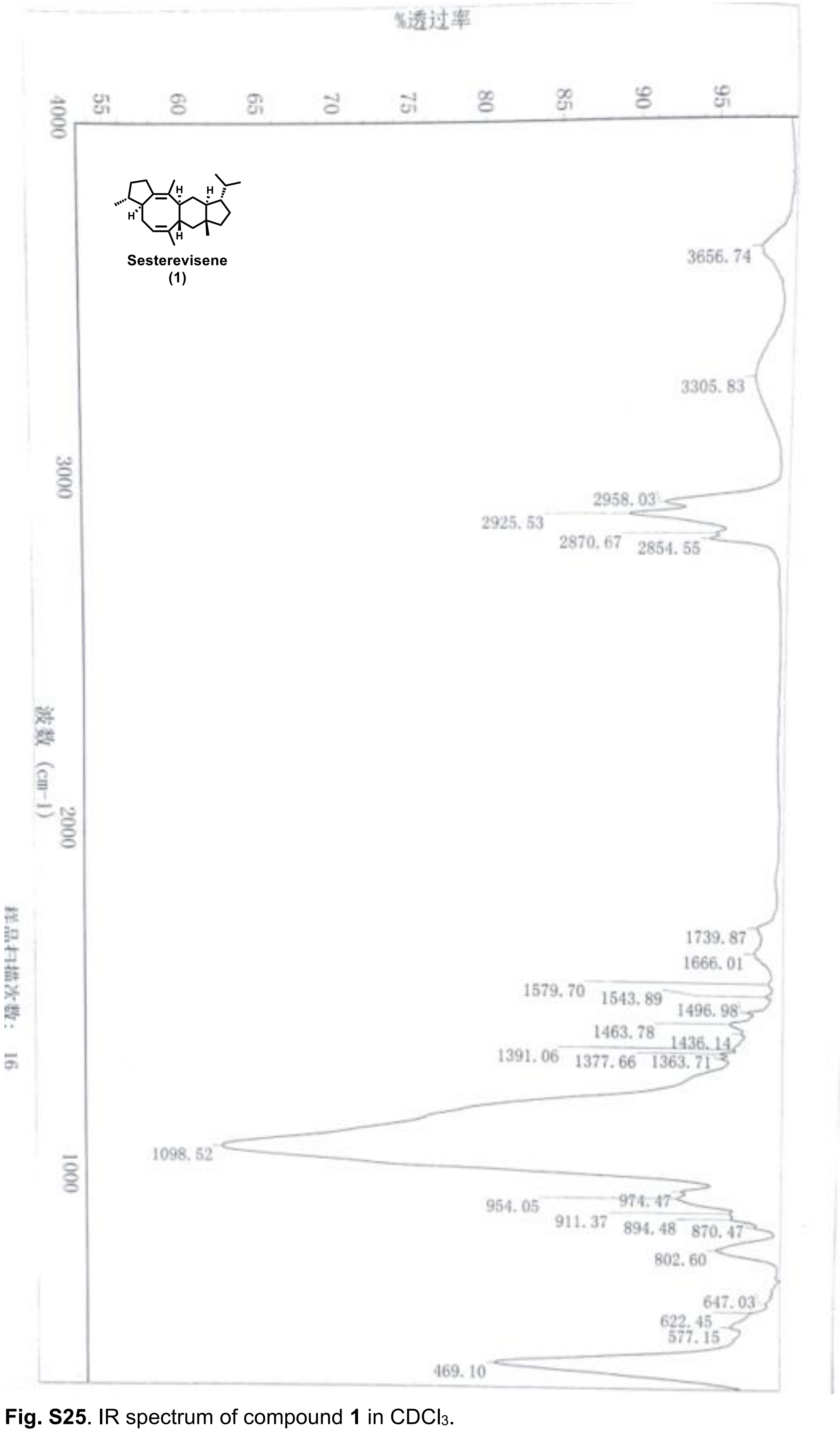

Fig. S25. IR spectrum of compound **1** in $CDCl_3$.

Single Mass Analysis (displaying only valid results)
Tolerance = 10.0 PPM / DBE: min = -1.5, max = 50.0
Selected filters: None

Monoisotopic Mass, Odd and Even Electron Ions
14 formula(e) evaluated with 1 results within limits (up to 50 closest results for each mass)
Elements Used:
C: 0-200 H: 0-400 O: 0-2

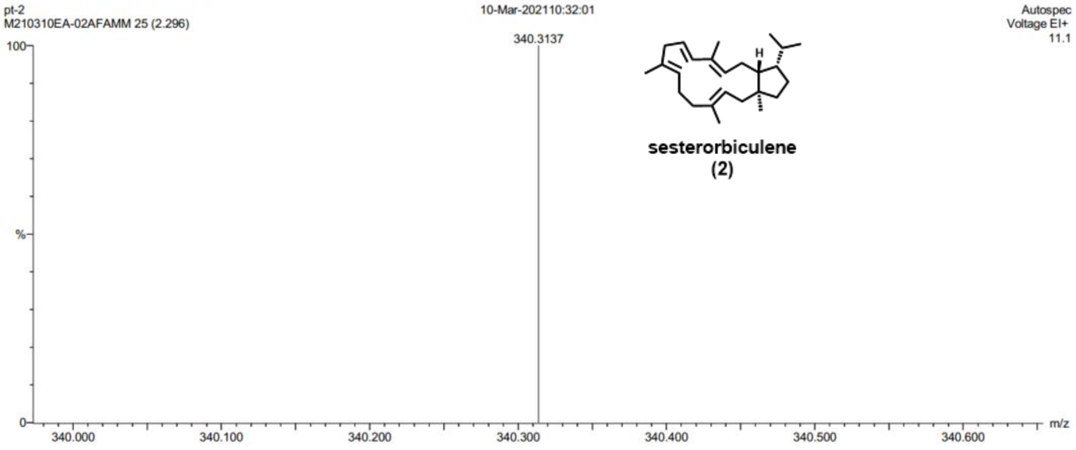

Minimum:				-1.5		
Maximum:		500.0	10.0	50.0		
Mass	Calc. Mass	mDa	PPM	DBE	i-FIT	Formula
340.3137	340.3130	0.7	2.1	6.0	5546026.5	C25 H40

Fig. S26. HR-EI-MS spectrum of compound **2** in $CDCl_3$.

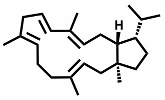

sesterorbiculene
(2)

Rudolph Research Analytical

This sample was measured on an Autopol VI, Serial #91058
Manufactured by Rudolph Research Analytical, Hackettstown, NJ, USA.

Measurement Date : Wednesday, 17-APR-2019

Set Temperature : OFF

Time Delay : Disabled

Delay between Measurement : Disabled

n	Average	Std.Dev.	% RSD	Maximum	Minimum
5	-45.10	1.26	-2.79	-43.90	-47.10

S.No	Sample ID	Time	Result	Scale	OR °Arc	WLG:nm	Lg.mm	Conc.g/100ml	Temp.
1	037	03:45:41 PM	-47.10	SR	-0.0471	589	100.00	0.100	26.9
2	037	03:45:49 PM	-45.40	SR	-0.0454	589	100.00	0.100	26.9
3	037	03:45:57 PM	-44.90	SR	-0.0449	589	100.00	0.100	26.9
4	037	03:46:05 PM	-44.20	SR	-0.0442	589	100.00	0.100	26.9
5	037	03:46:13 PM	-43.90	SR	-0.0439	589	100.00	0.100	26.9

Fig. S27. [α] spectrum of compound **2** in $CDCl_3$.

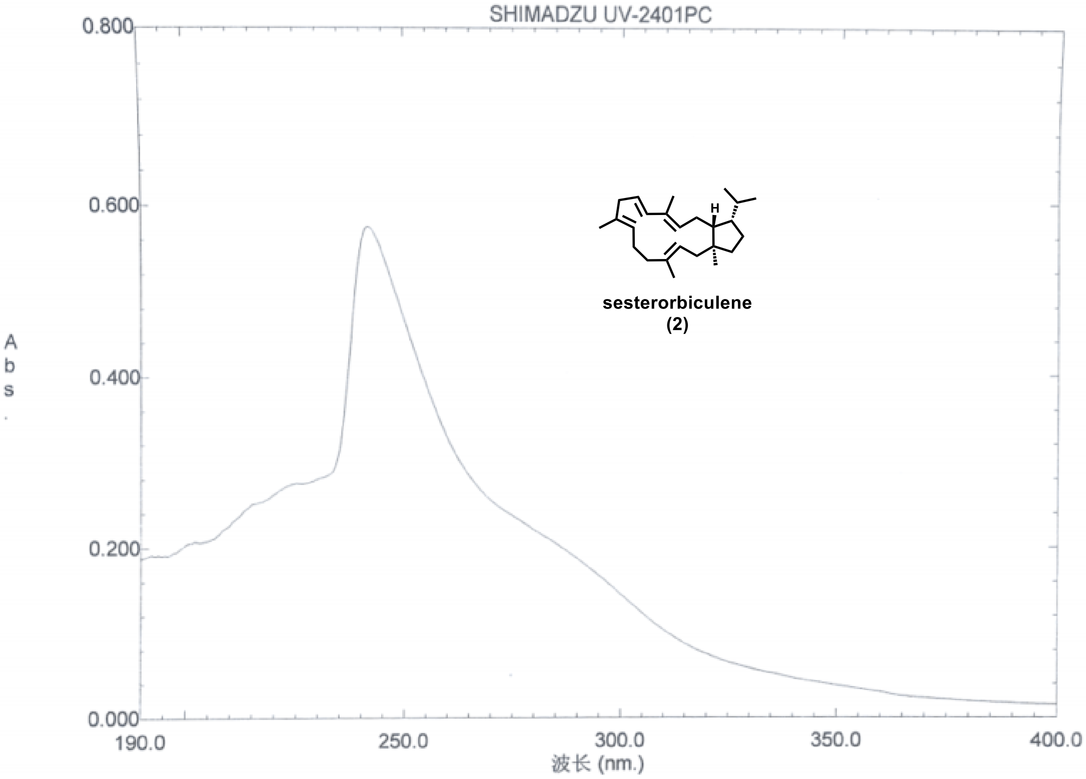

Fig. S28. UV spectrum of compound **2** in $CDCl_3$.

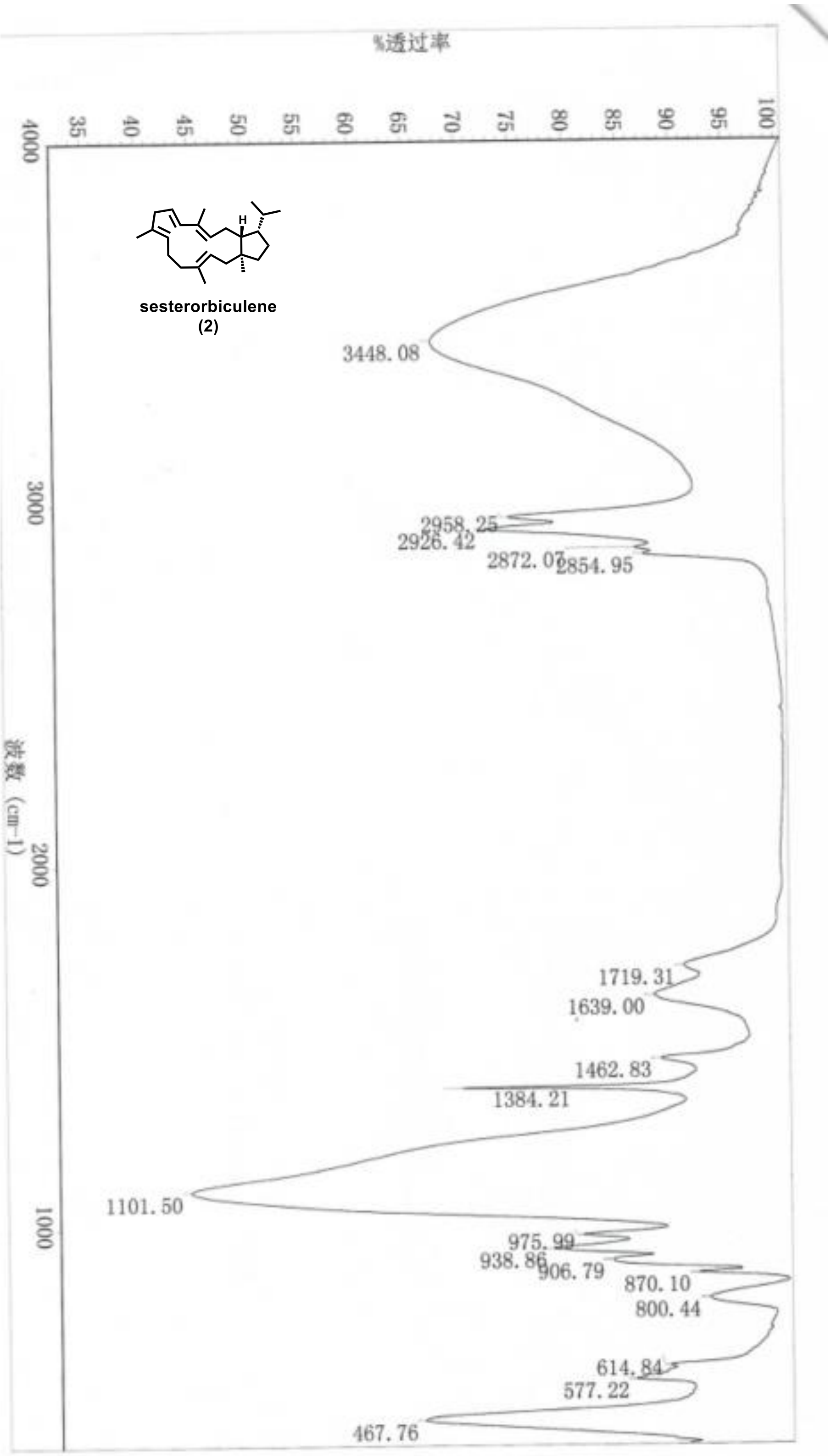

Fig. S29. IR spectrum of compound **2** in $CDCl_3$.

Single Mass Analysis (displaying only valid results)
Tolerance = 10.0 PPM / DBE: min = -1.5, max = 50.0
Selected filters: None

Monoisotopic Mass, Odd and Even Electron Ions
5 formula(e) evaluated with 1 results within limits (up to 50 closest results for each mass)
Elements Used:
C: 0-200 H: 0-400

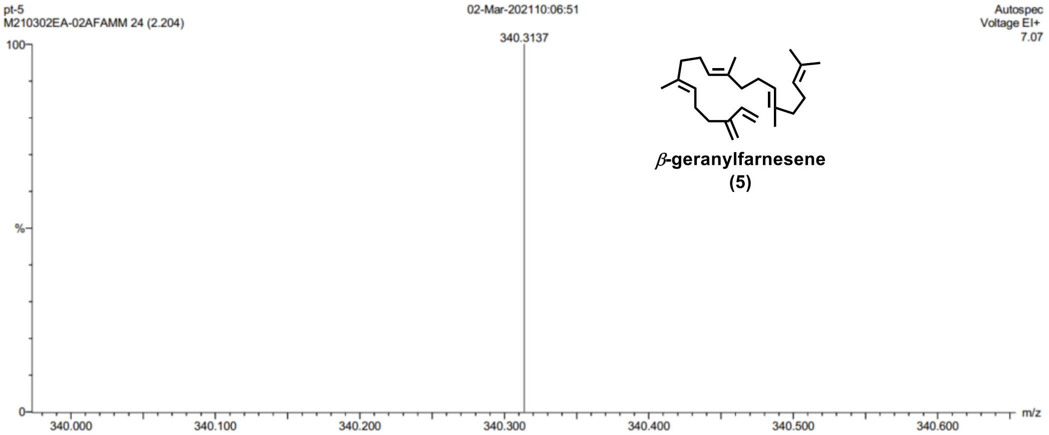

Minimum:				-1.5		
Maximum:		500.0	10.0	50.0		
Mass	Calc. Mass	mDa	PPM	DBE	i-FIT	Formula
340.3137	340.3130	0.7	2.1	6.0	5546025.5	C25 H40

Fig. S30. HR-EI-MS spectrum of compound **5** in $CDCl_3$.

Single Mass Analysis (displaying only valid results)
Tolerance = 10.0 PPM / DBE: min = -1.5, max = 50.0
Selected filters: None

Monoisotopic Mass, Odd and Even Electron Ions
15 formula(e) evaluated with 1 results within limits (up to 50 closest results for each mass)
Elements Used:
C: 0-200 H: 0-400 O: 0-2

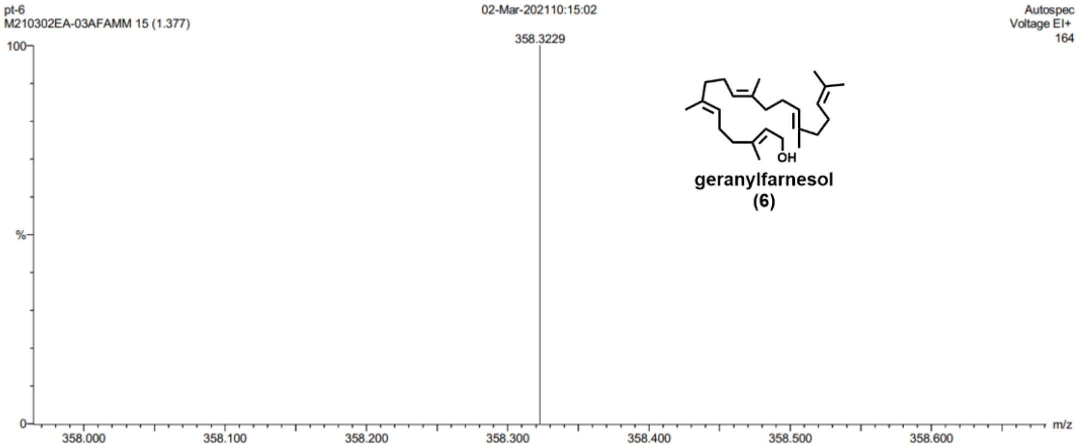

Minimum:				-1.5		
Maximum:		500.0	10.0	50.0		
Mass	Calc. Mass	mDa	PPM	DBE	i-FIT	Formula
358.3229	358.3236	-0.7	-2.0	5.0	5546098.0	C25 H42 O

Fig. S31. HR-EI-MS spectrum of compound **6** in $CDCl_3$.

Single Mass Analysis (displaying only valid results)
Tolerance = 10.0 PPM / DBE: min = -1.5, max = 50.0
Selected filters: None

Monoisotopic Mass, Odd and Even Electron Ions
5 formula(e) evaluated with 1 results within limits (up to 50 closest results for each mass)
Elements Used:
C: 0-200 H: 0-400

7 05-Feb-202110:27:30 Autospec
M210205EA-01AFAMM 44 (4.040) Voltage EI+ 11.4

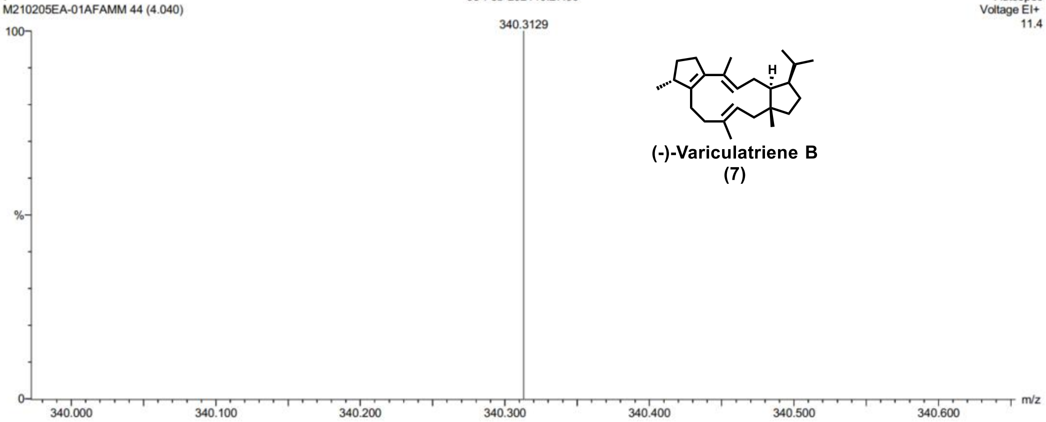

Minimum:				-1.5		
Maximum:		500.0	10.0	50.0		
Mass	Calc. Mass	mDa	PPM	DBE	i-FIT	Formula
340.3129	340.3130	-0.1	-0.3	6.0	5546026.5	C25 H40

Fig. S32. HR-EI-MS spectrum of compound **7** in $CDCl_3$.

Single Mass Analysis (displaying only valid results)
Tolerance = 10.0 PPM / DBE: min = -1.5, max = 50.0
Selected filters: None

Monoisotopic Mass, Odd and Even Electron Ions
14 formula(e) evaluated with 1 results within limits (up to 50 closest results for each mass)
Elements Used:
C: 0-200 H: 0-400 O: 0-2

pt061 10-Mar-202110:47:21 Autospec
M210310EA-04AFAMM 15 (1.377) Voltage EI+ 78.2

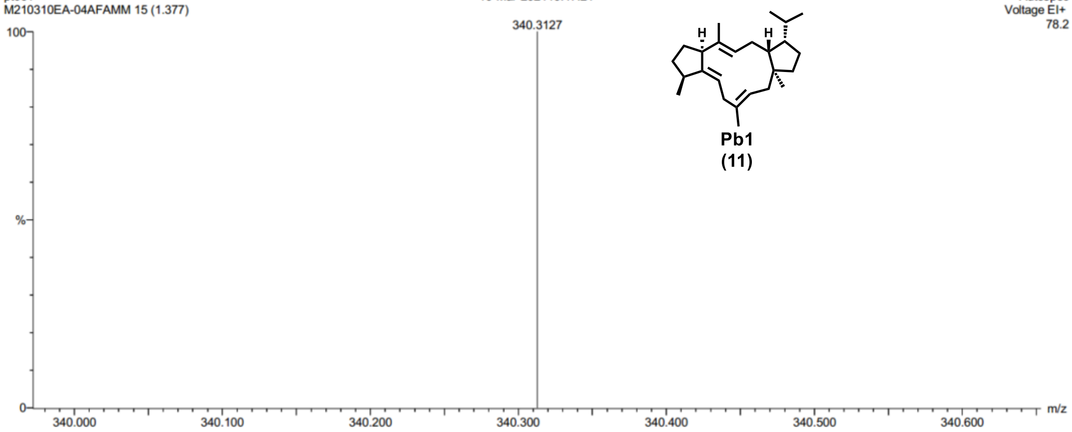

Minimum:				-1.5		
Maximum:		500.0	10.0	50.0		
Mass	Calc. Mass	mDa	PPM	DBE	i-FIT	Formula
340.3127	340.3130	-0.3	-0.9	6.0	5546056.0	C25 H40

Fig. S33. HR-EI-MS spectrum of compound **11** in $CDCl_3$.

Single Mass Analysis (displaying only valid results)
Tolerance = 10.0 PPM / DBE: min = -1.5, max = 50.0
Selected filters: None

Monoisotopic Mass, Odd and Even Electron Ions
14 formula(e) evaluated with 1 results within limits (up to 50 closest results for each mass)
Elements Used:
C: 0-200 H: 0-400 O: 0-2

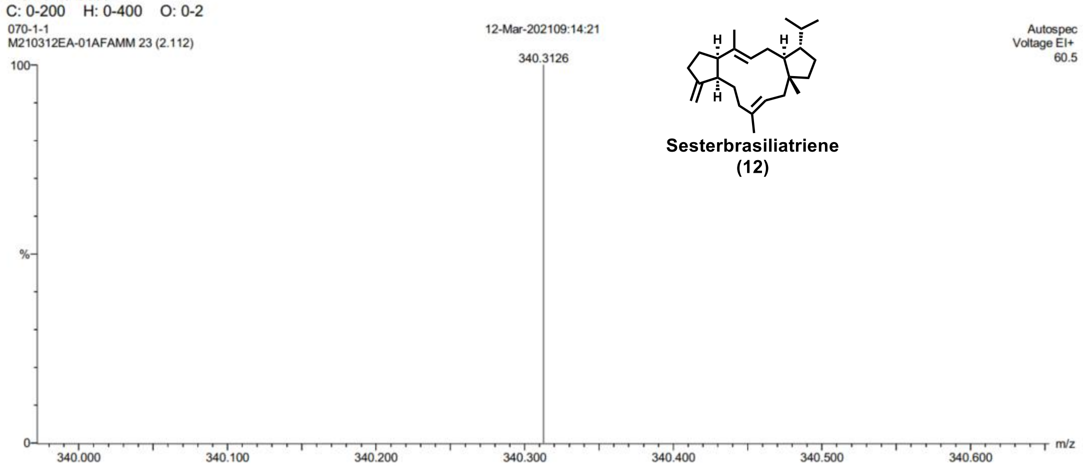

Minimum:				-1.5		
Maximum:		500.0	10.0	50.0		
Mass	Calc. Mass	mDa	PPM	DBE	i-FIT	Formula
340.3126	340.3130	-0.4	-1.2	6.0	5546047.5	C25 H40

Fig. S34. HR-EI-MS spectrum of compound **12** in $CDCl_3$.

Single Mass Analysis (displaying only valid results)
Tolerance = 10.0 PPM / DBE: min = -1.5, max = 50.0
Selected filters: None

Monoisotopic Mass, Odd and Even Electron Ions
15 formula(e) evaluated with 1 results within limits (up to 50 closest results for each mass)
Elements Used:
C: 0-200 H: 0-400 O: 0-2

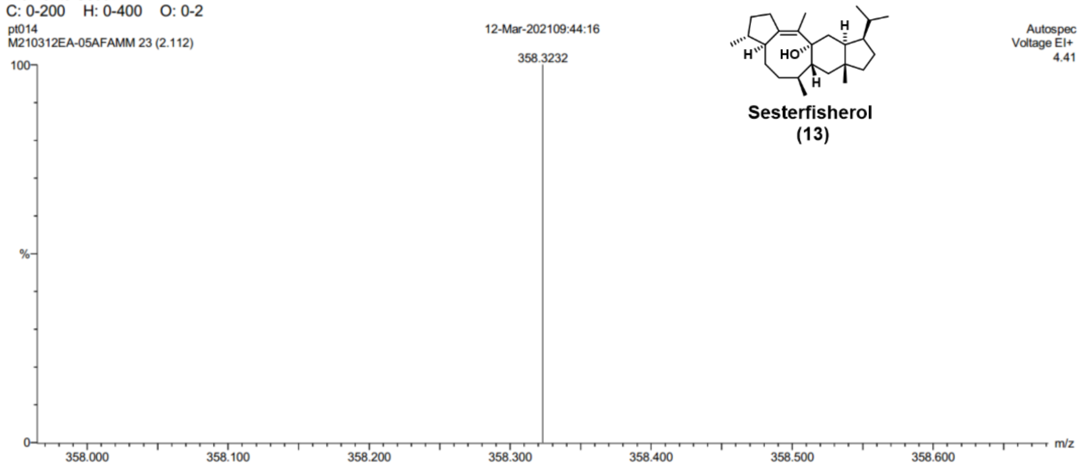

Minimum:				-1.5		
Maximum:		500.0	10.0	50.0		
Mass	Calc. Mass	mDa	PPM	DBE	i-FIT	Formula
358.3232	358.3236	-0.4	-1.1	5.0	5546025.5	C25 H42 O

Fig. S35. HR-EI-MS spectrum of compound **13** in $CDCl_3$.

Single Mass Analysis (displaying only valid results)
Tolerance = 10.0 PPM / DBE: min = -1.5, max = 50.0
Selected filters: None

Monoisotopic Mass, Odd and Even Electron Ions
15 formula(e) evaluated with 1 results within limits (up to 50 closest results for each mass)
Elements Used:
C: 0-200 H: 0-400 O: 0-2

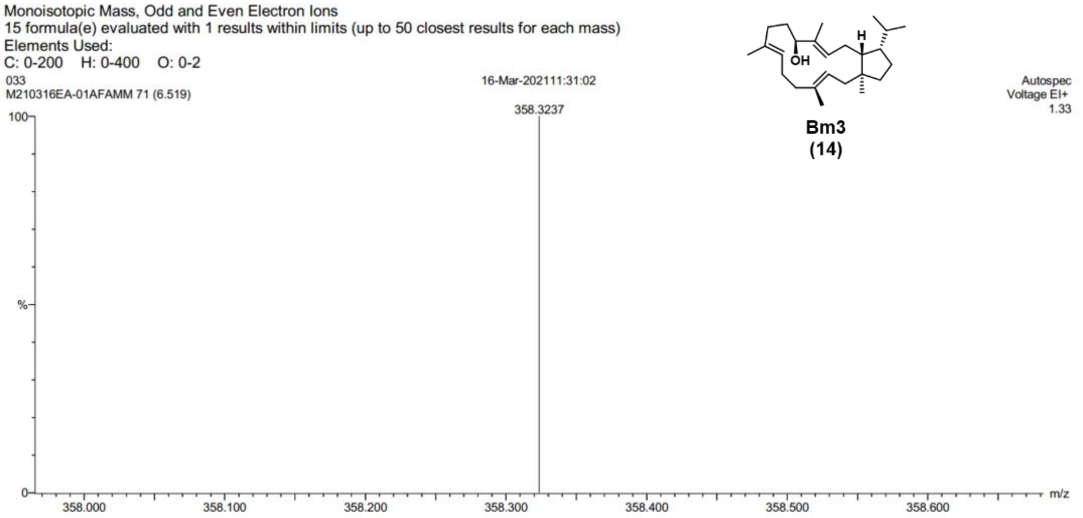

Minimum:				-1.5		
Maximum:		500.0	10.0	50.0		
Mass	Calc. Mass	mDa	PPM	DBE	i-FIT	Formula
358.3237	358.3236	0.1	0.3	5.0	5546025.5	C25 H42 O

Fig. S36. HR-EI-MS spectrum of compound **14** in $CDCl_3$.

Single Mass Analysis (displaying only valid results)
Tolerance = 10.0 PPM / DBE: min = -1.5, max = 50.0
Selected filters: None

Monoisotopic Mass, Odd and Even Electron Ions
15 formula(e) evaluated with 1 results within limits (up to 50 closest results for each mass)
Elements Used:
C: 0-200 H: 0-400 O: 0-2

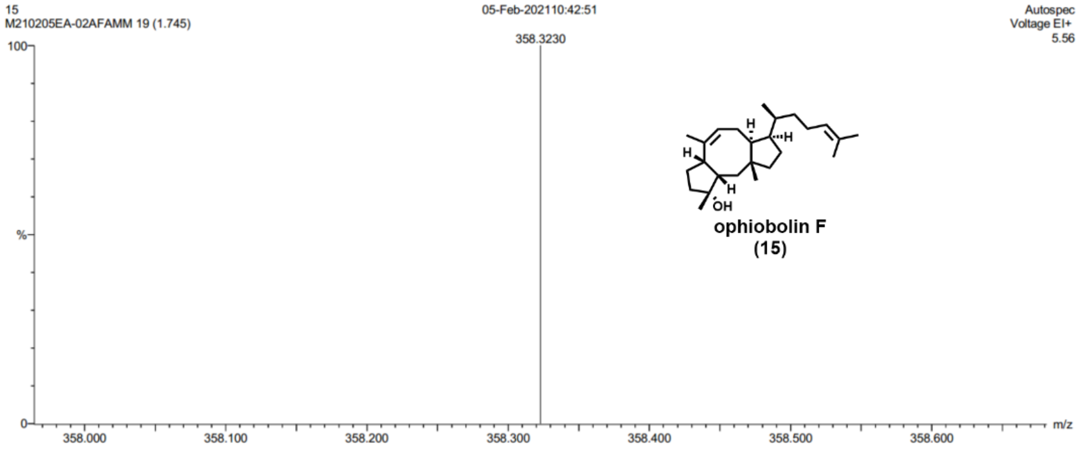

Minimum:				-1.5		
Maximum:		500.0	10.0	50.0		
Mass	Calc. Mass	mDa	PPM	DBE	i-FIT	Formula
358.3230	358.3236	-0.6	-1.7	5.0	5546025.5	C25 H42 O

Fig. S37. HR-EI-MS spectrum of compound **15** in $CDCl_3$.

Single Mass Analysis (displaying only valid results)
Tolerance = 10.0 PPM / DBE: min = -1.5, max = 50.0
Selected filters: None

Monoisotopic Mass, Odd and Even Electron Ions
12 formula(e) evaluated with 1 results within limits (up to 50 closest results for each mass)
Elements Used:
C: 0-200 H: 0-400 O: 0-2

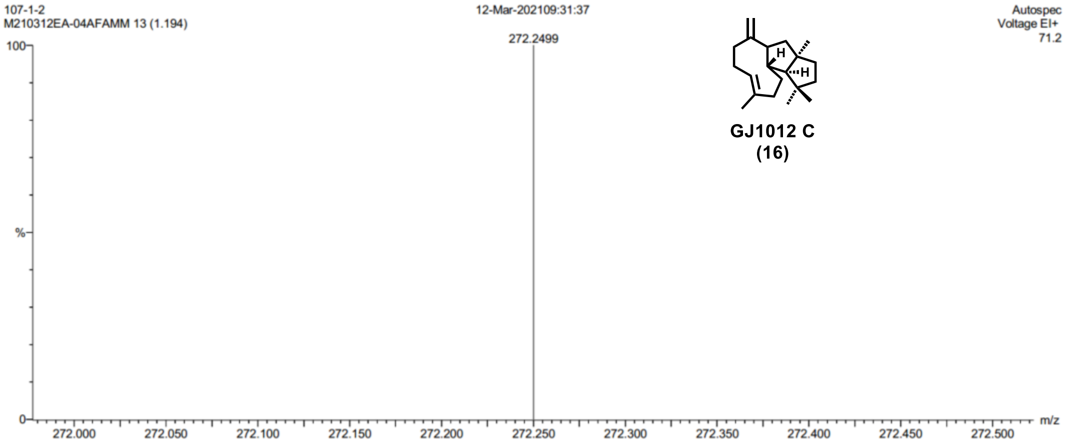

GJ1012 C
(16)

Minimum:				-1.5		
Maximum:		500.0	10.0	50.0		
Mass	Calc. Mass	mDa	PPM	DBE	i-FIT	Formula
272.2499	272.2504	-0.5	-1.8	5.0	5546050.0	C20 H32

Fig. S38. HR-EI-MS spectrum of compound **16** in $CDCl_3$.

Single Mass Analysis (displaying only valid results)
Tolerance = 10.0 PPM / DBE: min = -1.5, max = 50.0
Selected filters: None

Monoisotopic Mass, Odd and Even Electron Ions
12 formula(e) evaluated with 1 results within limits (up to 50 closest results for each mass)
Elements Used:
C: 0-200 H: 0-400 O: 0-2

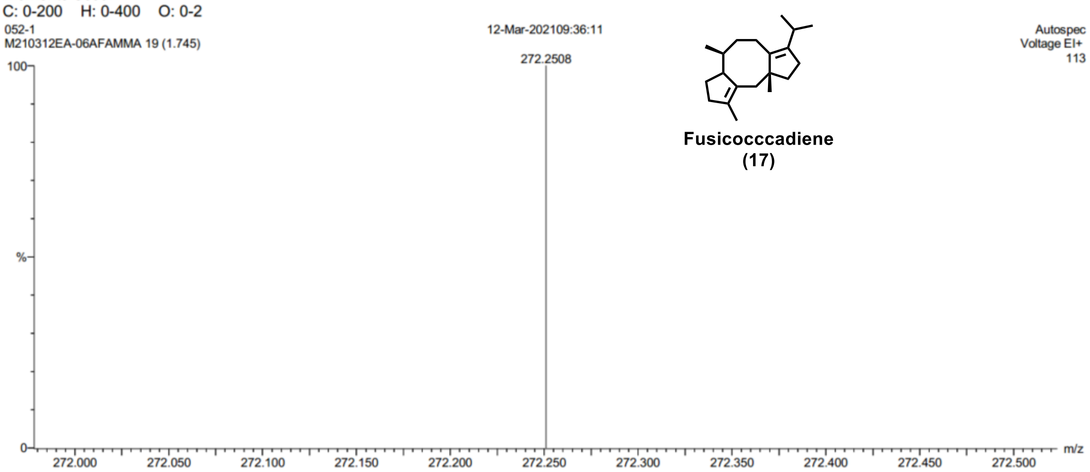

Fusicocccadiene
(17)

Minimum:				-1.5		
Maximum:		500.0	10.0	50.0		
Mass	Calc. Mass	mDa	PPM	DBE	i-FIT	Formula
272.2508	272.2504	0.4	1.5	5.0	5546070.0	C20 H32

Fig. S39. HR-EI-MS spectrum of compound **17** in $CDCl_3$.

Single Mass Analysis (displaying only valid results)
Tolerance = 10.0 PPM / DBE: min = -1.5, max = 50.0
Selected filters: None

Monoisotopic Mass, Odd and Even Electron Ions
5 formula(e) evaluated with 1 results within limits (up to 50 closest results for each mass)
Elements Used:
C: 0-200 H: 0-400

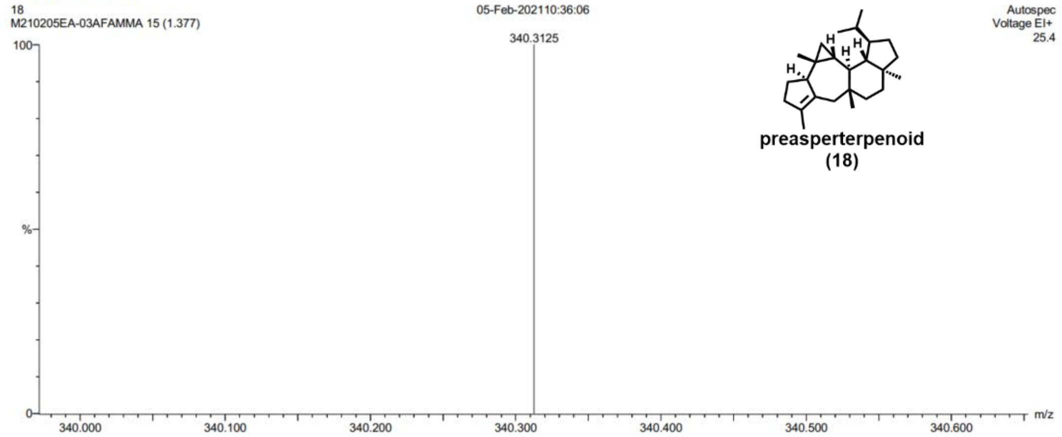

Minimum:				-1.5		
Maximum:		500.0	10.0	50.0		
Mass	Calc. Mass	mDa	PPM	DBE	i-FIT	Formula
340.3125	340.3130	-0.5	-1.5	6.0	5546031.5	C25 H40

Fig. S40. HR-EI-MS spectrum of compound **18** in $CDCl_3$

Single Mass Analysis (displaying only valid results)
Tolerance = 10.0 PPM / DBE: min = -1.5, max = 50.0
Selected filters: None

Monoisotopic Mass, Odd and Even Electron Ions
13 formula(e) evaluated with 1 results within limits (up to 50 closest results for each mass)
Elements Used:
C: 0-200 H: 0-400 O: 0-2

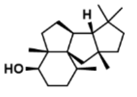

Penichrysol
(19)

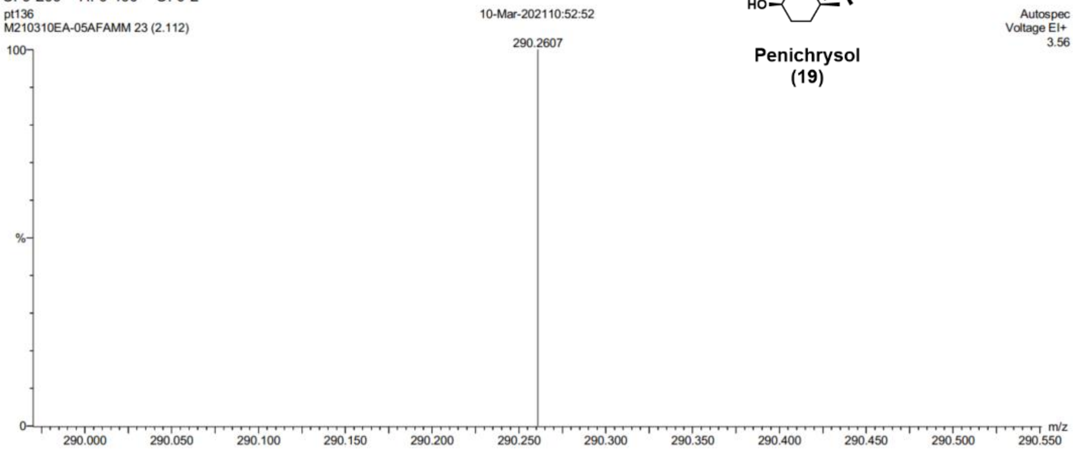

Minimum:				-1.5		
Maximum:		500.0	10.0	50.0		
Mass	Calc. Mass	mDa	PPM	DBE	i-FIT	Formula
290.2607	290.2610	-0.3	-1.0	4.0	5546025.5	C20 H34 O

Fig. S41. HR-EI-MS spectrum of compound **19** in $CDCl_3$

Single Mass Analysis (displaying only valid results)
Tolerance = 10.0 PPM / DBE: min = -1.5, max = 50.0
Selected filters: None

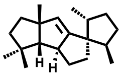

Spiroviolene
(21)

Monoisotopic Mass, Odd and Even Electron Ions
12 formula(e) evaluated with 1 results within limits (up to 50 closest results for each mass)
Elements Used:
C: 0-200 H: 0-400 O: 0-2

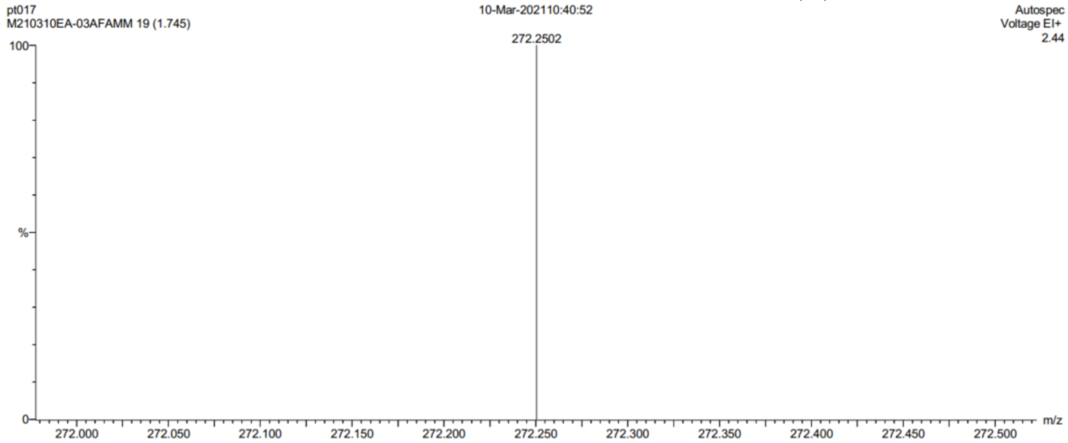

Minimum:				-1.5		
Maximum:		500.0	10.0	50.0		
Mass	Calc. Mass	mDa	PPM	DBE	i-FIT	Formula
272.2502	272.2504	-0.2	-0.7	5.0	5546025.5	C20 H32

Fig. S42. HR-EI-MS spectrum of compound **21** in $CDCl_3$

Single Mass Analysis (displaying only valid results)
Tolerance = 10.0 PPM / DBE: min = -1.5, max = 50.0
Selected filters: None

Monoisotopic Mass, Odd and Even Electron Ions
12 formula(e) evaluated with 1 results within limits (up to 50 closest results for each mass)
Elements Used:
C: 0-200 H: 0-400 O: 0-2

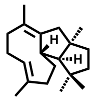

Variediene
(22)

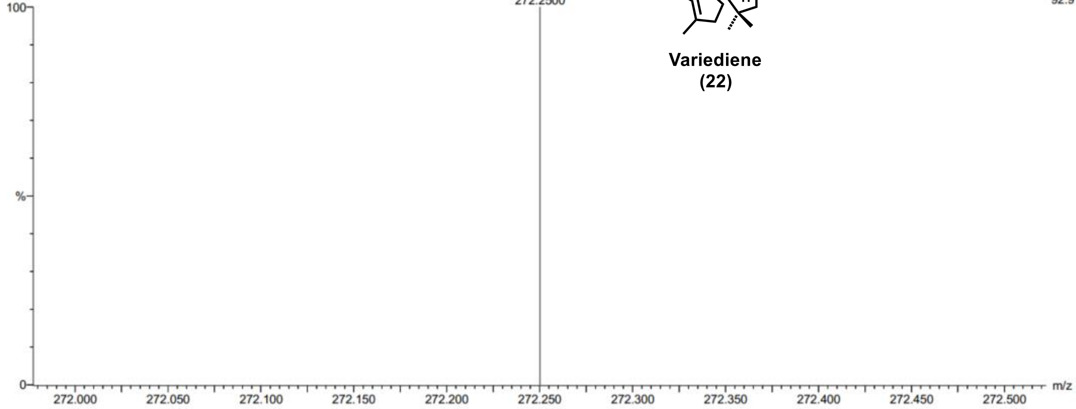

Minimum:				-1.5		
Maximum:		500.0	10.0	50.0		
Mass	Calc. Mass	mDa	PPM	DBE	i-FIT	Formula
272.2500	272.2504	-0.4	-1.5	5.0	5546060.0	C20 H32

Fig. S43. HR-EI-MS spectrum of compound **22** in $CDCl_3$

Single Mass Analysis (displaying only valid results)
Tolerance = 10.0 PPM / DBE: min = -1.5, max = 50.0
Selected filters: None

Monoisotopic Mass, Odd and Even Electron Ions
13 formula(e) evaluated with 1 results within limits (up to 50 closest results for each mass)
Elements Used:
C: 0-200 H: 0-400 O: 0-2

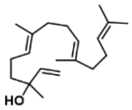

Geranyllinalool
(23)

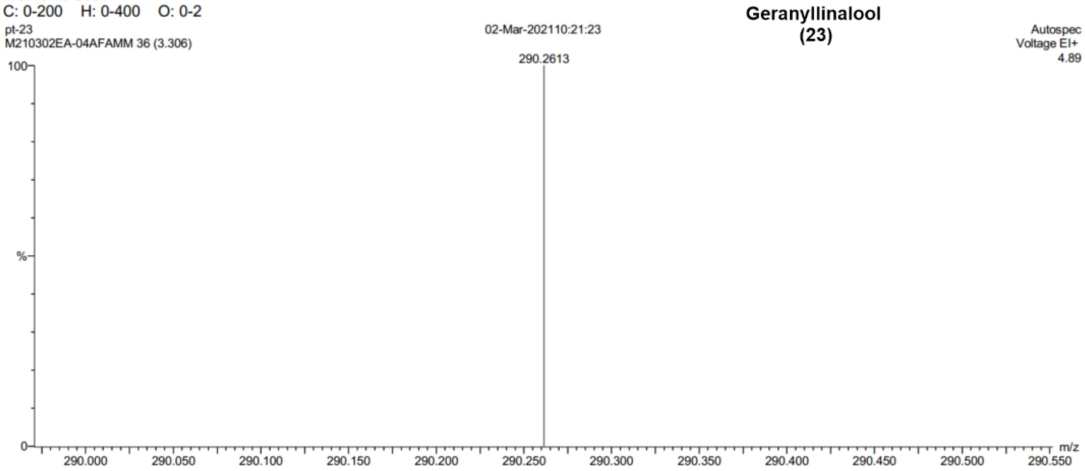

Minimum:				-1.5		
Maximum:		500.0	10.0	50.0		
Mass	Calc. Mass	mDa	PPM	DBE	i-FIT	Formula
290.2613	290.2610	0.3	1.0	4.0	5546025.5	C20 H34 O

Fig. S44. HR-EI-MS spectrum of compound **23** in $CDCl_3$.

Single Mass Analysis (displaying only valid results)
Tolerance = 10.0 PPM / DBE: min = -1.5, max = 50.0
Selected filters: None

Monoisotopic Mass, Odd and Even Electron Ions
13 formula(e) evaluated with 1 results within limits (up to 50 closest results for each mass)
Elements Used:
C: 0-200 H: 0-400 O: 0-2

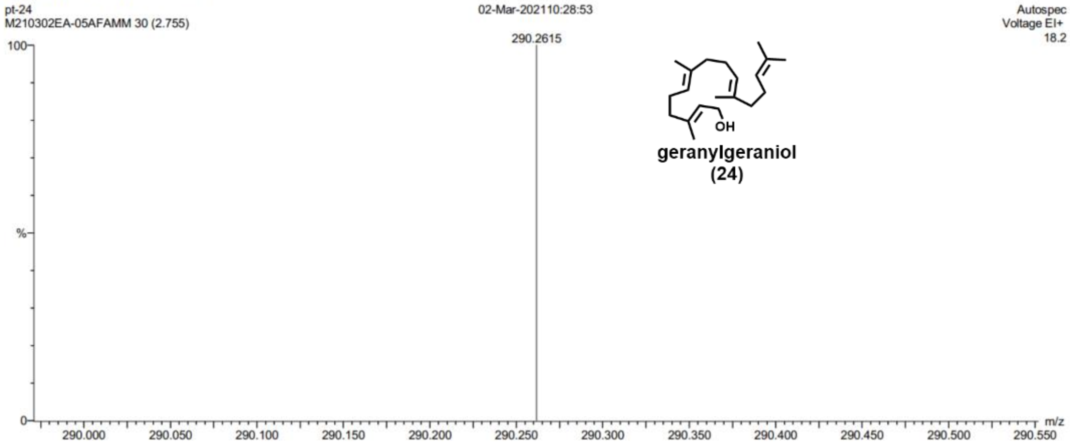

Minimum:				-1.5		
Maximum:		500.0	10.0	50.0		
Mass	Calc. Mass	mDa	PPM	DBE	i-FIT	Formula
290.2615	290.2610	0.5	1.7	4.0	5546028.0	C20 H34 O

Fig. S45. HMBC spectrum of compound **24** in $CDCl_3$.

Supplementary Tables:

Table S1. Detail information for 20 known PTTS enzymes.

Protein	Origin	Classification	Products	Ref
AbFS	*Alternaria brassicicola*	Clade II-B	Fusicoccadiene	(5)
AcldAS	*Aspergillus calidoustus*	Clade II-D	Asperterpenol A	(6)
AcOS	*Aspergillus clavatus*	Clade II-B	Opiobolin F, Ophiobolane 1, Ophiobolane 2, Clavaphyllene	(7)
BmTS1	*Bipolaris maydis*	Clade I-A	Bm1	(8)
BmTS2	*Bipolaris maydis*	Clade I-A	Bm2	(8)
BmTS3	*Bipolaris maydis*	Clade I-A	Bm3	(8)
BscA	*Pseudocercospora fijiensis*	Clade II-B	Fusicoccadiene	(9)
CgDS	*Colletotrichum gloeosporioides*	Clade II-D	Dolasta-1(15),8-diene, (1*R*)-δ-araneosene	(10)
EvAS	*Emericella variecolor*	Clade II-B	Astellifadiene	(11)
EvQS	*Emericella variecolor*	Clade I-F	Quiannulatene	(12)
EvSS	*Emericella variecolor*	Clade II-B	Stellata-2,6,19-triene	(13)
EvVS	*Emericella variecolor*	Clade II-C	Variediene	(14)
FgMS	*Fusarium graminearum*	Clade II-B	Mangicdiene Variecoltetraene	(15)
NfSS	*Neosartorya fischeri*	Clade I-A	Sesterfisherol	(16)
PaFS	*Phomopsis amygdali*	Clade II-B	Dusicoccadiene	(17)
PaPS	*Phomopsis amygdali*	Clade II-B	Phomopsene	(18)
PbSS	*Penicillium brasilianum*	Clade I-E	Sesterbrasiliatriene	(19)
PbTS1	*Phoma betae*	Clade I-A	Pb1	(8)
PcCS	*Penicillium chrysogenum*	Clade II-D	Penichrysol	(20)
PvPS	*Penicillium verruculosum*	Clade II-B	Preasperterpenoid A	(19)

Table S2. The general profiles of 74 PTTSs researched in this study.

PTTS	**Original number**	**Species**	**Lineage**
PTTS001	J3NSF9	*Gaeumannomyces tritici*	Sordariomyceta
PTTS002	A0A0C1E324	*Aspergillus ustus*	Eurotiomycetes
PTTS003	A0A0B8N1E4	*Talaromyces cellulolyticus*	Eurotiomycetes
PTTS004	G0SEK5	*Chaetomium thermophilum*	Sordariomyceta
PTTS005	Q2GYN3	*Chaetomium globosum*	Sordariomyceta
PTTS006	A0A0M8PAM1	*Penicillium nordicum*	Eurotiomycetes
PTTS007	A0A0D0BLM7	*Gymnopus luxurians*	Agaricales
PTTS008	A0A167CIH5	*Colletotrichum incanum*	Sordariomyceta
PTTS009	A0A162QA28	*Colletotrichum incanum*	Sordariomyceta
PTTS010	A0A0F4GLU2	*Zymoseptoria brevis*	Dothideomyceta
PTTS013	A0A100IAT3	*Aspergillus niger*	Eurotiomycetes
PTTS014	A0A177DNJ5	*Alternaria alternata*	Dothideomyceta
PTTS016	A0A177E1W6	*Alternaria alternata*	Dothideomyceta
PTTS017	A0A0U5GLI1	*Aspergillus calidoustus*	Eurotiomycetes
PTTS021	A0A135LYJ9	*Penicillium griseofulvum*	Eurotiomycetes
PTTS023	F7WBX5	*Sordaria macrospora*	Sordariomyceta
PTTS024	A0A0U5G0B1	*Aspergillus ustus*	Eurotiomycetes
PTTS026	A0A0U5GRW1	*Aspergillus calidoustus*	Sordariomyceta
PTTS027	A0A0U5GCR8	*Aspergillus ustus*	Eurotiomycetes
PTTS030	A0A0F7TBG7	*Penicillium brasilianum*	Eurotiomycetes
PTTS033	A0A010R1D7	*Colletotrichum fioriniae*	Sordariomyceta
PTTS035	A0A093XF11	*Pseudogymnoascus*	Sordariomyceta
PTTS036	N4V6D4	*Colletotrichum orbiculare*	Sordariomyceta
PTTS037	N4VHL7	*Colletotrichum orbiculare*	Sordariomyceta
PTTS039	A0A1B7XXV7	*Colletotrichum orbiculare*	Sordariomyceta
PTTS040	H1VXC5	*Colletotrichum higginsianum*	Sordariomyceta
PTTS041	H1VDU5	*Colletotrichum higginsianum*	Sordariomyceta
PTTS042	G2QU05	*Thermothielavioides terrestris*	Sordariomyceta
PTTS043	A0A0U1LL70	*Talaromyces islandicus*	Eurotiomycetes
PTTS044	A0A0U1LRS4	*Talaromyces islandicus*	Eurotiomycetes
PTTS045	A0A094HZN7	*Pseudogymnoascus*	Sordariomyceta
PTTS046	A0A093Z450	*Pseudogymnoascus*	Sordariomyceta
PTTS047	A0A017SNL7	*Aspergillus ruber*	Eurotiomycetes
PTTS049	S3E9C0	*Glarea lozoyensis*	Sordariomyceta
PTTS050	G4N1M9	*Pyricularia oryzae*	Sordariomyceta
PTTS051	W3XI54	*Pestalotiopsis fici*	Sordariomyceta
PTTS052	L7HT01	*Pyricularia grisea*	Sordariomyceta
PTTS054	T0KSF4	*Colletotrichum gloeosporioides*	Sordariomyceta
PTTS058	AIAI1_EUR_DPIG_10264	*Penicillium janthinellum*	Eurotiomycetes
PTTS059	AIAI1_DOT_EGUL_6851	*Pleomassaria siparia*	Dothideomyceta
PTTS060	AIAI1_EUR_HLIA_1630	*Penicillium canescens*	Eurotiomycetes

PTTS061	AIAI1_SOR_LATZ_7654	*Colletotrichum siamense*	Sordariomyceta
PTTS062	AIAI1_EUR_PPHY_2376	*Gymnascella aurantiaca*	Eurotiomycetes
PTTS065	AIAI1_LEC_CZOJ_335	*Xanthoria parietina*	Lecanoromycetes
PTTS066	AIAI1_SOR_EBHA_9358	*Thielavia arenaria*	Sordariomyceta
PTTS067	AIAI1_DOT_ERHT_3641	*Lophiostoma macrostomum*	Dothideomyceta
PTTS070	AIAI1_EUR_HLIA_4516	*Penicillium arizonense*	Eurotiomycetes
PTTS074	AIAI1_SOR_LATZ_6187	*Glomerella cingulata*	Sordariomyceta
PTTS078	AIAI1_DOT_NJXP_7686	*Westerdykella ornata*	Dothideomyceta
PTTS079	AIAI1_DOT_OWZC_3702	*Delitschia confertaspora*	Dothideomyceta
PTTS080	AIAI1_EUR_QHTQ_10144	*Penicillium glabrum*	Eurotiomycetes
PTTS086	AIAI1_SOR_WINP_3356	*Colletotrichum sublineola*	Sordariomyceta
PTTS087	AIAI1_DOT_XAAG_6412	*Pseudovirgaria hyperparasitica*	Dothideomyceta
PTTS089	AIAI1_SOR_XBMT_14221	*Colletotrichum caudatum*	Sordariomyceta
PTTS090	AIAI1_EUR_XJPO_12400	*Talaromyces verruculosus*	Eurotiomycetes
PTTS092	AIAI1_DOT_YXFC_1880	*Bimuria novae-zelandiae*	Dothideomyceta
PTTS093	AIAI1_DOT_ZFEH_9911	*Botryosphaeria dothidea*	Dothideomyceta
PTTS097	AIAI1_EUR_BSIJ_9281	*Bipolaris maydis*	Dothideomyceta
PTTS100	AIAI1_DOT_OCCK_11708	*Bipolaris maydis*	Dothideomyceta
PTTS103	A0A0C3HZQ7	*Oidiodendron maius*	Sordariomyceta
PTTS106	G4N8D6	*Pyricularia oryzae*	Sordariomyceta
PTTS107	AIAI1_DOT_JOFG_5695	*Baudoinia panamericana,*	Dothideomyceta
PTTS108	AIAI1_DOT_JSQJ_6579	*Macrophomina phaseolina*	Dothideomyceta
PTTS110	AIAI1_EUR_ODBE_6888	*Aspergillus brasiliensis*	Eurotiomycetes
PTTS114	AIAI1_AGA_RHJD_16091	*Gymnopus luxurians*	Agaricales
PTTS116	AIAI1_DOT_USYT_10235	*Glonium stellatum*	Dothideomyceta
PTTS119	AIAI1_EUR_WBMS_4798	*Aspergillus aculeatus*	Eurotiomycetes
PTTS122	AIAI1_DOT_ZLLL_9302	*Cenococcum geophilum*	Dothideomyceta
PTTS125	H1UY59	*Colletotrichum higginsianum*	Sordariomyceta
PTTS127	AIAI1_EUR_EYTM_12258	*Aspergillus niger*	Eurotiomycetes
PTTS128	AIAI1_EUR_GJUY_4531	*Aspergillus tubingensis*	Eurotiomycetes
PTTS129	AIAI1_SOR_GYZV_8783	*Beauveria bassiana*	Sordariomyceta
PTTS130	AIAI1_EUR_HPVK_8023	*Coccidioides immitis*	Eurotiomycetes
PTTS136	PENARI	*Penicillium arizonense*	Eurotiomycetes

Table S3. The general profiles of 34 PTTSs researched in this study.

Gene Name	Protein Name	Accession No.	Product	Type of product	Identity[a]	Identity[b]
PTTS008	CiGS	MW798200	*β*-Geranylfarnesene (**5**)<br>Pb1 (**11**)	sesterterpene	PbTS1 (43%)	PTTS036 (72%)<br>PTTS041 (79%)<br>PTTS061 (69%)
PTTS009	CiSS	MW798201	Sesterorbiculene (**2**)	sesterterpene	AcOS (29%)	PTTS037(70%)<br>PTTS054 (77%)
PTTS010	ZbSS	MW798202	Sesterevisene (**1**)	sesterterpene	EvQS (46%)	PTTS125 (45%)
PTTS013	AnGS	MW798203	Geranylfarnesol (**6**)<br>Geranyllinalool (**23**)	sesterterpene<br>diterpene	AcOS (28%)	
PTTS014	AaSS	MW798204	Sesterfisherol (**13**)	sesterterpene	NfSS (45%)	
PTTS017	AcSS	MW798205	Spiroviolene (**21**)	diterpene	AcOS (31%)	
PTTS021	PgGS	MW798206	Geranylfarnesol (**6**)	sesterterpene	AcOS (30%)	PTTS040 (31%)
PTTS023	SmVS	MW798207	Variediene (**22**)<br>Geranylgeraniol (**24**)	diterpene	EvVS (41%)	PTTS107 (41%)
PTTS024	AuOS	MW798208	Ophiobolin F (**15**)	sesterterpene	AcOS (65%)	PTTS100 (62%)
PTTS033	CfBS	MW798209	Bm3 (**14**)	sesterterpene	BmTS3 (71%)	PTTS108 (70%)<br>PTTS109 (40%)
PTTS036	CoFS	MW798210	Fusaproliferene (**10**)<br>Pb1 (**11**)	sesterterpene	PbTS1 (44%)	PTTS008 (72%)<br>PTTS041 (73%)<br>PTTS061 (73%)
PTTS037	CoSS	MW798211	Sesterorbiculene (**2**)	sesterterpene	BmTS (40%)	PTTS009 (70%)<br>PTTS054 (77%)
PTTS040	ChVS	MW798212	(-)-Variculatriene B (**7**)	sesterterpene	AcOS (31%)	PTTS050 (70%)<br>PTTS067 (60%)<br>PTTS086 (56%)<br>PTTS106 (67%)
PTTS041	ChPS	MW798213	Pb1 (**11**)	sesterterpene	PbTS1 (43%)	PTTS036 (73%)<br>PTTS008(79%)<br>PTTS061 (69%)
PTTS042	TtPS	MW798214	Preasperterpenoid (**18**)	sesterterpene	PvPS (36%)	PTTS090 (37%)
PTTS050	PoVS	MW798215	(-)-Variculatriene B (**7**)	sesterterpene	PaFS (28%)	PTTS040 (55%)<br>PTTS067 (62%)<br>PTTS086 (58%)<br>PTTS106 (55%)
PTTS051	PfVS	MW798216	Variculatriene A (**3**)<br>Clavaphyllene (**4**)<br>β-Geranylfarnesene (**5**)	sesterterpene	AcOS (31%)	
PTTS052	PgFS	MW798217	Fusicocccadiene (**17**)<br>Geranylfarnesol (**6**)	diterpene<br>sesterterpene	PaFS (57%)	
PTTS054	CgSS	MW798218	Sesterorbiculene (**2**)	sesterterpene	AcOS (30%)	PTTS009(70%)<br>PTTS037 (77%)
PTTS061	CsPS	MW798219	Pb1 (**11**)	sesterterpene	PbTS1 (45%)	PTTS036 (73%)<br>PTTS041 (72%)<br>PTTS008 (69%)
PTTS066	TaGS	MW798220	Geranylfarnesol (**6**)<br>Geranylgeraniol (**24**)	Sesterterpene<br>diterpene	AcOS (30%)	
PTTS067	LmVS	MW798221	(-)-Variculatriene B (**7**)	sesterterpene	AcOS (31%)	PTTS050 (62%)<br>PTTS040 (61%)<br>PTTS086 (60%)<br>PTTS106 (67%)
PTTS070	PaSS	MW798222	Sesterbrasiliatriene (**12**)	sesterterpene	PbSS (67%)	
PTTS086	CsVS	MW798223	(-)-Variculatriene B (**7**)	sesterterpene	PaFS (30%)	PTTS050 (58%)<br>PTTS067 (60%)<br>PTTS040 (56%)

						PTTS106 (55%)
PTTS087	PhPS	MW798224	Penichrysol (**19**)<br>Phomopsene (**20**)	diterpene	PaPS (55%)	
PTTS090	TvPS	MW798225	Preasperterpenoid (**18**)	sesterterpene	PvPS (94%)	PTTS042 (37%)
PTTS100	BmOS	MW798226	Ophiobolin F (**15**)	sesterterpene	AcOS (64%)	PTTS024 (62%)
PTTS106	PoVS	MW798227	(-)-Variculatriene B (**7**)	sesterterpene	PaFS (29%)	PTTS050 (55%)<br>PTTS067 (67%)<br>PTTS086 (56%)<br>PTTS040 (67%)
PTTS107	BpVS	MW798228	GJ1012 C (**16**)<br>Variediene (**22**)	diterpene	EvVS (40%)	PTTS023 (41%)
PTTS108	MpBS	MW798229	Bm3 (**14**)	sesterterpene	BmTS3 (72%)	PTTS109 (42%)<br>PTTS033 (70%)
PTTS110	AbVS	MW798230	GJ1012 C (**16**)<br>Variediene (**22**)	diterpene	EvVS (64%)	PTTS119 (66%)
PTTS119	AaGS	MW798231	Geranylfarnesol (**6**)<br>GJ1012 C (**16**)<br>Variediene (**22**)	sesterterpene<br>diterpene	EvVS (60%)	PTTS110 (66%)
PTTS125	ChBS	MW798232	Brassiteraene A (**8**)<br>Brassiteraene B (**9**)	sesterterpene	AcOS (32%)	
PTTS136	PaCS	MW798233	Penichrysol (**19**)	diterpene	PcCS (71%)	

Note: The number of products is consistent with Fig. 3; [a] represents the identity between PTTSs in this work and characterized previously; [b] represents the identity among PTTSs in this work.

Table S4. The data for structural characterization of 24 compounds.

Compound	HR-EI-MS	1D-NMR	2D-NMR	[α] spectrum	GC-MS spectrum	UV spectrum	IR spectrum
Sesterevisene (**1**)	●	●	●	●	●	●	●
Sesterorbiculene (**2**)	●	●	●	●	●	●	●
β-Geranylfarnesene (**5**)	●	●	●	⃠	●	⃠	⃠
Geranylfarnesol (**6**)	●	●	●	⃠	●	⃠	⃠
(-)-Variculatriene B (**7**)	●	●	●	●	●	⃠	⃠
Ophiobolin F (**15**)	●	●	●	●	●	⃠	⃠
Preasperterpenoid (**18**)	●	●	●	●	●	⃠	⃠
Geranyllinalool (**23**)	●	●	●	⃠	●	⃠	⃠
Geranylgeraniol (**24**)	●	●	●	⃠	●	⃠	⃠
Pb1 (**11**)	●	●	●	●	●	⃠	⃠
Sesterbrasiliatriene (**12**)	●	●	●	●	●	⃠	⃠
Sesterfisherol (**13**)	●	●	●	●	●	⃠	⃠
Bm3 (**14**)	●	●	●	●	●	⃠	⃠
GJ1012 C (**16**)	●	●	●	●	●	⃠	⃠
Fusicocccadiene (**17**)	●	●	●	●	●	⃠	⃠
Penichrysol (**19**)	●	●	●	●	●	⃠	⃠
Spiroviolene (**21**)	●	●	●	●	●	⃠	⃠
Variediene (**22**)	●	●	●	●	●	⃠	⃠
Variculatriene A (**3**)	⃠	⃠	⃠	⃠	●	⃠	⃠
Clavaphyllene (**4**)	⃠	⃠	⃠	⃠	●	⃠	⃠
Brassiteraene A (**8**)	⃠	⃠	⃠	⃠	●	⃠	⃠
Brassiteraene B (**9**)	⃠	⃠	⃠	⃠	●	⃠	⃠
Fusaproliferene (**10**)	⃠	⃠	⃠	⃠	●	⃠	⃠
Phomopsene (**20**)	⃠	⃠	⃠	⃠	●	⃠	⃠

Note: The orange underline represents two new compounds characterized in this paper; ● represents the collected data; ⃠ represent data that was not collected.

Table S5. ^{1}H, ^{13}C, ^{1}H-^{1}H COSY and HMBC NMR data of Sesterevisene (**1**)[a]

Num	δ_H	δ_C	^{1}H-^{1}H COSY	HMBC
1	1.79 (m)	45.1	H2	C3, C12, C16
	1.14 (m)			
2	1.96 (td, 12.2, 3.2)	44.6	H1, H12	C4
3	-	141.7		
4	5.12 (td, 8.8, 1.5)	118.6	H5	
5	2.72 (m)	28.6	H6, H4	C3, C6, C7
	1.82 (overlap)			
6	2.15 (m)	51.3	H5	C21
7	1.47 (overlap)	40.9		
8	1.75 (m)	33.8	H9	C21
	1.06 (m)			
9	2.26 (dd, 16.2, 8.0)	30.9	H8	C6, C7, C11
	2.06 (m)			
10	-	138.3		
11	-	128.1		
12	2.87 (td, 11.5, 3.5)	48.0	H13	C10, C14
13	1.69 (m)	29.3	H12	
	1.46 (m)			
14	1.61 (overlap)	52.1		C1
15	-	42.1		
16	1.48 (overlap)	40.8	H17	
	1.12 (m)			
17	1.83 (overlap)	28.2	H16	C15
	1.55 (m)			
18	1.64 (m)	47.1		C15
19	1.60 (overlap)	31.6	H24, H25	C24, C25
20	1.59 (overlap)	25.8		C2, C4
21	0.98 (d, 6.4)	18.4	H7	C6, C8
22	1.53 (s)	14.6		C10, C22
23	0.85 (s)	19.3		C1, C14, C16
24	0.81 (d, 6.2)	22.6	H19	C18, C19
25	0.90 (d, 6.2)	23.9	H19	C18, C19

[a] ^{1}H and ^{13}C NMR data were recorded at 500 MHz and 125 MHz in $CDCl_3$, respectively.

Table S6. ^{1}H, ^{13}C, ^{1}H-^{1}H COSY and HMBC NMR data of Sesterorbiculene (**2**)[a]

Num	δ_H	δ_C	^{1}H-^{1}H COSY	HMBC
1	2.03 (dd, 14.0, 6.7)	39.6	H2	C14
	1.80 (dd, 14.0, 8.0)			
2	5.17 (m)	122.5	H1	C15, C20
3	-	135.2		
4	2,16 (m)	39.8		
5	2.27 (m)	24.8	H6	C3, C6
	2.16 (m)			
6	5.05 (m)	123.8	H5	C21
7	-	134.7		
8	2.76 (m)	38.3	H9	C6, C7, C9
9	5.45 (dt, 11.4, 8.3)	125.7	H8, H10	C11
10	5.87 (d, 11.4)	134.9	H9	C12, C22
11	-	129.7		
12	5.37 (tt, 6.4, 1.4)	129.6	H13	C10
13	1.98 (td, 6.1, 3.1)	31.9	H12, H14	
14	1.52 (m)	46.3	H13	C12, C19
15	-	44.9		
16	1.43 (m)	41.2		C1, C17, C18
	1.38 (m)			
17	1.57 (m)	25.6		
	1.29 (m)			
18	1.40 (m)	54.9		
19	1.61 (m)	32.0		
20	1.57 (s)	15.6		C4
21	1.62 (overlap)	17.4		C6, C7
22	1.62 (overlap)	17.0		C10, C22
23	0.84 (overlap)	22.3		C1
24	0.91 (d, 6.8)	22.5	H19	C18
25	0.84 (overlap)	18.9	H19	C18

[a] ^{1}H and ^{13}C NMR data were recorded at 500 MHz and 125 MHz in $CDCl_3$, respectively

Table S7. ^1H and ^{13}C NMR data of *β*-Geranylfarnesene (**5**)[a] and Geranylfarnesol (**6**)[a]

Num	*β*-Geranylfarnesene (5)[a]		Geranylfarnesol (6)[a]	
	δ_H	δ_C	δ_H	δ_C
1	5.22 (d, 10.8)<br>5.04 (d, 10.8)	113.3	4.15 (d, 6.9)	59.4
2	6.36 (dd, 17.6, 10.8)	139.2	5.43 (m)	124.4
3		146.3		139.9
4	2.22 (m)	31.6	2.12-2.03 (m)	39.7
5	2.02-2.08 (m)	26.8	1.97-2.00 (m)	26.3
6	5.10-5.17 (m)	124.2	5.09-5.12 (m)	124.3
7		135.6		135.4
8	1.98-2.08 (m)	39.9	2.12-2.03 (m)	39.6
9	2.02-2.08 (m)	26.9	1.97-2.00 (m)	26.8
10	5.11 (overlap)	124.4	5.09-5.12 (m)	124.2
11		135.1		135.0
12	1.99-2.08 (m)	39.9	2.12-2.03 (m)	39.7
13	2.02-2.08 (m)	26.9	1.97-2.00 (m)	26.7
14	5.11 (overlap)	124.5	5.09-5.12 (m)	123.8
15		135.2		134.9
16	1.99-2.08 (m)	39.9	2.12-2.03 (m)	39.6
17	2.08-2.21 (m)	27.0	1.97-2.00 (m)	26.6
18	5.11 (overlap)	124.6	5.09-5.12 (m)	123.3
19		131.5		131.3
20	4.99 (s)<br>4.98 (s)	115.9	1.67-1.69 (m)	16.0
21	1.57-1.59 (m)	16.3	1.58-1.61 (m)	16.0
22	1.57-1.59 (m)	16.2	1.58-1.61 (m)	16.0
23	1.57-1.59 (m)	16.3	1.58-1.61 (m)	16.3
24	1.66 (s)	25.9	1.67-1.69 (m)	25.1
25	1.57-1.59 (m)	17.9	1.58-1.61 (m)	17.7

[a] ^1H and ^{13}C NMR data were recorded at 500 MHz and 125 MHz in $CDCl_3$, respectively.

Table S8. ^{1}H and ^{13}CNMR data of (-)-Variculatriene B (**7**)[a] and Pb1 (**11**)[c].

Num	(-)-Variculatriene B (7)[a]		Pb1 (11)[c]	
	δ_H	δ_C	δ_H	δ_C
1	2.25 (m)	139.1	1.38 (m)	43.7
	1.67 (m)			
2	5.11 (m)	126.1	5.14 (m)	126.6
3	-	25.1	-	139.3
4	2.21 (m)	46.9	2.90 (m)	37.5
	1.91 (m)		2.41 (m)	
5	2.48 (m)	42.7	4.88 (dd,4.9)	122.5
6	-	36.7	-	142.4
7	2.79 (m)	25.3	2.73 (m)	32.4
8	2.11 (m)	46.4	1.92 (m)	33.5
	1.39 (m)		1.47 (m)	
9	2.21 (m)	27.5	1.26 (m)	27.0
	1.90 (m)			
10	-	23.2	1.84 (m)	50.5
11	-	19.2	-	133.2
12	5.48 (m)	23.8	5.48 (d, 11.5)	128.4
13	1.90 (m)	25.6	2.06 (m)	25.5
	1.56 (m)			
14	1.65 (m)	24.4	1.71 (m)	47.5
15	-	20.8	-	44.6
16	1.64 (m)	139.1	1.39 (m)	42.6
	1.28 (m)			
17	1.77 (m)	126.1	1.49 (m)	26.3
	1.55 (m)			
18	2.06 (m)	25.1	1.84 (m)	49.4
19	2.00 (m)	46.9	1.62 (m)	27.2
20	1.69 (m)	42.7	1.69 (s)	18.6
21	1.01 (d, 6.9)	36.7	1.01 (d, 7.0)	22.5
22	1.69 (m)	25.3	1.58 (s)	20.3
23	0.83 (s)	46.4	0.96 (d, 7.1)	24.6
24	0.97 (d, 6.7)	27.5	0.85 (d, 6.4)	21.1
25	0.91 (d, 6.7)	23.2	0.96 (d, 7.1)	24.2

[a] ^{1}H and ^{13}C NMR data were recorded at 500 MHz and 125 MHz in $CDCl_3$, respectively.

[a] ^{1}H and ^{13}C NMR data were recorded at 400 MHz and 100 MHz in $CDCl_3$, respectively.

Table S9. ^{1}H and ^{13}CNMR data of Sesterbrasiliatriene (**12**)[a] and Sesterfisherol (**13**)[a].

Num	Sesterbrasiliatriene (12)[c]		Sesterfisherol (13)[c]	
	δ_H	δ_C	δ_H	δ_C
1	1.94 (m)	42.1	*	43.7
	1.84 (m)		*	
2	5.27 (t, 7.5)	123.1	1.43 (m)	44.8
3	-	136.3	1.63 (m)	33.8
4	2.18 (m)	37.6	*	35.8
	1.97 (m)		*	
5	1.91 (m)	27.9	1.45 (m)	26.2
	1.07 (m)		1.03 (m)	
6	2.48 (m)	50.0	3.68 (brs)	43.7
7	-	157.1	2.18 (m)	38.4
8	2.47(m)	32.4	1.70 (m)	29.1
			1.30 (m)	
9	1.90 (m)	27.9	2.36 (m)	32.0
	1.67(m)		2.21 (m)	
10	2.93 (td, 7.1, 3.5)	51.3	-	143.0
11	-	134.5	-	132.8
12	5.09 (dd, 10.3, 4.0)	130.0	-	78.4
13	1.93 (m)	29.1	2.16 (m)	39.2
			1.61 (m)	
14	1.41 (m)	49.5	1.93 (m)	44.1
15	-	45.2	-	41.5
16	1.50 (m)	43.5	1.47 (m)	41.1
	1.35 (m)		1.15 (m)	
17	1.48 (m)	23.6	1.86 (m)	28.4
	1.23 (m)		1.57 (m)	
18	1.37 (m)	54.1	1.67 (m)	47.1
19	1.72 (m)	28.4	1.62 (m)	31.5
20	0.93 (d, 6.9)	22.8	0.89 (d, 7.2)	23.8
21	0.82 (d, 6.8)	16.8	0.96 (d, 6.8)	15.2
22	0.83 (s)	23.1	1.72 (s)	17.9
23	1.43 (s)	14.1	0.82(s)	18.6
24	4.80 (m)	103.0	0.86 (d, 6.2)	22.6
	4.75 (m)			
25	1.57 (s)	16.2	0.93 (d, 6.0)	24.0

[c] ^{1}H and ^{13}C NMR data were recorded at 400 MHz and 100 MHz in $CDCl_3$, respectively. The signals with asterisk were not observed possibly due to significant broadning.

Table S10. ^{1}H and ^{13}CNMR data of Bm3 (**14**)[c] and Ophiobolin F (**15**)[a].

Num	Bm3 (14)[c]		Ophiobolin F (15)[a]	
	δ_H	δ_C	δ_H	δ_C
1	1.83 (m)	42.4	1.94 (m)	25.7
	2.16 (m)		2.00 (m)	
2	5.25 (m)	125.5	1.62 (m)	54.2
3	-	134.6		80.2
4	2.13 (m)	39.8	1.68 (m)	42.0
	2.06 (m)		1.96 (m)	
5	2.08 (m)	24.0	1.43 (m)	23.8
	2.17 (m)		1.54 (m)	
6	5.22 (m)	124.9	3.15 (t, 8.3)	42.5
7	-	132.7		136.6
8	1.78 (m)	35.1	5.64 (t, 8.3)	139.2
	2.05 (m)			
9	1.63 (m)	30.0	2.13 (m)	23.7
	1.81 (m)			
10	3.96 (dd, 3.7, 9.7)	77.2	1.68 (m)	55.9
11	-	134.6		43.9
12	5.23(m)	132.5	1.34 (m)	43.7
			1.44 (m)	
13	1.87 (m)	22.8	1.32 (m)	36.8
	1.98 (m)		1.44 (m)	
14	1.82 (m)	46.0	2.30 (m)	45.9
15	-	46.2	1.68 (m)	33.4
16	1.36(m)	41.6	1.27 (m)	37.8
	1.52 (m)		1.40 (m)	
17	1.16 (m)	28.2	1.95 (m)	26.7
	1.66 (m)			
18	1.66 (m)	52.1	5.28 (t, 6.9)	125.6
19	1.52 (m)	29.2		130.9
20	1.61 (s)	15.8	1.05 (s)	29.0
21	1.60 (s)	15.4	2.03 (s)	21.9
22	1.57 (s)	10.8	0.85 (s)	19.1
23	0.97 (s)	24.0	0.83 (d, 6.9)	17.2
24	0.86 (d, 6.3)	21.8	1.62 (s)	17.8
25	0.92 (d, 6.3)	22.4	1.71 (s)	26.1

[a] ^{1}H and ^{13}C NMR data were recorded at 500 MHz and 125 MHz in $CDCl_3$, respectively.

[c] ^{1}H and ^{13}C NMR data were recorded at 400 MHz and 100 MHz in $CDCl_3$, respectively

Table S11. ^{1}H and ^{13}C NMR data of GJ1012 C (**16**)[b] and Fusicocccadiene (**17**)[c].

Num	GJ1012 C (16)[b]		Fusicocccadiene (17)[c]	
	δ_H	δ_C	δ_H	δ_C
1	1.59 (m)	44.8	2.29 (m)	39.3
	1.23 (m)		1.95 (m)	
2	2.26 (m)	53.0	-	137.2
3	-	148.5	-	132.1
4	1.97 (m)	38.9	2.23 (m)	36.7
	1.66 (m)		2.16 (m)	
5	2.12 (m)	28.6	1.71 (m)	22.4
	1.65 (m)			
6	5.22 (dd, 11.1, 4.0)	123.9	2.48 (m)	54.7
7	-	136.2	1.97 (m)	29.9
8	1.67 (m)	41.2	1.43 (m)	32.2
9	1.56 (m)	33.0	2.01 (m)	21.3
	1.14 (m)		1.91 (m)	
10	2.05 (m)	45.5	-	139.6
11	-	47.9	-	51.6
12	1.58 (m)	40.9	1.64 (m)	38.6
13	1.41 (m)	42.1	2.18 (m)	26.7
14	1.44 (m)	72.5	-	140.5
15	-	42.9	2.63 (m)	27.0
16	4.87 (brs)	111.4	1.63 (s)	15.5
	4.86 (brs)			
17	1.52 (s)	18.9	0.86 (d, 7.0)	21.2
18	1.16 (s)	31.6	0.93 (s)	26.7
19	1.04 (s)	24.4	0.92 (d, 7.0)	21.0
20	1.21 (s)	32.7	0.98 (d, 7.0)	21.3

[b] ^{1}H and ^{13}C NMR data were recorded at 600 MHz and 150 MHz in $CDCl_3$, respectively.

[c] ^{1}H and ^{13}C NMR data were recorded at 400 MHz and 100 MHz in $CDCl_3$, respectively.

Table S12. ^1H and ^{13}C NMR data of Preasperterpenoid (**18**)[a] and Penichrysol (**19**)[c].

Num	Preasperterpenoid (18)[a]		Penichrysol (19)[c]	
	δ_H	δ_C	δ_H	δ_C
1	2.38 (m)<br>1.64 (m)	47.3	3.60 (dd, 5.3, 3.4)	73.7
2	-	135.9	1.49 (m)<br>1.66 (m)	29.7
3	-	131.1	1.23 (m)<br>1.40 (m)	27.2
4	2.38 (m)<br>2.07 (m)	37.9	1.48 (m)	35.0
5	1.91 (m)<br>1.81 (m)	26.2	-	60.6
6	2.11 (d, 9.3)	54.4	2.30 (m)	53.9
7		22.7	1.32 (m)<br>1.94 (m)	31.7
8	0.52 (dd, 8.4, 4.1)<br>0.31 (m)	24.9	1.33 (m)<br>1.86 (m)	37.6
9	2.26 (dd, 16.2, 8.0)<br>2.06 (m)	29.5	-	49.2
10	1.20 (m)	47.2	1.72 (m)<br>1.93 (m)	43.9
11	-	39.4	-	53.7
12	1.61 (m)<br>1.31 (m)	39.4	1.72 (m)<br>1.94 (m)	40.9
13	1.46 (m)<br>1.32 (m)	36.1	1.41 (m)<br>1.60 (m)	40.5
14	1.19 (m)	51.1	-	42.4
15	-	42.9	1.47 (m)	74.2
16	1.37 (m)<br>1.10 (m)	40.1	0.95 (d,7.2)	17.7
17	1.63 (m)<br>1.46 (m)	22.3	1.12 (s)	22.1
18	1.77 (m)	45.4	1.24 (s)	31.7
19	2.36 (m)	28.4	1.01 (s)	31.6
20	0.87 (m)	23.3	0.97 (s)	27.1
21	0.79 (d, 7.0)	15.2	-	-
22	0.75 (s)	17.6	-	-
23	0.86 (m)	20.4	-	-
24	0.89 (s)	20.9	-	-
25	1.66 (s)	13.6	-	-

[a] ^1H and ^{13}C NMR data were recorded at 500 MHz and 125 MHz in $CDCl_3$, respectively.

[c] ^1H and ^{13}C NMR data were recorded at 400 MHz and 100 MHz in $CDCl_3$, respectively.

Table S13. ^{1}H and ^{13}C NMR data of Spiroviolene (**21**)^c and Variediene (**22**)^b.

Num	Spiroviolene (21)^c		Variediene (22)^b	
	δ_H	δ_C	δ_H	δ_C
1	4.77 ((d, 2.8)	128.5	1.95 (m)<br>1.47 (m)	45.5
2	-	148.5	-	143.8
3	1.64 (m)	44.4	-	120.8
4	1.76 (m)<br>1.36 (m)	30.9	1.65 (m)	38.2
5	1.73 (m)<br>1.33 (m)	30.3	1.93 (m)	24.9
6	1.84 (m)	46.2	5.22 (dd, 11.1, 3.9)	126.8
7	-	53.3	-	138.8
8	1.92 (td,12.7, 6.9)<br>1.69 (m)	39.1	2.14 (m)<br>2.08 (m)	40.9
9	1.68 (m)<br>1.04 (m)	32.6	2.30 (m)<br>1.69 (m)	41.2
10	2.68 (dtd, 12.6, 6.4, 2.9)	58.9	2.22 (m)	44.8
11	-	63.3	-	48.5
12	1.64 (m)<br>1.54 (m)	38.2	1.47 (m)<br>1.39 (m)	41.7
13	1.55 (m)<br>1.38 (m)	40.4	1.24 (m)	41.3
14	1.52 (m)	65.7	1.29 (s)	68.3
15	-	41.0	-	43.2
16	1.02 (s)	28.9	1.59 (s)	21.4
17	0.98 (s)	25.9	1.36 (s)	16.1
18	1.29 (s)	32.1	1.22 (s)	31.3
19	0.87 (d, 6.8)	14.8	0.95 (s)	32.2
20	0.84 (d, 6.7)	14.7	0.78 (s)	24.4

^{b} ^{1}H and ^{13}C NMR data were recorded at 600 MHz and 150 MHz in $CDCl_3$, respectively.
^{c} ^{1}H and ^{13}C NMR data were recorded at 400 MHz and 100 MHz in $CDCl_3$, respectively.

Table S14. ^{1}H and ^{13}CNMR data of Geranyllinalool (**23**)[b] and Geranylgeraniol (**24**)[a].

Num	Geranyllinalool (23) [b]		Geranylgeraniol (24)[a]	
	δ_H	δ_C	δ_H	δ_C
1	5.22 (dd, 17.3, 1.3)<br>5.06 (dd, 10.8, 1.3)	111.9	4.14 (m)	59.4
2	5.92 (dd, 17.3, 10.8)	145.2	5.40 (m)	124.4
3	-	73.8	-	139.8
4	1.57 (m)	42.3	1.93-2.05 (m)	39.7
5	2.03 (m)	22.9	1.97-2.14 (m)	26.7
6	5.08-5.15 (m)	124.4	5.09 (m)	124.1
7	-	135.9	-	135.4
8	2.06 (m)<br>1.97 (m)	39.9	1.93-2.05 (m)	39.7
9	2.06 (m)	26.8	1.97-2.14 (m)	26.6
10	5.09-5.12 (m)	124.3	5.09 (m)	123.7
11	-	135.3	-	135.0
12	2.06 (m)<br>1.97 (m)	39.9	1.93-2.05 (m)	39.5
13	2.06 (m)<br>-	26.9	1.97-2.14 (m)	26.3
14	5.08-5.15 (m)	124.6	5.09 (m)	123.3
15	-	131.5	-	131.3
16	1.28-1.67 (m)	28.1	1.67 (m)	16.3
17	1.59 (m)	16.2	1.58 (m)	16.0
18	1.59 (m)	16.3	1.58 (m)	16.0
19	1.68 (m)	25.9	1.67 (m)	25.7
25	1.59 (m)	17.9	1.58 (m)	17.7

[a] ^{1}H and ^{13}C NMR data were recorded at 500 MHz and 125 MHz in $CDCl_3$, respectively.

[b] ^{1}H and ^{13}C NMR data were recorded at 600 MHz and 150 MHz in $CDCl_3$, respectively.

Table S15. Experimental and calculated ^{13}C-NMR chemical shifts of **1**-18*S* and **1**-18*R*.

No.	δexptl.	**1**-18*S*-$\delta_{calcd.}$	**1**-18*S*-$\delta_{calcd.}$ (corrected)	**1**-18*R*-$\delta_{calcd.}$	**1**-18*R*-$\delta_{calcd.}$ (corrected)
1	45.1	45.3	46.4	46.6	44.2
2	44.6	47.1	48.2	46.5	44.1
3	141.7	141.6	142.1	143.1	142.4
4	118.6	118.5	119.2	118.1	116.9
5	28.6	32.6	33.8	31.6	28.9
6	51.3	53.6	54.7	53.5	51.2
7	40.9	42.9	43.9	42.7	40.2
8	33.8	35.9	37.0	36.8	34.2
9	30.9	34.1	35.2	34.2	31.5
10	138.3	138.5	139.1	139.0	138.3
11	128.1	130.7	131.3	130.1	129.2
12	48.0	49.1	50.1	49.3	46.9
13	29.3	32.5	33.6	33.0	30.4
14	52.1	50.2	51.2	52.9	50.6
15	42.1	44.2	45.3	43.7	41.2
16	40.8	42.7	43.7	45.0	42.5
17	28.2	25.7	26.9	32.9	30.3
18	47.1	46.7	47.8	49.4	47.0
19	31.6	30.6	31.7	36.3	33.7
20	25.8	28.7	29.9	28.9	26.1
21	18.4	19.7	20.9	20.0	17.1
22	14.6	18.3	19.5	18.6	15.7
23	19.3	22.5	23.7	22.7	19.9
24	23.9	23.1	18.1	25.0	20.8
25	22.6	16.9	24.3	23.6	22.2
R^2		0.99644		0.99905	
MAE		2.04		2.35	
CMAE		2.60		0.98	

R^2: correlation coefficient parameter; MAE: mean absolute error; CMAE: corrected mean absolute error.

Table S16. Experimental and calculated ^{1}H-NMR chemical shifts of **1**-18*S* and **1**-18*R*.

No.	δexptl.	**1**-18*S*-$\delta_{calcd.}$	**1**-18*S*-$\delta_{calcd.}$ (corrected)	**1**-18*R*-$\delta_{calcd.}$	**1**-18*R*-$\delta_{calcd.}$ (corrected)
1a	1.14	1.25	1.28	1.13	1.14
1b	1.79	1.87	1.84	1.66	1.62
2	1.96	2.21	2.14	2.18	2.08
4	5.12	5.53	5.10	5.63	5.17
5a	1.82	1.83	1.80	1.83	1.77
5b	2.72	2.72	2.59	2.82	2.66
6	2.15	2.25	2.18	2.28	2.17
7	1.47	1.61	1.61	1.57	1.54
8a	1.06	1.09	1.14	1.02	1.05
8b	1.75	1.66	1.65	1.76	1.71
9a	2.06	2.22	2.15	2.18	2.08
9b	2.26	2.36	2.27	2.34	2.22
12	2.87	3.07	2.90	2.95	2.78
13a	1.46	1.29	1.32	1.63	1.59
13b	1.69	1.44	1.45	1.66	1.62
14	1.61	1.37	1.39	1.60	1.57
16a	1.12	0.92	0.99	0.89	0.93
16b	1.48	1.52	1.53	1.47	1.45
17a	1.55	1.40	1.42	1.68	1.64
17b	1.83	1.53	1.53	1.84	1.78
18	1.64	1.68	1.67	1.55	1.52
19	1.60	1.72	1.70	1.68	1.64
20	1.59	1.67	1.66	1.69	1.65
21	0.98	0.97	1.03	0.93	0.97
22	1.53	1.63	1.62	1.65	1.61
23	0.85	0.94	1.01	0.98	1.02
24	0.90	0.88	0.87	0.95	0.87
25	0.81	0.78	0.95	0.82	0.98
R^2		0.97748		0.98913	
MAE		0.126		0.098	
CMAE		0.126		0.070	

R^2: correlation coefficient parameter; MAE: mean absolute error; CMAE: corrected mean absolute error.

Table S17. DP4+ results of **1**-18*S* and **1**-18*R*.

	**1**-18*S*	**1**-18*R*
DP4+ (H data)	0.00%	100%
DP4+ (C data)	0.00%	100%
DP4+ (all data)	0.00%	100%

Table S18. Conformational analysis of the M06-2X-D3/def2-SVP optimized conformers of **1**-18*S* in the gas phase (T=298.15 K)

Conformer	E[a] (Hartree)	C[b] (Hartree)	G[c] (kcal/mol)	ΔG[d] (kcal/mol)	Population[e]
**1**-18*S*-1	-975.021553	0.54717	-611826.024508	0.0	86.34%
**1**-18*S*-2	-975.019217	0.548474	-611824.558668	1.46584	7.26%
**1**-18*S*-3	-975.019098	0.548199	-611824.483995	1.540512	6.40%

[a]Electronic energy obtained at M06-2X-D3/def2-SVP level of theory; [b]Thermal correction to Gibbs free energy obtained at M06-2X-D3/def2-SVP level of theory; [c]Gibbs free energy (E + C); [d]The relative Gibbs free energy; [e]The Boltzmann distribution of each conformer.

Table S19. Atomic coordinates (Å) of **1**-18*S*-1 obtained at the M06-2X-D3/def2-SVP level of theory in the gas phase.

C	-0.229601	-0.010460	0.445018	H	1.337852	-0.043440	1.981489
C	-0.189045	-1.526921	0.110586	H	3.929673	-2.344084	-1.476822
C	0.954701	-1.837406	-0.879282	H	3.090828	-1.027220	-2.318914
C	2.307938	-1.377898	-0.331038	H	4.847340	0.337446	-1.572033
C	2.217155	0.121977	0.009038	H	5.336139	-0.781150	-0.307327
C	1.126442	0.446547	1.016413	H	3.934572	0.168106	1.290371
C	3.469319	-1.354411	-1.337709	H	-3.010491	-0.114973	-2.571178
C	4.458732	-0.304003	-0.767362	H	-1.369136	0.081645	-2.014813
C	3.665898	0.528752	0.282148	H	-3.303863	-2.160937	-1.377546
C	-1.412570	0.333452	1.323520	H	-4.275229	0.148725	1.985766
C	-2.574183	0.747702	0.796452	H	-3.600776	1.711153	2.480450
C	-2.897886	0.922821	-0.689150	H	-4.503618	2.821655	0.507455
C	-2.381354	-0.161571	-1.668667	H	-5.818695	1.685785	0.882452
C	-2.417764	-1.566641	-1.128320	H	-0.549879	0.855862	3.243452
C	-1.493645	-2.154543	-0.355144	H	-2.184647	0.161347	3.349401
C	-3.818220	1.080329	1.607085	H	-0.789091	-0.877081	2.996295
C	-4.756893	1.751755	0.604370	H	3.391234	2.463178	1.138230
C	-4.437324	1.048496	-0.713888	H	5.627776	3.383009	0.661763
C	-1.235037	0.112970	2.803628	H	5.789862	1.784520	1.429409
H	-2.489966	1.897480	-1.023342	H	6.042146	1.963305	-0.321955
C	3.949092	2.040417	0.283738	H	3.760024	3.819747	-0.948434
C	5.434366	2.308439	0.529194	H	2.372978	2.723070	-1.078693
C	3.466908	2.759775	-0.975790	H	3.907522	2.320312	-1.885182
H	1.923783	0.606832	-0.941743	H	-6.093089	1.816740	-1.893496
H	-0.336136	0.543603	-0.495707	H	-4.597061	2.772678	-2.014773
H	0.057335	-2.040337	1.057317	H	-4.738811	1.219783	-2.871977
C	-4.996426	1.748465	-1.942442	H	-0.810201	-4.192462	-0.119306
H	-4.849790	0.022930	-0.659364	H	-1.797828	-3.592843	1.219259
C	-1.687496	-3.570310	0.122553	H	-2.574953	-4.036072	-0.325389
C	2.721929	-2.243840	0.869554	H	3.757570	-2.040369	1.177513
H	0.971747	-2.915817	-1.109858	H	2.082145	-2.097524	1.750870
H	0.745277	-1.312746	-1.828655	H	2.667345	-3.307727	0.590506
H	1.086117	1.531257	1.214389	-	-	-	-

Table S20. Atomic coordinates (Å) of **1**-18*S*-2 obtained at the M06-2X-D3/def2-SVP level of theory in the gas phase.

C	-0.210842	-0.001804	0.403980	H	1.390217	0.055304	1.901688
C	-0.180279	-1.538591	0.189467	H	3.888422	-2.521390	-1.349641
C	0.966538	-1.934911	-0.764187	H	3.117539	-1.210084	-2.266753
C	2.315623	-1.406830	-0.270893	H	5.043401	-0.001771	-1.569451
C	2.227142	0.126208	-0.095955	H	5.258362	-0.964035	-0.111783
C	1.152625	0.499025	0.920991	H	3.956974	0.519168	1.143243
C	3.475010	-1.504913	-1.267147	H	-3.034993	-0.317717	-2.556423
C	4.498872	-0.477267	-0.740359	H	-1.384943	-0.086973	-2.040597
C	3.688951	0.582906	0.072340	H	-3.310262	-2.274634	-1.219200
C	-1.379745	0.408088	1.272878	H	-4.234847	0.274692	1.985257
C	-2.548867	0.783834	0.733672	H	-3.552778	1.867205	2.357747
C	-2.891953	0.851766	-0.756392	H	-4.482589	2.835774	0.324345
C	-2.392000	-0.302033	-1.662553	H	-5.791714	1.729244	0.794884
C	-2.421592	-1.664659	-1.022868	H	-0.733328	-0.677976	3.021476
C	-1.488112	-2.197147	-0.221312	H	-0.487441	1.067628	3.136614
C	-3.781628	1.175660	1.534838	H	-2.121764	0.387676	3.316821
C	-4.733557	1.775315	0.499275	H	3.906709	2.014931	-1.499464
C	-4.431021	0.981605	-0.770908	H	3.033838	3.037404	1.258731
C	-1.180222	0.296405	2.762464	H	3.409646	4.080896	-0.131430
H	-2.485201	1.798149	-1.164828	H	2.042431	2.955713	-0.220307
C	4.010381	2.011695	-0.398130	H	5.734209	3.350281	-0.458520
C	3.066807	3.076205	0.157690	H	6.167122	1.625049	-0.448347
C	5.457397	2.365968	-0.053316	H	5.586720	2.404570	1.040454
H	1.891012	0.517881	-1.077839	H	-6.097588	1.668773	-1.986241
H	-0.333408	0.481240	-0.573478	H	-4.603156	2.615357	-2.181410
H	0.059085	-1.972708	1.176117	H	-4.749065	1.009210	-2.932934
C	-5.001274	1.598089	-2.038701	H	-2.572785	-4.068110	-0.043927
H	-4.846216	-0.036104	-0.641778	H	-0.806365	-4.216336	0.150851
C	-1.678437	-3.575053	0.358647	H	-1.775185	-3.518858	1.455483
C	2.732508	-2.128055	1.021258	H	3.711390	-1.781181	1.381518
H	0.998539	-3.031291	-0.880501	H	2.017934	-1.996069	1.843823
H	0.753624	-1.515118	-1.763413	H	2.815299	-3.208396	0.825354
H	1.091478	1.584751	1.078250	-	-	-	-

Table S21. Atomic coordinates (Å) of **1**-18*S*-3 obtained at the M06-2X-D3/def2-SVP level of theory in the gas phase.

C	0.149897	-0.012307	0.393417	H	-1.451076	-0.179849	1.886419
C	0.088018	1.529180	0.229944	H	-3.985899	2.455132	-1.380862
C	-1.028539	1.917987	-0.760016	H	-3.095669	1.264872	-2.346944
C	-2.387533	1.382505	-0.303999	H	-4.804119	-0.248107	-1.822733
C	-2.290741	-0.147265	-0.114444	H	-5.421616	0.735238	-0.500106
C	-1.209118	-0.553740	0.877160	H	-4.059608	-0.288633	1.094852
C	-3.513203	1.462143	-1.347159	H	3.009835	0.497315	-2.506248
C	-4.497008	0.335622	-0.941612	H	1.354498	0.219561	-2.028914
C	-3.752029	-0.575963	0.075040	H	3.234818	2.389834	-1.060867
C	1.316602	-0.432630	1.260021	H	4.171032	-0.274134	1.994424
C	2.497761	-0.765297	0.718802	H	3.508420	-1.886887	2.312817
C	2.853855	-0.763367	-0.769722	H	4.455913	-2.774523	0.248828
C	2.353083	0.429013	-1.624993	H	5.751436	-1.673598	0.767892
C	2.351905	1.757513	-0.916417	H	0.426423	-1.178958	3.093188
C	1.392806	2.231763	-0.108815	H	2.041994	-0.465626	3.308970
C	3.731197	-1.166088	1.513756	H	0.629680	0.575357	3.039019
C	4.695717	-1.718791	0.463562	H	-5.176648	-2.114395	-0.284214
C	4.393754	-0.881841	-0.778673	H	-2.297152	-2.809491	-1.078742
C	1.102291	-0.378487	2.750780	H	-3.766573	-3.734416	-1.448698
H	2.456056	-1.691787	-1.225170	H	-3.509473	-2.143028	-2.197257
C	-4.090852	-2.070748	-0.084590	H	-4.174917	-3.909880	1.081405
C	-3.378402	-2.721132	-1.269536	H	-4.398786	-2.431376	2.045124
C	-3.846984	-2.866170	1.198584	H	-2.780831	-2.882455	1.467631
H	-1.972240	-0.533108	-1.102174	H	6.070986	-1.506268	-2.014317
H	0.291015	-0.457423	-0.599954	H	4.588797	-2.464694	-2.242810
H	-0.207559	1.924841	1.218344	H	4.714281	-0.832466	-2.939614
C	4.973927	-1.447856	-2.065536	H	0.662874	4.215450	0.342763
H	4.800829	0.133460	-0.610748	H	1.603557	3.469441	1.640967
C	1.540901	3.580397	0.545875	H	2.437666	4.109385	0.198011
C	-2.849460	2.113829	0.967382	H	-3.901105	1.895182	1.201085
H	-1.063852	3.013728	-0.881173	H	-2.254938	1.857115	1.855356
H	-0.779967	1.496432	-1.750364	H	-2.769693	3.201617	0.815232
H	-1.137165	-1.650142	0.954067	-	-	-	-

Table S22. Conformational analysis of the M06-2X-D3/def2-SVP optimized conformers of **1**-18*R* in the gas phase (T=298.15 K)

Conformer	E[a] (Hartree)	C[b] (Hartree)	G[c] (kcal/mol)	ΔG[d] (kcal/mol)	Population[e]
**1**-18*R*-1	-975.014394	0.547698	-611821.532235	0.0	91.45%
**1**-18*R*-2	-975.012158	0.549399	-611820.129145	1.40309	8.55%

[a]Electronic energy obtained at M06-2X-D3/def2-SVP level of theory; [b]Thermal correction to Gibbs free energy obtained at M06-2X-D3/def2-SVP level of theory; [c]Gibbs free energy (E + C); [d]The relative Gibbs free energy; [e]The Boltzmann distribution of each conformer.

Table S23. Atomic coordinates (Å) of **1**-18*R*-1 obtained at the M06-2X-D3/def2-SVP level of theory in the gas phase.

C	-0.247408	-0.205826	-0.140787	H	1.461082	-0.897080	-1.317772
C	-0.155335	1.288147	-0.544306	H	3.740007	2.787510	1.030086
C	0.891427	2.014884	0.323564	H	2.797020	1.889662	2.239110
C	2.267356	1.351375	0.215034	H	4.848966	0.585119	2.232291
C	2.135633	-0.136371	0.605542	H	5.109227	0.886580	0.519234
C	1.131557	-0.882110	-0.265975	H	3.541038	-1.310972	1.754526
C	3.293870	1.807400	1.258949	H	-3.353486	1.295231	2.089663
C	4.331572	0.670931	1.266746	H	-1.673748	0.860781	1.913112
C	3.581014	-0.642592	0.876668	H	-3.372274	2.553090	0.059294
C	-1.339853	-0.919075	-0.907697	H	-4.087538	-1.061448	-1.955158
C	-2.578855	-1.037485	-0.408313	H	-3.461257	-2.674864	-1.574278
C	-3.085025	-0.499278	0.932304	H	-4.658918	-2.732559	0.546477
C	-2.620300	0.916322	1.360768	H	-5.844869	-1.900036	-0.483456
C	-2.503671	1.908511	0.233457	H	-1.863222	-1.693520	-2.871092
C	-1.461190	2.064953	-0.594650	H	-0.438371	-0.635689	-2.847037
C	-3.738215	-1.708204	-1.130784	H	-0.310946	-2.290063	-2.239101
C	-4.829698	-1.830843	-0.066707	H	4.369833	-0.849645	-1.133759
C	-4.621366	-0.595079	0.806530	H	4.319682	-3.401639	-1.164543
C	-0.979642	-1.414775	-2.284946	H	2.707505	-2.679952	-1.067017
H	-2.781814	-1.206356	1.729669	H	3.465604	-3.356462	0.391480
C	4.326037	-1.450625	-0.208347	H	6.308745	-2.300324	-0.520286
C	3.657923	-2.789396	-0.534092	H	6.332025	-0.821437	0.460333
C	5.761301	-1.734912	0.247783	H	5.750305	-2.344446	1.166584
H	1.673143	-0.102224	1.609682	H	-5.052697	-1.520210	2.717161
H	-0.492219	-0.260785	0.927377	H	-5.173494	0.254684	2.742803
H	0.225708	1.297258	-1.580935	H	-6.450673	-0.708024	1.974391
C	-5.364409	-0.640053	2.132315	H	-1.492940	2.594946	-2.680602
H	-4.955414	0.288806	0.230110	H	-2.409834	3.716150	-1.637755
C	-1.509130	3.091319	-1.696236	H	-0.626492	3.750717	-1.661729
C	2.844816	1.569407	-1.192722	H	3.896222	1.259322	-1.257648
H	0.952307	3.077528	0.034289	H	2.296262	1.023948	-1.973084
H	0.552148	1.990902	1.374411	H	2.803485	2.640740	-1.445145
H	1.033947	-1.931016	0.054722	-	-	-	-

Table S24. Atomic coordinates (Å) of **1**-18*R*-2 obtained at the M06-2X-D3/def2-SVP level of theory in the gas phase.

C	-0.266169	-0.224619	-0.158496	H	1.453613	-0.846206	-1.366621
C	-0.151324	1.301663	-0.424342	H	3.816403	2.606643	1.197932
C	0.903355	1.952959	0.499337	H	2.874463	1.659301	2.370429
C	2.271198	1.282673	0.324875	H	4.907069	0.328241	2.214813
C	2.087902	-0.220979	0.622471	H	5.077085	0.645473	0.492255
C	1.108901	-0.904122	-0.322077	H	3.377003	-1.400076	1.859534
C	3.340373	1.629309	1.371629	H	-3.366383	1.107113	2.181356
C	4.336449	0.453230	1.284199	H	-1.688783	0.676457	1.975914
C	3.490235	-0.805558	0.938568	H	-3.363028	2.535936	0.268399
C	-1.361504	-0.849080	-0.994081	H	-4.117612	-0.863569	-2.044201
C	-2.602813	-1.001399	-0.509999	H	-3.498456	-2.510224	-1.829077
C	-3.103432	-0.584068	0.875678	H	-4.684826	-2.768762	0.285355
C	-2.631905	0.786651	1.426092	H	-5.872556	-1.838582	-0.654783
C	-2.500594	1.871362	0.390089	H	-0.334508	-2.099781	-2.437818
C	-1.450146	2.092352	-0.413049	H	-1.881386	-1.441648	-3.020851
C	-3.767720	-1.591064	-1.290255	H	-0.450697	-0.397845	-2.897448
C	-4.855347	-1.812179	-0.238077	H	3.379902	-2.601255	-0.197791
C	-4.640263	-0.665734	0.748494	H	5.802830	-3.183508	-0.111241
C	-0.998807	-1.220622	-2.408910	H	5.166093	-2.874714	1.523317
H	-2.798115	-1.360224	1.605057	H	6.152540	-1.643481	0.702250
C	4.120128	-1.792512	-0.064261	H	4.844544	-2.051362	-2.086242
C	5.378241	-2.412218	0.547947	H	3.560515	-0.833119	-1.959828
C	4.442671	-1.245625	-1.453917	H	5.210583	-0.457168	-1.409732
H	1.574700	-0.218828	1.601310	H	-5.059050	-1.768929	2.564425
H	-0.524505	-0.371826	0.897234	H	-5.181939	-0.005109	2.760559
H	0.231354	1.397080	-1.455989	H	-6.462958	-0.891943	1.911467
C	-5.375787	-0.837568	2.068443	H	-0.597335	3.864221	-1.314998
H	-4.977175	0.269078	0.261181	H	-1.459220	2.811323	-2.443185
C	-1.482253	3.214842	-1.417433	H	-2.380630	3.836364	-1.309650
C	2.824915	1.614694	-1.069648	H	2.273713	1.127433	-1.885860
H	0.968351	3.033554	0.290002	H	2.761854	2.702435	-1.231912
H	0.568480	1.849803	1.546638	H	3.878351	1.329346	-1.172564
H	1.008599	-1.975408	-0.080009	-	-	-	-

Table S25. Details of primers used in this study.

Primer name	Primer sequence (5'-3')
PTTS001-F	CAATTGAGCTGATCCAGTACTGCCAACAACTGTAACATCTCTAACAG
PTTS001-R	GAAAATTCAATATAAGCTTGCCACCATGACTTCTAATTGGATC
PTTS001V-R	CTGTTAGAGATGTTACAGTTGTTGGCAGTACTGGATCAGCTCAATTG
PTTS001V-F	GATCCAATTAGAAGTCATGGTGGCAAGCTTATATTGAATTTTC
PTTS002-F	GAAAATTCAATATAAGCTTGCCACCATGGAAGCTGCATTGAGAG
PTTS002-R	CAATTGAGCTGATCCAGTACTGCCTAAACCCAAACCAACCAACAAC
PTTS002V-R	CTCTCAATGCAGCTTCCATGGTGGCAAGCTTATATTGAATTTTC
PTTS002V-F	GTTGTTGGTTGGTTTGGGTTTAGGCAGTACTGGATCAGCTCAATTG
PTTS003-F	CAATATAAGCTTGCCACCATGTACGATAATGTTGTTGAATCTGCTG
PTTS003-R	GACAATTGAGCTGATCCAGTACTGCCTCTAACTTTTAATCTAGAAAC
PTTS003V-R	CAGCAGATTCAACAACATTATCGTACATGGTGGCAAGCTTATATTG
PTTS003V-F	GTTTCTAGATTAAAAGTTAGAGGCAGTACTGGATCAGCTCAATTGTC
PTTS004-F	GAAAATTCAATATAAGCTTGCCACCATGGAATACAGATACTCTGATC
PTTS004-R	CAATTGAGCTGATCCAGTACTGCCAACTTTCAATTTATCCAACAAAG
PTTS004V-R	GATCAGAGTATCTGTATTCCATGGTGGCAAGCTTATATTGAATTTTC
PTTS004V-F	CTTTGTTGGATAAATTGAAAGTTGGCAGTACTGGATCAGCTCAATTG
PTTS005-F	GAAAATTCAATATAAGCTTGCCACCATGGATCATCCAAATTCTC
PTTS005-R	CAATTGAGCTGATCCAGTACTGCCAACTTTTAATGCTTCTAACAAAC
PTTS005V-R	GAGAATTTGGATGATCCATGGTGGCAAGCTTATATTGAATTTTC
PTTS005V-F	GTTTGTTAGAAGCATTAAAAGTTGGCAGTACTGGATCAGCTCAATTG
PTTS006-F	GAAAATTCAATATAAGCTTGCCACCATGACTATGGGTAAAGAAATTG
PTTS006-R	CAATTGAGCTGATCCAGTACTGCCTTTTTGAATAGCTTCTTCCATTG
PTTS006V-R	CAATTTCTTTACCCATAGTCATGGTGGCAAGCTTATATTGAATTTTC
PTTS006V-F	CAATGGAAGAAGCTATTCAAAAAGGCAGTACTGGATCAGCTCAATTG
PTTS007-F	GAAAATTCAATATAAGCTTGCCACCATGGAGTTTAGATACTCAAC
PTTS007-R	CAATTGAGCTGATCCAGTACTGCCAACTCTTAACAATTCCCATAAC
PTTS007V-R	GTTGAGTATCTAAACTCCATGGTGGCAAGCTTATATTGAATTTTC
PTTS007V-F	GTTATGGGAATTGTTAAGAGTTGGCAGTACTGGATCAGCTCAATTG
PTTS008-F	GAGCTGATCCAGTACTGCCCAAAGTAATATCTCTAACAGATAATC
PTTS008-R	CAATATAAGCTTGCCACCATGGAATCATTATGGGAACATTCTG
PTTS008V-R	CTGTTAGAGATATTACTTTGGGCAGTACTGGATCAGCTCAATTG
PTTS008V-F	GAATGTTCCCATAATGATTCCATGGTGGCAAGCTTATATTGAATTTTC
PTTS009-F	CAATTGAGCTGATCCAGTACTGCCTAAACCTGCTGGAGCAAC
PTTS009-R	ATTCAATATAAGCTTGCCACCATGGCTGAATCAATGTGGAAATATTC
PTTS009V-R	GTTGCTCCAGCAGGTTTAGGCAGTACTGGATCAGCTCAATTG
PTTS009V-F	CCACATTGATTCAGCCATGGTGGCAAGCTTATATTGAATTTTC
PTTS0010-F	GAGCTGATCCAGTACTGCCAATCCATAATCTTCTCAAAACTAATTC
PTTS0010-R	CAATATAAGCTTGCCACCATGGCAGAATTTGCTATTCCAGTTC
PTTS0010V-R	GTTTTGAGAAGATTATGGATTGGCAGTACTGGATCAGCTCAATTG
PTTS0010V-F	CTGGAATAGCAAATTCTGCCATGGTGGCAAGCTTATATTGAATTTTC
PTTS0013-F	CAATTGAGCTGATCCAGTACTGCCCATTTCTCTCAATCTATCC

PTTS0013-R	GAAAATTCAATATAAGCTTGCCACCATGTCTCCAGTTGATATTTC
PTTS0013V-R	GGATAGATTGAGAGAAATGGGCAGTACTGGATCAGCTCAATTG
PTTS0013V-F	GAAATATCAACTGGAGACATGGTGGCAAGCTTATATTGAATTTTC
PTTS0014-F	GAGCTGATCCAGTACTGCCAGCTAAACCTCTAACAGAGATACC
PTTS0014-R	CAATATAAGCTTGCCACCATGGAAGATCAAATTTTCATTCAATCAAG
PTTS0014V-R	TCTCTGTTAGAGGTTTAGCTGGCAGTACTGGATCAGCTCAATTG
PTTS0014V-F	CTTGATTGAATGAAAATTTGATCTTCCATGGTGGCAAGCTTATATTG
PTTS0016-F	GAAAATTCAATATAAGCTTGCCACCATGGATAGACCAAAACAAGC
PTTS0016-R	CAATTGAGCTGATCCAGTACTGCCAACTTCCAATCTTTGCAACAATG
PTTS0016V-R	GCTTGTTTTGGTCTATCCATGGTGGCAAGCTTATATTGAATTTTC
PTTS0016V-F	CATTGTTGCAAAGATTGGAAGTTGGCAGTACTGGATCAGCTCAATTG
PTTS0017-F	GAAAATTCAATATAAGCTTGCCACCATGGGTACTATGGGTTCTG
PTTS0017-R	GAGCTGATCCAGTACTGCCAGACAATGAAGATGTCCAAGCAGTTTC
PTTS0017V-R	CAGAACCCATAGTACCCATGGTGGCAAGCTTATATTGAATTTTC
PTTS0017V-F	CTGCTTGGACATCTTCATTGTCTGGCAGTACTGGATCAGCTCAATTG
PTTS0021-F	CAATTGAGCTGATCCAGTACTGCCATGACCCTTTTTACCTGG
PTTS0021-R	CAATATAAGCTTGCCACCATGGATTTGCCAACTTCTGAATTG
PTTS0021V-R	CAGGTAAAAAGGGTCATGGCAGTACTGGATCAGCTCAATTG
PTTS0021V-F	CAATTCAGAAGTTGGCAAATCCATGGTGGCAAGCTTATATTG
PTTS0023-F	GAGCTGATCCAGTACTGCCAACTGACAATTTAGCCATACACATTC
PTTS0023-R	CAATATAAGCTTGCCACCATGGATATGTTGGATGGTCATACTTCTG
PTTS0023V-R	GTGTATGGCTAAATTGTCAGTTGGCAGTACTGGATCAGCTCAATTG
PTTS0023V-F	GAAGTATGACCATCCAACATATCCATGGTGGCAAGCTTATATTG
PTTS0024-F	GAGCTGATCCAGTACTGCCAACTTTTAACAATTCTAACATCATTC
PTTS0024-R	CAATATAAGCTTGCCACCATGGAATACAAATACTCTACTATCGTTG
PTTS0024V-R	CAACGATAGTAGAGTATTTGTATTCCATGGTGGCAAGCTTATATTG
PTTS0024V-F	GATGTTAGAATTGTTAAAAGTTGGCAGTACTGGATCAGCTCAATTG
PTTS0026-F	CAATTGAGCTGATCCAGTACTGCCCAAACCTAAACCAACCAAC
PTTS0026-R	GAAAATTCAATATAAGCTTGCCACCATGGATGCTGCATTAAGAG
PTTS0026V-R	CTCTTAATGCAGCATCCATGGTGGCAAGCTTATATTGAATTTTC
PTTS0026V-F	GTTGGTTGGTTTAGGTTTGGGCAGTACTGGATCAGCTCAATTG
PTTS0027-F	GAAAATTCAATATAAGCTTGCCACCATGTACACTTTGGATGATTTC
PTTS0027-R	GAGCTGATCCAGTACTGCCAACTTTCAATAACAACAAGAAAATTC
PTTS0027V-R	GAAATCATCCAAAGTGTACATGGTGGCAAGCTTATATTGAATTTTC
PTTS0027V-F	GAATTTTCTTGTTGTTATTGAAAGTTGGCAGTACTGGATCAGCTC
PTTS0030-F	GAAAATTCAATATAAGCTTGCCACCATGAAAAATTCTAGATATTTG
PTTS0030-R	CAATTGAGCTGATCCAGTACTGCCTAAACCCAAACCCAACAAC
PTTS0030V-R	CAAATATCTAGAATTTTTCATGGTGGCAAGCTTATATTGAATTTTC
PTTS0030V-F	GTTGTTGGGTTTGGGTTTAGGCAGTACTGGATCAGCTCAATTG
PTTS0033-F	GAAAATTCAATATAAGCTTGCCACCATGGATCCATTGTTGTACAG
PTTS0033-R	CAATTGAGCTGATCCAGTACTGCCACCCAAAGTTGCGATACCCTTTG
PTTS0033V-R	CTGTACAACAATGGATCCATGGTGGCAAGCTTATATTGAATTTTC
PTTS0033V-F	CAAAGGGTATCGCAACTTTGGGTGGCAGTACTGGATCAGCTCAATTG

PTTS0035-F	GAAAATTCAATATAAGCTTGCCACCATGAAGTCTCAAGTTACTTTC
PTTS0035-R	CAATTGAGCTGATCCAGTACTGCCTGGTGGCAAGATATCTGACAAC
PTTS0035V-R	GAAAGTAACTTGAGACTTCATGGTGGCAAGCTTATATTGAATTTTC
PTTS0035V-F	GTTGTCAGATATCTTGCCACCAGGCAGTACTGGATCAGCTCAATTG
PTTS0036-F	CAATTGAGCTGATCCAGTACTGCCTAAAGAAACATCTCTAACTG
PTTS0036-R	GAAAATTCAATATAAGCTTGCCACCATGACTACAATTTGGGAACATTG
PTTS0036V-R	GTTCCCAAATTGTAGTCATGGTGGCAAGCTTATATTGAATTTTC
PTTS0036V-F	CAGTTAGAGATGTTTCTTTAGGCAGTACTGGATCAGCTCAATTG
PTTS0037-F	CAATTGAGCTGATCCAGTACTGCCCAAACCAGCTGGAACAAC
PTTS0037-R	GAAAATTCAATATAAGCTTGCCACCATGGCATCTGAAATGTGG
PTTS0037V-R	CCACATTTCAGATGCCATGGTGGCAAGCTTATATTGAATTTTC
PTTS0037V-F	GTTGTTCCAGCTGGTTTGGGCAGTACTGGATCAGCTCAATTG
PTTS0039-F	GAAAATTCAATATAAGCTTGCCACCATGTTGTCTTATTCAACAGTTG
PTTS0039-R	CAATTGAGCTGATCCAGTACTGCCAATTCTTAACAATTCTAAC
PTTS0039V-R	CAACTGTTGAATAAGACAACATGGTGGCAAGCTTATATTGAATTTTC
PTTS0039V-F	GTTAGAATTGTTAAGAATTGGCAGTACTGGATCAGCTCAATTG
PTTS0040-F	CAATATAAGCTTGCCACCATGTTGTCTGAAGAAGAATTATATC
PTTS0040-R	CAATTGAGCTGATCCAGTACTGCCAACATCAATTCTTAAAGATTCC
PTTS0040V-R	GATATAATTCTTCTTCAGACAACATGGTGGCAAGCTTATATTG
PTTS0040V-F	GGAATCTTTAAGAATTGATGTTGGCAGTACTGGATCAGCTCAATTG
PTTS0041-F	GAGCTGATCCAGTACTGCCAACATCAATTCTTAAAGATTCC
PTTS0041-R	CAATATAAGCTTGCCACCATGTTGTCTGAAGAAGAATTATATC
PTTS0041V-R	GATATAATTCTTCTTCAGACAACATGGTGGCAAGCTTATATTG
PTTS0041V-F	GAATCTTTAAGAATTGATGTTGGCAGTACTGGATCAGCTCAATTG
PTTS042-R	CAATTGAGCTGATCCAGTACTGCCAACTGCAACAGCTTCTAACAAAG
PTTS042-F	CAATATAAGCTTGCCACCATGGAATCTAATAATTCAGTTTTGGC
PTTS042V-R	CTGAATTATTAGATTCCATGGTGGCAAGCTTATATTGAATTTTC
PTTS042V-F	GTTAGAAGCTGTTGCAGTTGGCAGTACTGGATCAGCTCAATTG
PTTS043-F	GAGCTGATCCAGTACTGCCTTTAACATGCAATCTTTTCAATAAC
PTTS043-R	CAATATAAGCTTGCCACCATGGTTTCTTTAGAAGTTTTCTTGTTG
PTTS043V-R	GAAAAGATTGCATGTTAAAGGCAGTACTGGATCAGCTCAATTGTC
PTTS043V-F	CAACAAGAAAACTTCTAAAGAAACCATGGTGGCAAGCTTATATTG
PTTS044-F	GAGCTGATCCAGTACTGCCTTCAATTTTTAACAAAATCAAAAATGC
PTTS044-R	CAATATAAGCTTGCCACCATGTCTATCTTGGATGATTTCCCATAC
PTTS044V-R	GCATTTTTGATTTTGTTAAAAATTGAAGGCAGTACTGGATCAGCTC
PTTS044V-F	GTATGGGAAATCATCCAAGATAGACATGGTGGCAAGCTTATATTG
PTTS045-F	CAATTGAGCTGATCCAGTACTGCCAACTTTCAAAGTTGATAACAAAG
PTTS045-R	CAATATAAGCTTGCCACCATGGAATATCATTACTCTCATTTGGTTG
PTTS045V-R	GTTATCAACTTTGAAAGTTGGCAGTACTGGATCAGCTCAATTGTCTG
PTTS045V-F	CAACCAAATGAGAGTAATGATATTCCATGGTGGCAAGCTTATATTG
PTTS046-F	GAGCTGATCCAGTACTGCCTGGTGGTGGAATATCAGACAAGAAC
PTTS046-R	CAATATAAGCTTGCCACCATGAAATCTCAAGATACTTCACAAAAGAG
PTTS046V-R	GTTCTTGTCTGATATTCCACCACCAGGCAGTACTGGATCAGCTC

PTTS046V-F	CTCTTTTGTGAAGTATCTTGAGATTTCATGGTGGCAAGCTTATATTG
PTTS047-F	GAGCTGATCCAGTACTGCCAACTTCTAAAACACCTAACAAAGATTC
PTTS047-R	CAATATAAGCTTGCCACCATGGAATACAGATTTTCATACTTGATGG
PTTS047V-R	CTTTGTTAGGTGTTTTAGAAGTTGGCAGTACTGGATCAGCTCAATTG
PTTS047V-F	CCATCAAGTATGAAAATCTGTATTCCATGGTGGCAAGCTTATATTG
PTTS049-F	ATTGAGCTGATCCaGTaCTGCCTAATCTCAACATTTCAACAAACAATC
PTTS049-R	GAAAATTCAATATAAGCTTgccaccATGGCTACTGCACCAGCTTATG
PTTS049V-R	GTTGAAATGTTGAGATTAGGCAGtACtGGATCAGCTCAATTGTCTGTTG
PTTS049V-F	GGTGCAGTAGCCATggtggcAAGCTTATATTGAATTTTCAAAAATTCTTA
PTTS050-F	GAGCTGATCCAGTACTGCCAACTCTCAATGTTTCAACCAACAATC
PTTS050-R	CAATATAAGCTTGCCACCATGATGGATAGAAGAGATATCTATCC
PTTS050V-R	GTTGGTTGAAACATTGAGAGTTGGCAGTACTGGATCAGCTCAATTG
PTTS050V-F	GGATAGATATCTCTTCTATCCATCATGGTGGCAAGCTTATATTG
PTTS051-F	GAGCTGATCCAGTACTGCCAACTTCCAATTCTAATCTCTTTTGAAC
PTTS051-R	CAATATAAGCTTGCCACCATGGCTGGTACTAGATCTTCAAGACC
PTTS051V-R	GTTCAAAAGAGATTAGAATTGGAAGTTGGCAGTACTGGATCAGCTC
PTTS051V-F	GGTCTTGAAGATCTAGTACCAGCCATGGTGGCAAGCTTATATTG
PTTS052-F	GAGCTGATCCAGTACTGCCAACTCTTAACAATTCTAAAATAACTTCC
PTTS052-R	CAATATAAGCTTGCCACCATGTTTGTTCATTCAGAAGCATTGGATG
PTTS052V-R	GGAAGTTATTTTAGAATTGTTAAGAGTTGGCAGTACTGGATCAGCTC
PTTS052V-F	GCATCCAATGCTTCTGAATGAACAAACATGGTGGCAAGCTTATATTG
PTTS054-F	CAATTGAGCTGATCCAGTACTGCCAACACCTAATGGAACAATAACTG
PTTS054-R	CAATATAAGCTTGCCACCATGGCTGAAGAAATGTGGAAATATTC
PTTS054V-R	CAGTTATTGTTCCATTAGGTGTTGGCAGTACTGGATCAGCTCAATTG
PTTS054V-F	GAATATTTCCACATTTCTTCAGCCATGGTGGCAAGCTTATATTG
PTTS058-F	GAGCTGATCCAGTACTGCCAGTAACTGTTGCCAATAATCTATTAGC
PTTS058-R	CAATATAAGCTTGCCACCATGGAATTGTTATATTCTGATCCAGTTG
PTTS058V-R	GATTATTGGCAACAGTTACTGGCAGTACTGGATCAGCTCAATTGTC
PTTS058V-F	CAACTGGATCAGAATATAACAATTCCATGGTGGCAAGCTTATATTG
PTTS059-F	GAGCTGATCCAGTACTGCCAACTTTTAAAATTTCCAACAACAATCT
PTTS059-R	CAATATAAGCTTGCCACCATGGAATACCAATACTCAAAGATCTTG
PTTS059V-R	GTTGTTGGAAATTTTAAAAGTTGGCAGTACTGGATCAGCTCAATTG
PTTS059V-F	CAAGATCTTTGAGTATTGGTATTCCATGGTGGCAAGCTTATATTG
PTTS060-F	CAATTGAGCTGATCCAGTACTGCCTTCACAATCTTGTGTTGGTCTTG
PTTS060-R	CAATATAAGCTTGCCACCATGAAATTGAAGGTTAAGATCAGAAAAGC
PTTS060V-R	GACCAACACAAGATTGTGAAGGCAGTACTGGATCAGCTCAATTGTC
PTTS060V-F	GCTTTTCTGATCTTAACCTTCAATTTCATGGTGGCAAGCTTATATTG
PTTS061-F	GAGCTGATCCAGTACTGCCTGCAACGATATCTCTCAAAGATAATC
PTTS061-R	CAATATAAGCTTGCCACCATGGATGCTATTTGGGAATATTCAGTTG
PTTS061V-R	CTTTGAGAGATATCGTTGCAGGCAGTACTGGATCAGCTCAATTGTC
PTTS061V-F	CAACTGAATATTCCCAAATAGCATCCATGGTGGCAAGCTTATATTG
PTTS062-F	CAATATAAGCTTGCCACCATGACAATTCATCAATACTCAGTTGAAG
PTTS062-R	CAATTGAGCTGATCCAGTACTGCCTCTAGACAAACCTCTGATCAAC

PTTS062V-R	CTGAGTATTGATGAATTGTCATGGTGGCAAGCTTATATTGAATTTTC
PTTS062V-F	GTTGATCAGAGGTTTGTCTAGAGGCAGTACTGGATCAGCTCAATTG
PTTS065-F	CAATATAAGCTTGCCACCATGGAATGGAGATACTCTGTTCCAG
PTTS065-R	CAATTGAGCTGATCCAGTACTGCCAATTCTTAACATTTCAACAACC
PTTS065V-R	GAACAGAGTATCTCCATTCCATGGTGGCAAGCTTATATTGAATTTTC
PTTS065V-F	GAAATGTTAAGAATTGGCAGTACTGGATCAGCTCAATTGTCTGTTG
PTTS066-F	GCTGATCCAGTACTGCCAACTGTTAATTCACCCATACACAATCTC
PTTS066-R	CAATATAAGCTTGCCACCATGTGTACTATGGATGATCATACATCTG
PTTS066V-R	GATTGTGTATGGGTGAATTAACAGTTGGCAGTACTGGATCAGCTC
PTTS066V-F	CAGATGTATGATCATCCATAGTACACATGGTGGCAAGCTTATATTG
PTTS067-F	CAATATAAGCTTGCCACCATGTTATCTGATGATGAATTGTATCC
PTTS067-R	CAATTGAGCTGATCCAGTACTGCCAATTCTCAATGTTTCAGCCAAC
PTTS067V-R	CAATTCATCATCAGATAACATGGTGGCAAGCTTATATTGAATTTTC
PTTS067V-F	GTTGGCTGAAACATTGAGAATTGGCAGTACTGGATCAGCTCAATTG
PTTS070-F	GAGCTGATCCAGTACTGCCTAAACCCAAACCAATCAATAAAACTC
PTTS070-R	CAATATAAGCTTGCCACCATGGAAGATTTCCCAAACGTTTTCG
PTTS070V-R	GTTTTATTGATTGGTTTGGGTTTAGGCAGTACTGGATCAGCTC
PTTS070V-F	CGAAAACGTTTGGGAAATCTTCCATGGTGGCAAGCTTATATTG
PTTS074-F	CAATATAAGCTTGCCACCATGCCAACTTTTGCACAACCAGTTCCAG
PTTS074-R	GAGCTGATCCAGTACTGCCAGAAATCCACAATCTTCTTAAAACC
PTTS074V-R	CTGGTTGTGCAAAAGTTGGCATGGTGGCAAGCTTATATTGAATTTTC
PTTS074V-F	GGTTTTAAGAAGATTGTGGATTTCTGGCAGTACTGGATCAGCTC
PTTS078-F	CAATATAAGCTTGCCACCATGGATGCTCAATCTAGATCTTCACAATG
PTTS078-R	GAGCTGATCCAGTACTGCCAATCCACAACTTATTCAATAACAATC
PTTS078V-R	GAAGATCTAGATTGAGCATCCATGGTGGCAAGCTTATATTGAATTTTC
PTTS078V-F	GTTATTGAATAAGTTGTGGATTGGCAGTACTGGATCAGCTCAATTG
PTTS079-F	GAAAATTCAATATAAGCTTGCCACCATGGGTATTGCTGATGGTTTGAC
PTTS079-R	GAGCTGATCCAGTACTGCCAACAGACAATTTTTGAACCAATGCTTTC
PTTS079V-R	GTCAAACCATCAGCAATACCCATGGTGGCAAGCTTATATTGAATTTTC
PTTS079V-F	CATTGGTTCAAAAATTGTCTGTTGGCAGTACTGGATCAGCTCAATTG
PTTS080-F	CAATATAAGCTTGCCACCATGGATTACCAAAACATCCAAACAGAAC
PTTS080-R	GAGCTGATCCAGTACTGCCTCTAACCATTAATCTATGAATCAAC
PTTS080V-R	GTTTGGATGTTTTGGTAATCCATGGTGGCAAGCTTATATTGAATTTTC
PTTS080V-F	GATTCATAGATTAATGGTTAGAGGCAGTACTGGATCAGCTCAATTG
PTTS086-F	CAATATAAGCTTGCCACCATGGAAACTTTTGGTAAATTGGGTGAAG
PTTS086-R	CAATTGAGCTGATCCAGTACTGCCAATTCTCAACATTTCAACCAAC
PTTS086V-R	CAATTTACCAAAAGTTTCCATGGTGGCAAGCTTATATTGAATTTTC
PTTS086V-F	GTTGGTTGAAATGTTGAGAATTGGCAGTACTGGATCAGCTCAATTG
PTTS087-F	GAGCTGATCCAGTACTGCCAACTTTTAACAATTCTAACAAAGATTG
PTTS087-R	CAATATAAGCTTGCCACCATGGAATACAGATACTCTTACATGATCG
PTTS087V-R	CTTTGTTAGAATTGTTAAAAGTTGGCAGTACTGGATCAGCTCAATTG
PTTS087V-F	CGATCATGTAAGAGTATCTGTATTCCATGGTGGCAAGCTTATATTG
PTTS089-F	CAATATAAGCTTGCCACCATGCCATCTTTTTCAAGACCAGTTCCAG

PTTS089-R	GAGCTGATCCAGTACTGCCTGAAATCCACAATTTCTTCAAAACC
PTTS089V-R	CTGGTCTTGAAAAAGATGGCATGGTGGCAAGCTTATATTGAATTTTC
PTTS089V-F	GGTTTTGAAGAAATTGTGGATTTCAGGCAGTACTGGATCAGCTC
PTTS090-F	GAGCTGATCCAGTACTGCCAACTTGTAATCTTTTCAATAACAATCTC
PTTS090-R	CAATATAAGCTTGCCACCATGGCTGGTACTAAGAAATCTACTGCTAC
PTTS090V-R	GTTATTGAAAAGATTACAAGTTGGCAGTACTGGATCAGCTCAATTG
PTTS090V-F	GCAGTAGATTTCTTAGTACCAGCCATGGTGGCAAGCTTATATTG
PTTS092-F	GAAAATTCAATATAAGCTTGCCACCATGTTGCCAGATAACGAATTG
PTTS092-R	CAATTGAGCTGATCCAGTACTGCCAGAAATTCTCAATGTTTCAACC
PTTS092V-R	CAATTCGTTATCTGGCAACATGGTGGCAAGCTTATATTGAATTTTC
PTTS092V-F	GGTTGAAACATTGAGAATTTCTGGCAGTACTGGATCAGCTCAATTG
PTTS093-F	CAATATAAGCTTGCCACCATGCCATACTTCTCTAACCCATTGCCAAC
PTTS093-R	CAATTGAGCTGATCCAGTACTGCCAACCCACAATTTTCTCAAAAC
PTTS093V-R	GTTGGCAATGGGTTAGAGAAGTATGGCATGGTGGCAAGCTTATATTG
PTTS093V-F	GTTTTGAGAAAATTGTGGGTTGGCAGTACTGGATCAGCTCAATTG
PTTS097-F	GAAAATTCAATATAAGCTTGCCACCATGTTGCCATCTTCAAGAGATTC
PTTS097-R	CAATTGAGCTGATCCAGTACTGCCCAATTCTAATCTTTTCTTCATC
PTTS097V-R	GAATCTCTTGAAGATGGCAACATGGTGGCAAGCTTATATTGAATTTTC
PTTS097V-F	GATGAAGAAAAGATTAGAATTGGGCAGTACTGGATCAGCTCAATTG
PTTS100-F	GAGCTGATCCAGTACTGCCAACTTTTAACATTTCCATCATCAATC
PTTS100-R	CAATATAAGCTTGCCACCATGGCACCATCTTATGATCAACCATAC
PTTS100V-R	GATGATGGAAATGTTAAAAGTTGGCAGTACTGGATCAGCTCAATTG
PTTS100V-F	GTTGATCATAAGATGGTGCCATGGTGGCAAGCTTATATTGAATTTTC
PTTS103-R	CAATTGAGCTGATCCAGTACTGCCAACTTTTAACATTTCCAATAAC
PTTS103-F	GAAAATTCAATATAAGCTTGCCACCATGGATTACAAATTTTCTAC
PTTS103V-R	GTAGAAAATTTGTAATCCATGGTGGCAAGCTTATATTGAATTTTC
PTTS103V-F	GTTATTGGAAATGTTAAAAGTTGGCAGTACTGGATCAGCTCAATTG
PTTS106-R	CAATTGAGCTGATCCAGTACTGCCTTCAATCCACAATGATTCCATC
PTTS106-F	GAAAATTCAATATAAGCTTGCCACCATGTTATCTCATGAAGAATTG
PTTS106V-R	CAATTCTTCATGAGATAACATGGTGGCAAGCTTATATTGAATTTTC
PTTS106V-F	GATGGAATCATTGTGGATTGAAGGCAGTACTGGATCAGCTCAATTG
PTTS107-F	CAATATAAGCTTGCCACCATGGATCAATCACAATCTTTGTTGG
PTTS107-R	CAATTGAGCTGATCCAGTACTGCCCAAAGATAATTTCCACAACAAC
PTTS107V-R	CATCCAACAAAGATTGTGATTGATCCATGGTGGCAAGCTTATATTG
PTTS107V-F	GTTGTGGAAATTATCTTTGGGCAGTACTGGATCAGCTCAATTGTC
PTTS108-R	CAATTGAGCTGATCCAGTACTGCCTTCAGCTCTGTTCAAACCAAC
PTTS108-F	GAAAATTCAATATAAGCTTGCCACCATGGAATCTTTATCATACC
PTTS108V-R	GGTATGATAAAGATTCCATGGTGGCAAGCTTATATTGAATTTTC
PTTS108V-F	GTTGGTTTGAACAGAGCTGAAGGCAGTACTGGATCAGCTCAATTG
PTTS110-F	GAAAATTCAATATAAGCTTGCCACCATGGTTCCAACTTCATTATCTCC
PTTS110-R	CAATTGAGCTGATCCAGTACTGCCAACTTCCAATCTATGAACCAAC
PTTS110V-R	CTGGAGATAATGAAGTTGGAACCATGGTGGCAAGCTTATATTG
PTTS110V-F	GGTTCATAGATTGGAAGTTGGCAGTACTGGATCAGCTCAATTG

PTTS114-R	GAGCTGATCCAGTACTGCCAACTCTTAACAATTCCCATAAAAC
PTTS114-F	GAAAATTCAATATAAGCTTGCCACCATGGAATTTCAATATTCTAC
PTTS114V-R	GTAGAATATTGAAATTCCATGGTGGCAAGCTTATATTGAATTTTC
PTTS114V-F	GTTTTATGGGAATTGTTAAGAGTTGGCAGTACTGGATCAGCTC
PTTS116-R	GACAATTGAGCTGATCCAGTACTGCCAGAAACTTTTAACAATTCC
PTTS116-F	GAAAATTCAATATAAGCTTGCCACCATGAGATACAAATTTTCTAC
PTTS116V-R	GTAGAAAATTTGTATCTCATGGTGGCAAGCTTATATTGAATTTTC
PTTS116V-F	GGAATTGTTAAAAGTTTCTGGCAGTACTGGATCAGCTCAATTGTC
PTTS119-F	CAATATAAGCTTGCCACCATGATTTCTATTGCTCCATTCCCAGATG
PTTS119-R	CAATTGAGCTGATCCAGTACTGCCAACTTCCAATCTCATAACCAAC
PTTS119V-R	CTGGGAATGGAGCAATAGAAATCATGGTGGCAAGCTTATATTG
PTTS119V-F	GTTATGAGATTGGAAGTTGGCAGTACTGGATCAGCTCAATTGTC
PTTS122-R	CAATTGAGCTGATCCAGTACTGCCAATTTTCAATGTTTCTAAC
PTTS122-F	GAAAATTCAATATAAGCTTGCCACCATGGATTACAGAAATTCTTC
PTTS122V-R	GAAGAATTTCTGTAATCCATGGTGGCAAGCTTATATTGAATTTTC
PTTS122V-F	GTTAGAAACATTGAAAATTGGCAGTACTGGATCAGCTCAATTG
PTTS125-F	CAATATAAGCTTGCCACCATGGCTTCATTTTCTAGACCAGTTC
PTTS125-R	GAGCTGATCCAGTACTGCCTGAGATCCACAATTTTCTCAAAAC
PTTS125V-R	CTGGAACTGGTCTAGAAAATGAAGCCATGGTGGCAAGCTTATATTG
PTTS125V-F	GTTTTGAGAAAATTGTGGATCTCAGGCAGTACTGGATCAGCTCAATTG
PTTS127-R	GAGCTGATCCAGTACTGCCAATTCTTGCAAAAGCTCTCTTATC
PTTS127-F	CAATATAAGCTTGCCACCATGGCTTTTACTGGTTCTTTTTCAG
PTTS127V-R	CTGAAAAAGAACCAGTAAAAGCCATGGTGGCAAGCTTATATTG
PTTS127V-F	GATAAGAGAGCTTTTGCAAGAATTGGCAGTACTGGATCAGCTC
PTTS128-R	CAATTGAGCTGATCCAGTACTGCCAACTTCTAATCTATGAACC
PTTS128-F	GGTTCATAGATTAGAAGTTGGCAGTACTGGATCAGCTCAATTG
PTTS128V-R	GAAACAAATGTGGCAAAGCCATCATGGTGGCAAGCTTATATTG
PTTS128V-F	GGTTCATAGATTAGAAGTTGGCAGTACTGGATCAGCTCAATTG
PTTS129-R	GAGCTGATCCAGTACTGCCAACTCTCATCATTTCTAACATAAC
PTTS129-F	GAAAATTCAATATAAGCTTGCCACCATGGATTACAAATTTTCTAC
PTTS129V-R	GTAGAAAATTTGTAATCCATGGTGGCAAGCTTATATTGAATTTTC
PTTS129V-F	GTTATGTTAGAAATGATGAGAGTTGGCAGTACTGGATCAGCTC
PTTS130-R	GAGCTGATCCAGTACTGCCAATACAATGAATTAAAGTAAAAGAC
PTTS130-F	GAAAATTCAATATAAGCTTGCCACCATGGCTCATAAATATTCAAC
PTTS130V-R	GTTGAATATTTATGAGCCATGGTGGCAAGCTTATATTGAATTTTC
PTTS130V-F	GTCTTTTACTTTAATTCATTGTATTGGCAGTACTGGATCAGCTC
PTTS136-F	GAAAATTCAATATAAGCTTGCCACCATGGTTAAAGAATATGCTGTTCC
PTTS136-R	CAATTGAGCTGATCCAGTACTGCCAGATGCTTCCAATGCTCTC
PTTS136V-R	GGAACAGCATATTCTTTAACCATGGTGGCAAGCTTATATTGAATTTTC
PTTS136V-F	GAGAGCATTGGAAGCATCTGGCAGTACTGGATCAGCTCAATTG
173-ADH1-R	GAGATTAGAATTGGAAGTTTAAGAATTCGCGAATTTCTTATGATTTATG
173-PTTC051- F	ATAAGAAATTCGCGAATTCTTAAACTTCCAATTCTAATCTCTTTTGAAC
174-PTTC052-F	TAAGAAATTCGCGAATTCTTAAACTCTTAACAATTCTAAAATAACTTCC

174-ADH1- R	ATTTTAGAATTGTTAAGAGTTTAAGAATTCGCGAATTTCTTATGATTTATG
175-PTTC066-F	CATAAGAAATTCGCGAATTCTTATTAAACTGTTAATTCACCCATACACAATC
175-ADH1-R	GTATGGGTGAATTAACAGTTTAATAAGAATTCGCGAATTTCTTATGATTTATG
176-PTTC013-F	CATAAGAAATTCGCGAATTCTTACATTTCTCTCAATCTATCCAATAAAAATC
176-ADH1-R	TTGGATAGATTGAGAGAAATGTAAGAATTCGCGAATTTCTTATGATTTATG
177-PTTC021-F	CATAAGAAATTCGCGAATTCTTAATGACCCTTTTTACCTGGTTTTG
177-ADH1-R	CCAGGTAAAAAGGGTCATTAAGAATTCGCGAATTTCTTATGATTTATG
173-FDS-SC-F	CAAGGAGAAAAAACTATAGTCGACGCTCAATTGTCTGTTGAACAATTC
173-pGAL10-R	GTTCAACAGACAATTGAGCGTCGACTATAGTTTTTTCTCCTTGACG
173-CYC1-F	GTTGCTGCAAGAGATCATTAATCATGTAATTAGTTATGTCACGCTTAC
173-FDS-SC-R	GACATAACTAATTACATGATTAATGATCTCTTGCAGCAACTAATTCAC

Table S26. Details of plasmids used in this study.

Plasmids	Description	Reference
pGB315	p426gal derived, *URA*, T_{CYC1}-*ERG20*-P_{GAL1}-P_{GAL10}-*FgJ03939*-T_{ADH1}	(21)
pRC310	pGB315 derived, *URA*, P_{GAL10}-*PTTS001-GFPPS*-T_{ADH1}	This work
pRC311	pGB315 derived, *URA*, P_{GAL10}-*PTTS002-GFPPS*-T_{ADH1}	This work
pRC312	pGB315 derived, *URA*, P_{GAL10}-*PTTS003-GFPPS*-T_{ADH1}	This work
pRC313	pGB315 derived, *URA*, P_{GAL10}-*PTTS004-GFPPS*-T_{ADH1}	This work
pRC314	pGB315 derived, *URA*, P_{GAL10}-*PTTS005-GFPPS*-T_{ADH1}	This work
pRC315	pGB315 derived, *URA*, P_{GAL10}-*PTTS006-GFPPS*-T_{ADH1}	This work
pRC316	pGB315 derived, *URA*, P_{GAL10}-*PTTS007-GFPPS*-T_{ADH1}	This work
pRC317	pGB315 derived, *URA*, P_{GAL10}-*PTTS008-GFPPS*-T_{ADH1}	This work
pRC318	pGB315 derived, *URA*, P_{GAL10}-*PTTS009-GFPPS*-T_{ADH1}	This work
pRC319	pGB315 derived, *URA*, P_{GAL10}-*PTTS010-GFPPS*-T_{ADH1}	This work
pRC320	pGB315 derived, *URA*, P_{GAL10}-*PTTS013-GFPPS*-T_{ADH1}	This work
pRC321	pGB315 derived, *URA*, P_{GAL10}-*PTTS014-GFPPS*-T_{ADH1}	This work
pRC322	pGB315 derived, *URA*, P_{GAL10}-*PTTS016-GFPPS*-T_{ADH1}	This work
pRC323	pGB315 derived, *URA*, P_{GAL10}-*PTTS017-GFPPS*-T_{ADH1}	This work
pRC324	pGB315 derived, *URA*, P_{GAL10}-*PTTS021-GFPPS*-T_{ADH1}	This work
pRC325	pGB315 derived, *URA*, P_{GAL10}-*PTTS023-GFPPS*-T_{ADH1}	This work
pRC326	pGB315 derived, *URA*, P_{GAL10}-*PTTS024-GFPPS*-T_{ADH1}	This work
pRC327	pGB315 derived, *URA*, P_{GAL10}-*PTTS026-GFPPS*-T_{ADH1}	This work
pRC328	pGB315 derived, *URA*, P_{GAL10}-*PTTS027-GFPPS*-T_{ADH1}	This work
pRC329	pGB315 derived, *URA*, P_{GAL10}-*PTTS030-GFPPS*-T_{ADH1}	This work
pRC330	pGB315 derived, *URA*, P_{GAL10}-*PTTS033-GFPPS*-T_{ADH1}	This work
pRC331	pGB315 derived, *URA*, P_{GAL10}-*PTTS035-GFPPS*-T_{ADH1}	This work
pRC332	pGB315 derived, *URA*, P_{GAL10}-*PTTS036-GFPPS*-T_{ADH1}	This work
pRC333	pGB315 derived, *URA*, P_{GAL10}-*PTTS037-GFPPS*-T_{ADH1}	This work
pRC334	pGB315 derived, *URA*, P_{GAL10}-*PTTS039-GFPPS*-T_{ADH1}	This work
pRC335	pGB315 derived, *URA*, P_{GAL10}-*PTTS040-GFPPS*-T_{ADH1}	This work
pRC336	pGB315 derived, *URA*, P_{GAL10}-*PTTS041-GFPPS*-T_{ADH1}	This work

pRC337	pGB315 derived, *URA*, P_{GAL10}-*PTTS042-GFPPS*-T_{ADH1}	This work
pRC338	pGB315 derived, *URA*, P_{GAL10}-*PTTS043-GFPPS*-T_{ADH1}	This work
pRC339	pGB315 derived, *URA*, P_{GAL10}-*PTTS044-GFPPS*-T_{ADH1}	This work
pRC340	pGB315 derived, *URA*, P_{GAL10}-*PTTS045-GFPPS*-T_{ADH1}	This work
pRC341	pGB315 derived, *URA*, P_{GAL10}-*PTTS046-GFPPS*-T_{ADH1}	This work
pRC342	pGB315 derived, *URA*, P_{GAL10}-*PTTS047-GFPPS*-T_{ADH1}	This work
pRC343	pGB315 derived, *URA*, P_{GAL10}-*PTTS049-GFPPS*-T_{ADH1}	This work
pRC344	pGB315 derived, *URA*, P_{GAL10}-*PTTS050-GFPPS*-T_{ADH1}	This work
pRC345	pGB315 derived, *URA*, P_{GAL10}-*PTTS051-GFPPS*-T_{ADH1}	This work
pRC346	pGB315 derived, *URA*, P_{GAL10}-*PTTS052-GFPPS*-T_{ADH1}	This work
pRC347	pGB315 derived, *URA*, P_{GAL10}-*PTTS054-GFPPS*-T_{ADH1}	This work
pRC348	pGB315 derived, *URA*, P_{GAL10}-*PTTS058-GFPPS*-T_{ADH1}	This work
pRC349	pGB315 derived, *URA*, P_{GAL10}-*PTTS059-GFPPS*-T_{ADH1}	This work
pRC350	pGB315 derived, *URA*, P_{GAL10}-*PTTS060-GFPPS*-T_{ADH1}	This work
pRC351	pGB315 derived, *URA*, P_{GAL10}-*PTTS061-GFPPS*-T_{ADH1}	This work
pRC352	pGB315 derived, *URA*, P_{GAL10}-*PTTS062-GFPPS*-T_{ADH1}	This work
pRC353	pGB315 derived, *URA*, P_{GAL10}-*PTTS065-GFPPS*-T_{ADH1}	This work
pRC354	pGB315 derived, *URA*, P_{GAL10}-*PTTS066-GFPPS*-T_{ADH1}	This work
pRC355	pGB315 derived, *URA*, P_{GAL10}-*PTTS067-GFPPS*-T_{ADH1}	This work
pRC356	pGB315 derived, *URA*, P_{GAL10}-*PTTS070-GFPPS*-T_{ADH1}	This work
pRC357	pGB315 derived, *URA*, P_{GAL10}-*PTTS074-GFPPS*-T_{ADH1}	This work
pRC358	pGB315 derived, *URA*, P_{GAL10}-*PTTS078-GFPPS*-T_{ADH1}	This work
pRC359	pGB315 derived, *URA*, P_{GAL10}-*PTTS079-GFPPS*-T_{ADH1}	This work
pRC360	pGB315 derived, *URA*, P_{GAL10}-*PTTS080-GFPPS*-T_{ADH1}	This work
pRC361	pGB315 derived, *URA*, P_{GAL10}-*PTTS086-GFPPS*-T_{ADH1}	This work
pRC362	pGB315 derived, *URA*, P_{GAL10}-*PTTS087-GFPPS*-T_{ADH1}	This work
pRC363	pGB315 derived, *URA*, P_{GAL10}-*PTTS089-GFPPS*-T_{ADH1}	This work
pRC364	pGB315 derived, *URA*, P_{GAL10}-*PTTS090-GFPPS*-T_{ADH1}	This work
pRC365	pGB315 derived, *URA*, P_{GAL10}-*PTTS092-GFPPS*-T_{ADH1}	This work
pRC366	pGB315 derived, *URA*, P_{GAL10}-*PTTS093-GFPPS*-T_{ADH1}	This work

pRC367	pGB315 derived, *URA*, P_{GAL10}-*PTTS097-GFPPS*-T_{ADH1}	This work
pRC368	pGB315 derived, *URA*, P_{GAL10}-*PTTS100-GFPPS*-T_{ADH1}	This work
pRC369	pGB315 derived, *URA*, P_{GAL10}-*PTTS103-GFPPS*-T_{ADH1}	This work
pRC370	pGB315 derived, *URA*, P_{GAL10}-*PTTS106-GFPPS*-T_{ADH1}	This work
pRC371	pGB315 derived, *URA*, P_{GAL10}-*PTTS107-GFPPS*-T_{ADH1}	This work
pRC372	pGB315 derived, *URA*, P_{GAL10}-*PTTS108-GFPPS*-T_{ADH1}	This work
pRC373	pGB315 derived, *URA*, P_{GAL10}-*PTTS110-GFPPS*-T_{ADH1}	This work
pRC374	pGB315 derived, *URA*, P_{GAL10}-*PTTS114-GFPPS*-T_{ADH1}	This work
pRC375	pGB315 derived, *URA*, P_{GAL10}-*PTTS116-GFPPS*-T_{ADH1}	This work
pRC376	pGB315 derived, *URA*, P_{GAL10}-*PTTS119-GFPPS*-T_{ADH1}	This work
pRC377	pGB315 derived, *URA*, P_{GAL10}-*PTTS122-GFPPS*-T_{ADH1}	This work
pRC378	pGB315 derived, *URA*, P_{GAL10}-*PTTS125-GFPPS*-T_{ADH1}	This work
pRC379	pGB315 derived, *URA*, P_{GAL10}-*PTTS127-GFPPS*-T_{ADH1}	This work
pRC380	pGB315 derived, *URA*, P_{GAL10}-*PTTS128-GFPPS*-T_{ADH1}	This work
pRC381	pGB315 derived, *URA*, P_{GAL10}-*PTTS129-GFPPS*-T_{ADH1}	This work
pRC382	pGB315 derived, *URA*, P_{GAL10}-*PTTS130-GFPPS*-T_{ADH1}	This work
pRC383	pGB315 derived, *URA*, P_{GAL10}-*PTTS136-GFPPS*-T_{ADH1}	This work
pRC384	pGB315 derived, *URA*, T_{CYC1}-*GFPPS*-P_{GAL1}, P_{GAL10}-*PTTS052*-T_{ADH1}	This work
pRC385	pGB315 derived, *URA*, T_{CYC1}-*GFPPS*-P_{GAL1}, P_{GAL10}-*PTTS066*-T_{ADH1}	This work
pRC386	pGB315 derived, *URA*, T_{CYC1}-*GFPPS*-P_{GAL1}, P_{GAL10}-*PTTS013*-T_{ADH1}	This work
pRC387	pGB315 derived, *URA*, T_{CYC1}-*GFPPS*-P_{GAL1}, P_{GAL10}-*PTTS021*-T_{ADH1}	This work
pRC388	pGB315 derived, *URA*, T_{CYC1}-*GFPPS*-P_{GAL1}, P_{GAL10}-*PTTS051*-T_{ADH1}	This work

Table S27. Details of strains used in this study.

Strains	Relevant genotype	Reference
DH10B	E. coli D F-mcrA Δ(mrr-hsdRMS-mcrBC) Φ80dlacZ ΔM15 ΔlacX74 endA1 recA1 deoR Δ(ara,leu)7697 araD139 galU galK nupG rpsL λ^-	Invitrogen
CEN.PK2-1D	*Saccharomyces cerevisiae* MATalpha; his3D1; leu2-3_112; ura3-52; trp1-289; MAL2-8c; SUC2	EUROSCARF
YZL141	*S. cerevisiae*:: pGAL10-tHMG1	(21)
mRC310	*S. cerevisiae*:: P_{GAL10}-*tHMG1*; P_{GAL10}-*PTTS001-GFPPS*	This work
mRC311	*S. cerevisiae*:: P_{GAL10}-*tHMG1*; P_{GAL10}-*PTTS002-GFPPS*	This work
mRC312	*S. cerevisiae*:: P_{GAL10}-*tHMG1*; P_{GAL10}-*PTTS003 -GFPPS*	This work
mRC313	*S. cerevisiae*:: P_{GAL10}-*tHMG1*; P_{GAL10}-*PTTS004 -GFPPS*	This work
mRC314	*S. cerevisiae*:: P_{GAL10}-*tHMG1*; P_{GAL10}-*PTTS005 -GFPPS*	This work
mRC315	*S. cerevisiae*:: P_{GAL10}-*tHMG1*; P_{GAL10}-*PTTS006 -GFPPS*	This work
mRC316	*S. cerevisiae*:: P_{GAL10}-*tHMG1*; P_{GAL10}-*PTTS007 -GFPPS*	This work
mRC317	*S. cerevisiae*:: P_{GAL10}-*tHMG1*; P_{GAL10}-*PTTS008 -GFPPS*	This work
mRC318	*S. cerevisiae*:: P_{GAL10}-*tHMG1*; P_{GAL10}-*PTTS009 -GFPPS*	This work
mRC319	*S. cerevisiae*:: P_{GAL10}-*tHMG1*; P_{GAL10}-*PTTS010 -GFPPS*	This work
mRC320	*S. cerevisiae*:: P_{GAL10}-*tHMG1*; P_{GAL10}-*PTTS013 -GFPPS*	This work
mRC321	*S. cerevisiae*:: P_{GAL10}-*tHMG1*; P_{GAL10}-*PTTS014 -GFPPS*	This work
mRC322	*S. cerevisiae*:: P_{GAL10}-*tHMG1*; P_{GAL10}-*PTTS016 -GFPPS*	This work
mRC323	*S. cerevisiae*:: P_{GAL10}-*tHMG1*; P_{GAL10}-*PTTS017 -GFPPS*	This work
mRC324	*S. cerevisiae*:: P_{GAL10}-*tHMG1*; P_{GAL10}-*PTTS021 -GFPPS*	This work
mRC325	*S. cerevisiae*:: P_{GAL10}-*tHMG1*; P_{GAL10}-*PTTS023 -GFPPS*	This work
mRC326	*S. cerevisiae*:: P_{GAL10}-*tHMG1*; P_{GAL10}-*PTTS024 -GFPPS*	This work
mRC327	*S. cerevisiae*:: P_{GAL10}-*tHMG1*; P_{GAL10}-*PTTS026 -GFPPS*	This work
mRC328	*S. cerevisiae*:: P_{GAL10}-*tHMG1*; P_{GAL10}-*PTTS027 -GFPPS*	This work
mRC329	*S. cerevisiae*:: P_{GAL10}-*tHMG1*; P_{GAL10}-*PTTS030 -GFPPS*	This work
mRC330	*S. cerevisiae*:: P_{GAL10}-*tHMG1*; P_{GAL10}-*PTTS033 -GFPPS*	This work
mRC331	*S. cerevisiae*:: P_{GAL10}-*tHMG1*; P_{GAL10}-*PTTS035 -GFPPS*	This work
mRC332	*S. cerevisiae*:: P_{GAL10}-*tHMG1*; P_{GAL10}-*PTTS036 -GFPPS*	This work
mRC333	*S. cerevisiae*:: P_{GAL10}-*tHMG1*; P_{GAL10}-*PTTS037 -GFPPS*	This work

mRC334	*S. cerevisiae*:: P_{GAL10}-*tHMG1; P_{GAL10}-PTTS039 -GFPPS*	This work
mRC335	*S. cerevisiae*:: P_{GAL10}-*tHMG1; P_{GAL10}-PTTS040 -GFPPS*	This work
mRC336	*S. cerevisiae*:: P_{GAL10}-*tHMG1; P_{GAL10}-PTTS041 -GFPPS*	This work
mRC337	*S. cerevisiae*:: P_{GAL10}-*tHMG1; P_{GAL10}-PTTS042 -GFPPS*	This work
mRC338	*S. cerevisiae*:: P_{GAL10}-*tHMG1; P_{GAL10}-PTTS043 -GFPPS*	This work
mRC339	*S. cerevisiae*:: P_{GAL10}-*tHMG1; P_{GAL10}-PTTS044 -GFPPS*	This work
mRC340	*S. cerevisiae*:: P_{GAL10}-*tHMG1; P_{GAL10}-PTTS045 -GFPPS*	This work
mRC341	*S. cerevisiae*:: P_{GAL10}-*tHMG1; P_{GAL10}-PTTS046 -GFPPS*	This work
mRC342	*S. cerevisiae*:: P_{GAL10}-*tHMG1; P_{GAL10}-PTTS047 -GFPPS*	This work
mRC343	*S. cerevisiae*:: P_{GAL10}-*tHMG1; P_{GAL10}-PTTS049 -GFPPS*	This work
mRC344	*S. cerevisiae*:: P_{GAL10}-*tHMG1; P_{GAL10}-PTTS050 -GFPPS*	This work
mRC345	*S. cerevisiae*:: P_{GAL10}-*tHMG1; P_{GAL10}-PTTS051 -GFPPS*	This work
mRC346	*S. cerevisiae*:: P_{GAL10}-*tHMG1; P_{GAL10}-PTTS052 -GFPPS*	This work
mRC347	*S. cerevisiae*:: P_{GAL10}-*tHMG1; P_{GAL10}-PTTS054 -GFPPS*	This work
mRC348	*S. cerevisiae*:: P_{GAL10}-*tHMG1; P_{GAL10}-PTTS058 -GFPPS*	This work
mRC349	*S. cerevisiae*:: P_{GAL10}-*tHMG1; P_{GAL10}-PTTS059 -GFPPS*	This work
mRC350	*S. cerevisiae*:: P_{GAL10}-*tHMG1; P_{GAL10}-PTTS060 -GFPPS*	This work
mRC351	*S. cerevisiae*:: P_{GAL10}-*tHMG1; P_{GAL10}-PTTS061 -GFPPS*	This work
mRC352	*S. cerevisiae*:: P_{GAL10}-*tHMG1; P_{GAL10}-PTTS062 -GFPPS*	This work
mRC353	*S. cerevisiae*:: P_{GAL10}-*tHMG1; P_{GAL10}-PTTS065 -GFPPS*	This work
mRC354	*S. cerevisiae*:: P_{GAL10}-*tHMG1; P_{GAL10}-PTTS066 -GFPPS*	This work
mRC355	*S. cerevisiae*:: P_{GAL10}-*tHMG1; P_{GAL10}-PTTS067 -GFPPS*	This work
mRC356	*S. cerevisiae*:: P_{GAL10}-*tHMG1; P_{GAL10}-PTTS070 -GFPPS*	This work
mRC357	*S. cerevisiae*:: P_{GAL10}-*tHMG1; P_{GAL10}-PTTS074 -GFPPS*	This work
mRC358	*S. cerevisiae*:: P_{GAL10}-*tHMG1; P_{GAL10}-PTTS078 -GFPPS*	This work
mRC359	*S. cerevisiae*:: P_{GAL10}-*tHMG1; P_{GAL10}-PTTS079 -GFPPS*	This work
mRC360	*S. cerevisiae*:: P_{GAL10}-*tHMG1; P_{GAL10}-PTTS080 -GFPPS*	This work
mRC361	*S. cerevisiae*:: P_{GAL10}-*tHMG1; P_{GAL10}-PTTS086 -GFPPS*	This work
mRC362	*S. cerevisiae*:: P_{GAL10}-*tHMG1; P_{GAL10}-PTTS087 -GFPPS*	This work
mRC363	*S. cerevisiae*:: P_{GAL10}-*tHMG1; P_{GAL10}-PTTS089 -GFPPS*	This work

mRC364	*S. cerevisiae*:: P_{GAL10}-*tHMG1; P_{GAL10}-PTTS090 -GFPPS*	This work
mRC365	*S. cerevisiae*:: P_{GAL10}-*tHMG1; P_{GAL10}-PTTS092 -GFPPS*	This work
mRC366	*S. cerevisiae*:: P_{GAL10}-*tHMG1; P_{GAL10}-PTTS093 -GFPPS*	This work
mRC367	*S. cerevisiae*:: P_{GAL10}-*tHMG1; P_{GAL10}-PTTS097 -GFPPS*	This work
mRC368	*S. cerevisiae*:: P_{GAL10}-*tHMG1; P_{GAL10}-PTTS100 -GFPPS*	This work
mRC369	*S. cerevisiae*:: P_{GAL10}-*tHMG1; P_{GAL10}-PTTS103 -GFPPS*	This work
mRC370	*S. cerevisiae*:: P_{GAL10}-*tHMG1; P_{GAL10}-PTTS106 -GFPPS*	This work
mRC371	*S. cerevisiae*:: P_{GAL10}-*tHMG1; P_{GAL10}-PTTS107 -GFPPS*	This work
mRC372	*S. cerevisiae*:: P_{GAL10}-*tHMG1; P_{GAL10}-PTTS108; GFPPS*	This work
mRC373	*S. cerevisiae*:: P_{GAL10}-*tHMG1; P_{GAL10}-PTTS110; GFPPS*	This work
mRC374	*S. cerevisiae*:: P_{GAL10}-*tHMG1; P_{GAL10}-PTTS114; GFPPS*	This work
mRC375	*S. cerevisiae*:: P_{GAL10}-*tHMG1; P_{GAL10}-PTTS116; GFPPS*	This work
mRC376	*S. cerevisiae*:: P_{GAL10}-*tHMG1; P_{GAL10}-PTTS119; GFPPS*	This work
mRC377	*S. cerevisiae*:: P_{GAL10}-*tHMG1; P_{GAL10}-PTTS122; GFPPS*	This work
mRC378	*S. cerevisiae*:: P_{GAL10}-*tHMG1; P_{GAL10}-PTTS125; GFPPS*	This work
mRC379	*S. cerevisiae*:: P_{GAL10}-*tHMG1; P_{GAL10}-PTTS127; GFPPS*	This work
mRC380	*S. cerevisiae*:: P_{GAL10}-*tHMG1; P_{GAL10}-PTTS128; GFPPS*	This work
mRC381	*S. cerevisiae*:: P_{GAL10}-*tHMG1; P_{GAL10}-PTTS129; GFPPS*	This work
mRC382	*S. cerevisiae*:: P_{GAL10}-*tHMG1; P_{GAL10}-PTTS130; GFPPS*	This work
mRC383	*S. cerevisiae*:: P_{GAL10}-*tHMG1; P_{GAL10}-PTTS136; GFPPS*	This work
mRC384	*S. cerevisiae*:: P_{GAL10}-*tHMG1; P_{GAL10}-PTTS052; P_{GAL1}-GFPPS*	This work
mRC385	*S. cerevisiae*:: P_{GAL10}-*tHMG1; P_{GAL10}-PTTS066; P_{GAL1}-GFPPS*	This work
mRC386	*S. cerevisiae*:: P_{GAL10}-*tHMG1; P_{GAL10}-PTTS013; P_{GAL1}-GFPPS*	This work
mRC387	*S. cerevisiae*:: P_{GAL10}-*tHMG1; P_{GAL10}-PTTS021; P_{GAL1}-GFPPS*	This work
mRC388	*S. cerevisiae*:: P_{GAL10}-*tHMG1; P_{GAL10}-PTTS051; P_{GAL1}-GFPPS*	This work

References:

1. P. Pracht, F. Bohle, S. Grimme, Automated exploration of the low-energy chemical space with fast quantum chemical methods. *Physical Chemistry Chemical Physics* **22**, 7169-7192 (2020).
2. M. Frisch *et al.* (2016) Gaussian 16. (Gaussian, Inc. Wallingford, CT).
3. N. s. Grimblat, M. M. Zanardi, A. M. Sarotti, Beyond DP4: an improved probability for the stereochemical assignment of isomeric compounds using quantum chemical calculations of NMR shifts. *The Journal of organic chemistry* **80**, 12526-12534 (2015).
4. T. Lu, F. Chen, Multiwfn: a multifunctional wavefunction analyzer. *Journal of computational chemistry* **33**, 580-592 (2012).
5. A. Minami *et al.*, Identification and functional analysis of brassicicene C biosynthetic gene cluster in Alternaria brassicicola. *Bioorg Med Chem Lett* **19**, 870-874 (2009).
6. Z. Quan, J. S. Dickschat, Biosynthetic gene cluster for asperterpenols A and B and the cyclization mechanism of asperterpenol A synthase. *Org. Lett.* **22**, 7522-7555 (2020).
7. R. Chiba, A. Minami, K. Gomi, H. Oikawa, Identification of ophiobolin F synthase by a genome mining approach: a sesterterpene synthase from Aspergillus clavatus. *Org. Lett.* **15**, 594–597 (2013).
8. K. Narita *et al.*, Focused genome mining of structurally related sesterterpenes: enzymatic formation of enantiomeric and diastereomeric products. *Org. Lett.* **19**, 6696-6699 (2017).
9. A. Tazawa *et al.*, Total biosynthesis of brassicicenes: identification of a key enzyme for skeletal diversification. *Org. Lett.* **20**, 6178-6182 (2018).
10. G. Bian *et al.*, A Clade II-D fungal chimeric diterpene synthase from Colletotrichum gloeosporioides produces dolasta-1(15),8-diene. *Angew. Chem. Int. Ed.* **57**, 15887-15890 (2018).
11. Y. Matsuda *et al.*, Astellifadiene: structure determination by NMR spectroscopy and crystalline sponge method, and elucidation of its biosynthesis. *Angew. Chem. Int. Ed.* **55**, 5785-5788 (2016).
12. M. Okada *et al.*, Genome-based discovery of an unprecedented cyclization mode in fungal sesterterpenoid biosynthesis. *J. Am. Chem. Soc.* **138**, 10011-10018 (2016).
13. Y. Matsuda, T. Mitsuhashi, Z. Quan, I. Abe, Molecular basis for stellatic acid biosynthesis: a genome mining approach for discovery of sesterterpene synthases. *Org. Lett.* **17**, 4644-4647 (2015).
14. B. Qin *et al.*, An unusual chimeric diterpene synthase from Emericella variecolor and Its functional conversion into a sesterterpene synthase by domain swapping. *Angew. Chem. Int. Ed.* **55**, 1658-1661 (2016).
15. G. Bian *et al.*, Releasing the potential power of terpene synthases by a robust precursor supply platform. *Metab. Eng.* **42**, 1-8 (2017).
16. Y. Ye *et al.*, Genome mining for sesterterpenes using bifunctional terpene synthases reveals a unified Intermediate of di/sesterterpenes. *J. Am. Chem. Soc.* **137**, 11846-11853 (2015).
17. T. Toyomasu *et al.*, Fusicoccins are biosynthesized by an unusual chimera diterpene synthase in fungi. *Proc. Natl. Acad. Sci. USA.* **104**, 3084-3088 (2007).
18. T. Toyomasu *et al.*, Biosynthetic gene-based secondary metabolite screening: a new

diterpene, methyl phomopsenonate, from the fungus Phomopsis amygdali. *J. Org. Chem.* **74**, 1541-1548 (2009).

19. T. Mitsuhashi, J. Rinkel, M. Okada, I. Abe, J. S. Dickschat, Mechanistic characterization of two chimeric sesterterpene synthases from Penicillium. *Chem. Eur. J.* **23** (2017).
20. T. Mitsuhashi *et al.*, Crystalline sponge method enabled the Investigation of a prenyltransferase-terpene synthase chimeric enzyme, whose product exhibits broadened NMR signals. *Org. Lett.* **20**, 5606-5609 (2018).
21. G. K. Bian *et al.*, Metabolic engineering-based rapid characterization of a sesquiterpene cyclase and the skeletons of fusariumdiene and fusagramineol from Fusarium graminearum. *Org. Lett.* **20**, 1626-1629 (2018).
